# In-vacuum active colour sensor and wireless communication across a vacuum-air interface

**DOI:** 10.1038/s41598-020-80501-z

**Published:** 2021-01-14

**Authors:** Osamu Sakai, Takayuki Kitagawa, Keiji Sakurai, Go Itami, Shigeyuki Miyagi, Kazuyuki Noborio, Kohshi Taguchi

**Affiliations:** 1 Department of Electronic Systems Engineering, The University of Shiga Prefecture, 2500 Hassaka-cho, Hikone, Shiga 522-8533 Japan; 2 Regional ICT Research Center for Human, Industry and Future, The University of Shiga Prefecture, 2500 Hassaka-cho, Hikone, Shiga 522-8533 Japan; 3 R&D Department, Sakigake Semiconductor Co., Ltd, 50 Onmaeda-cho, Nishishichijo, Shimogyo-ku, Kyoto, 600-8897 Japan

**Keywords:** Electrical and electronic engineering, Information technology

## Abstract

In situ sensing with wireless digital-data transfer is a potential processing scheme that works very closely to the location of an event monitored by a sensor and converts the sensor’s raw output into digitized and informative small-volume bits, as suggested by recent proposals for edge computing and the Internet of Things (IoT). Colour perception may be a target of in situ sensor data acquisition; however, in contrast to from other sensing devices, colour sensors that detect visible light signals are usually located away from light-emitting sources, collecting light transmitting through the space and attenuating it in some manner. For example, in a vacuum chamber whose gas pressure is much less than the ambient atmosphere in which the sensors usually work, there are many veiled light sources, such as discharge plasma, for various industrial purposes including nanoscale manufacturing. In this study, we designed an in-vacuum colour sensor that can work with analogue-to-digital conversion and transfer data by wireless communication; this sensor is active in a low-pressure plasma chamber, detecting light signals and transferring them to a personal computer located outside the vacuum chamber. In addition to detecting lights with controlled spectra from outside successfully, we achieved complete operation of our in-vacuum active sensor for plasma emissions generated at 100 Pa. Comparing the signals with data from simultaneous monitoring by a monochromator, we established that the recorded signals arose from the plasma, confirming successful direct detection of low-pressure plasma emissions without any filtering effects between the sensor and the target object.

## Introduction

When physical and chemical events take place at a given spatial position, observation at the exact position reveals essential elements of the events qualitatively and quantitatively without any loss of origin information. However, in scientific diagnostics, observation points are often distant from the position of the original events, which makes signal attenuation and noise invasion inevitable; thus, accurate calibration and guaranteed reproducibility of the monitored quantities from sensors is difficult^1–3^. In optical diagnostics, absorption typically occurs, and is quantitatively defined using absorption coefficients, unless the propagation path is in a vacuum^4,5^. Another typical signal depression is caused by light reflection at the interface between media with different refractive indices or wave impedances, and complete matching with no reflection is impossible in most cases without an elaborate interface design^5,6^. When a given light medium is dispersive in space with its characteristic length of refractive-index variation larger than the wavelength, the WKB (Wentzel-Kramers-Brillouin) theory predicts amplitude modulation^7^, which is another reason why the observed light intensity differs from the original.

Edge computing enables us to obtain rich information on a sensor’s monitored targets and micro-processing unit, which is located in the vicinity of event occurrence; the components and integration design of edge computing devices are being widely explored from various points of view^8–12^. In scientific diagnostics, this computing concept introduces at least two suggestions to conventional sensor technology. First, by setting a sensor closer to an object that possesses or emits a target signal, analogue-to-digital (A/D) data conversion in an in situ edge-computing sensor is a promising means of improving data accuracy; analogue values are directly affected by ambient noises, whereas digital data are more robust since they are not composed of continuous analogue levels but comprises bit information that is available after digitization in a micro-processing unit. Second, a wireless communication module is easily installed in a sensor device, and the digital data after A/D conversion can be transferred to a data storage device such as a personal computer located far away.

We also designed our in situ sensor for robustness in low-pressure operation, which is an abnormal and very unlikely situation for classical sensors used in daily life. Our goal is to design a sensor for operation inside the vacuum chambers, because parameters of events inside the chambers are usually recorded using probing methods that are classically limited to analogue sensing and tend to suffer from noise interference. For instance, an optical probing device used for diagnostics of plasma in low-pressure vacuum chambers usually operates from outside through glass windows^3,13–15^. Light emissions from plasma are spatially integrated, leading to low spatial resolution^3^, and attenuated through the plasma itself and the window; ambient background noise easily sneaks into the signals detected by outside sensors. If we include robustness against low-pressure conditions, mainly by removing the possibilities of evaporation and/or explosion of constituents^16^ in the hardware components, then we can use these sensors inside vacuum chambers. When we directly detect light emissions from plasma, signals are free from the attenuation and contamination of background noise, while spatial signal integration is minimized.

In this context, our proposed scheme shares several points with the Internet of Things (IoT)^17–20^. Due to recent progress in IoT technology, large amounts of data are accumulated and available in healthcare systems, environmental monitoring systems, structural health monitoring systems, etc. This IoT technology basically handles the connections of things to the Internet or wireless communication networks. Our sensors are similar to IoT devices such as advanced sensors and their data acquisition systems^2^. In our case, we focus on the extension of sensor locations to an extreme environmental condition. Similar concepts were partially achieved in space science for satellites and planet probes^21–24^, but the expense and complexity of these probes are too specific for application to daily activities or for large-scale factory manufacturing.

In this study, we designed an in-vacuum active optical sensor that is capable of in situ computing and installed it in a vacuum chamber to detect light signals. The sensor consists of an integrated photodiode sensor with red (R), green (G), and blue (B) sensitivities and interintegrated circuit outputs, a reduced-instruction-set-computer (RISC) single-chip microcontroller, and a Bluetooth module (IEEE 802.15.1)^25^, all of which are commercially available at fairly low cost. Using this sensor at approximately 30 Pa in a vacuum chamber, we successfully monitored RGB intensities in the visible light range while recording the data to a personal computer outside the chamber (in ambient air at atmospheric pressure). After plasma ignition at 100 Pa of Ar, we observed direct optical signals from plasma emissions, and the detected values were considered reasonable responses based on the external parameter settings. The detected signals, completely without filtering effects in their output numerals, included information on the excited states of the Ar gas in the plasma region, which is indicated in an energy-level network diagram.

## Sensor operation in low-pressure vacuum chamber

**Figure 1:**
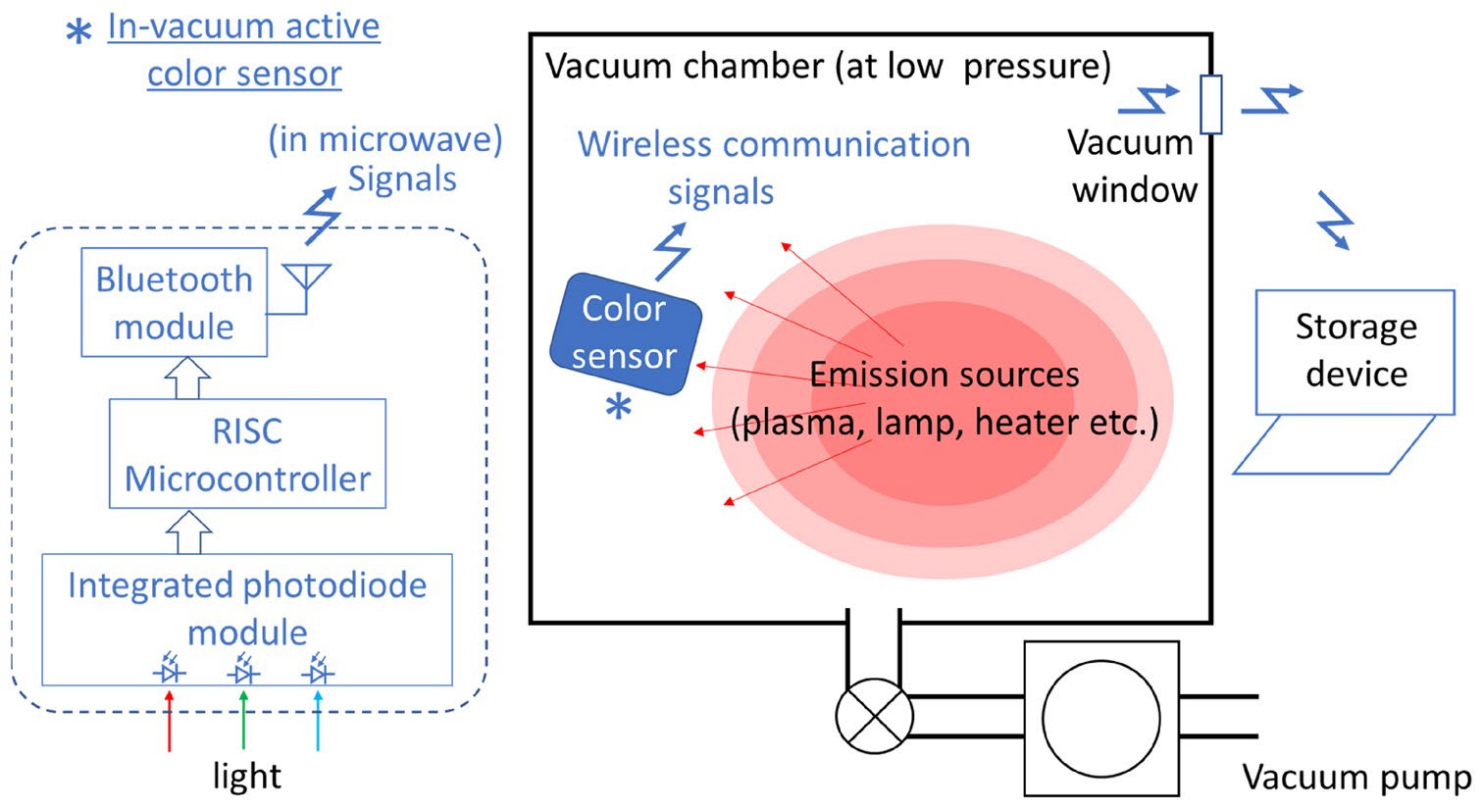
Conceptual view of edge-computing and wireless-communication system used in this study with a block diagram of the electronics in the colour sensor.

The in-vacuum active sensor possesses RGB-signal sensitivities, computing capability, a wireless communication function, and robustness at low pressure, as shown in Fig. 1 and described in the “Methods” section. In brief, an integrated photodiode array monitors the three colour components and creates three digital numeral outputs after A/D conversion, and the RISC microcontroller handles these outputs and sends them via Bluetooth standard wireless communication to a master personal computer that requests data transfer from the sensor. We stress that these circuit components for the vacuum chamber operation are commercially available at low cost without the need for custom orders. All the circuit components are in a metallic (aluminium) container with one side open for light introduction and another for wireless communication. Accurate calibration of these RGB outputs is completed after the procedure, and we had previously developed a supervised-learning calibration method using more than 1,600 colour samples^26^. However, we used these raw RGB outputs for comparison with wavelength spectra, as described below.

We installed sensors in two different setups in the same vacuum chamber; the lower part was made of stainless steel, whereas the upper part was a fused-quartz vessel (see details in the “Methods” section and Ref.^27^). For confirmation of invariances in the electronic device functions at low pressure, we used the glass-vessel part with no colour and enough transparency to allow us to test both the light detection and wireless communication. The glass vessel had a 100-mm outer diameter, larger than that in our previous experiment^27^. In the lower part, a columnar discharge electrode was set on the bottom with a metal-mesh-facing grounded electrode (see “Methods”).

**Figure 2:**
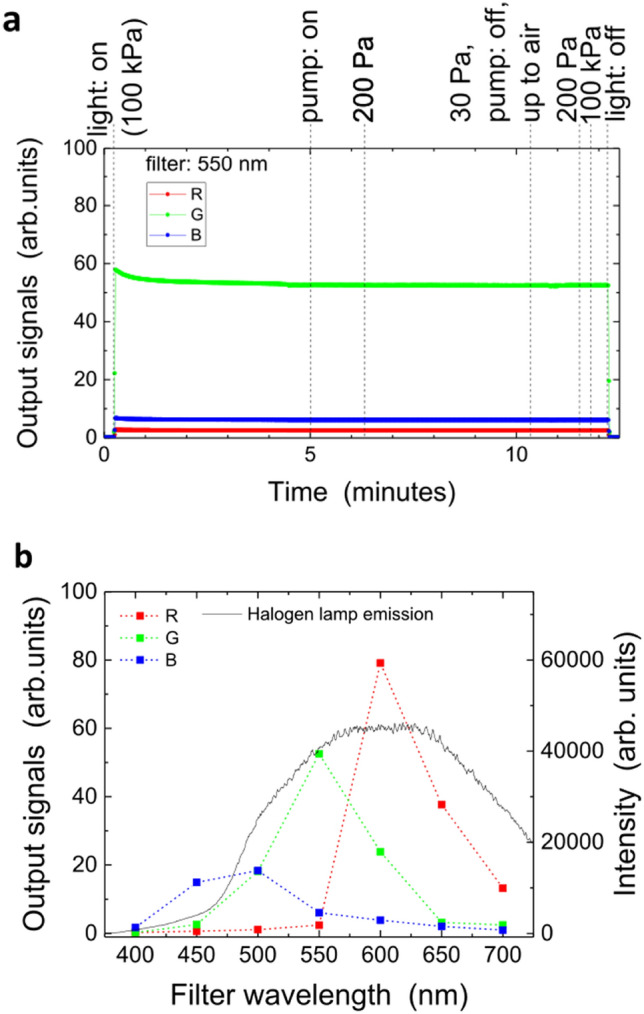
Output signals of the sensor when light originates from the halogen lamp located outside the chamber and enters the sensor inlet through the glass vessel. **(a)** Time evolution of the signals detected by the optical sensor located in the pressure-varying chamber. A bandpass filter with a centre wavelength of 550 nm is inserted on the outlet of the optical fibre that guides light from the lamp. **(b)** Signals of the R, G, and B components as a function of the centre wavelength bandpass filter. The halogen lamp emission spectrum detected by the monochromator is also shown. The values shown here are approximately 200 Pa, although they are invariant, as shown in **(a)**, regardless of pressure.

After installing the sensor above the grounded electrode and pumping the gas to 30 Pa, we tested for light detection and wireless communication as described below. This pressure is similar to or lower than that used for the plasma-enhanced chemical vapor deposition of thin Si films^28^ but higher than that for dry etching for trench formation in Si-oriented thin films^29,30^. Figure 2a shows signals transferred to the storage device outside the chamber, where visible lights were controlled by a halogen-lamp light source (Megalight100, SCHOTT Japan Corp.) externally installed above the glass vessel with wavelength bandpass filters (TS OD 4, Edmund Optics Ltd.). During the total operation period, the detected signals remained constant except for a slight reduction in the transient intensity of the light source during its start-up phase, indicating that the sensor suffered from neither a functional disorder nor a deterioration in signal level. We also confirmed that there were no effects of possible outgas from the sensors following the successful invariant operation at low pressure; no change in pressure trend occurred after pumping began or during the phases in which the pressure was decreased to 30 Pa or elevated up to that of air. This indicates that the electronic circuits, including the three chips of the integrated circuits, were live without any functional degradation during the operation of the vacuum pump. If any enclosed gas bubbles remained in the chips, they would have exploded at low pressure and might have damaged the fine circuit patterns, but this was not the case. Another concern is vapor evaporation of some parts of the circuits or other protection layers, but we confirmed sufficient robustness of our device because, as indicated above, we observed neither deterioration in sensor output nor abnormal pressure elevation during our tests.

Figure 2b shows the wavelength sensitivities of the R, G, and B sensor outputs by changing the centre wavelength of the bandpass filters. Although the range of each output has its own wavelength dependence, rough estimations suggest that the R component corresponds to lights from 600 to 700 nm, the G component from 500 to 600 nm, and the B component from 400 to 500 nm.

## Direct detection of plasma light emissions

Plasma in an electric discharge emits visible/ultraviolet/infrared lights spontaneously according to the excited energy levels of the included gases. Many articles have reported the results of optical diagnostics of plasma emissions obtained thus far, and almost all measurements were performed through layers or media such as vacuum windows, optical fibres, optical lenses/filters, and gas layers outside the plasma regions^13–15^. Careful preparation and arrangement of these layers can assure suitable (or equivalent) transparency for light. However, we cannot completely remove the possibility of signal degradation arising from surface reflections or wavelength dispersions of the optical components^3–7^ (see “Methods”). In addition, gas layers may induce self-absorption that reduces the original light intensity^31^. Direct measurements with optical sensors placed inside or in proximity to the plasma are favourable for the elimination of these concerns, and were considered for our proposed system.

**Figure 3:**
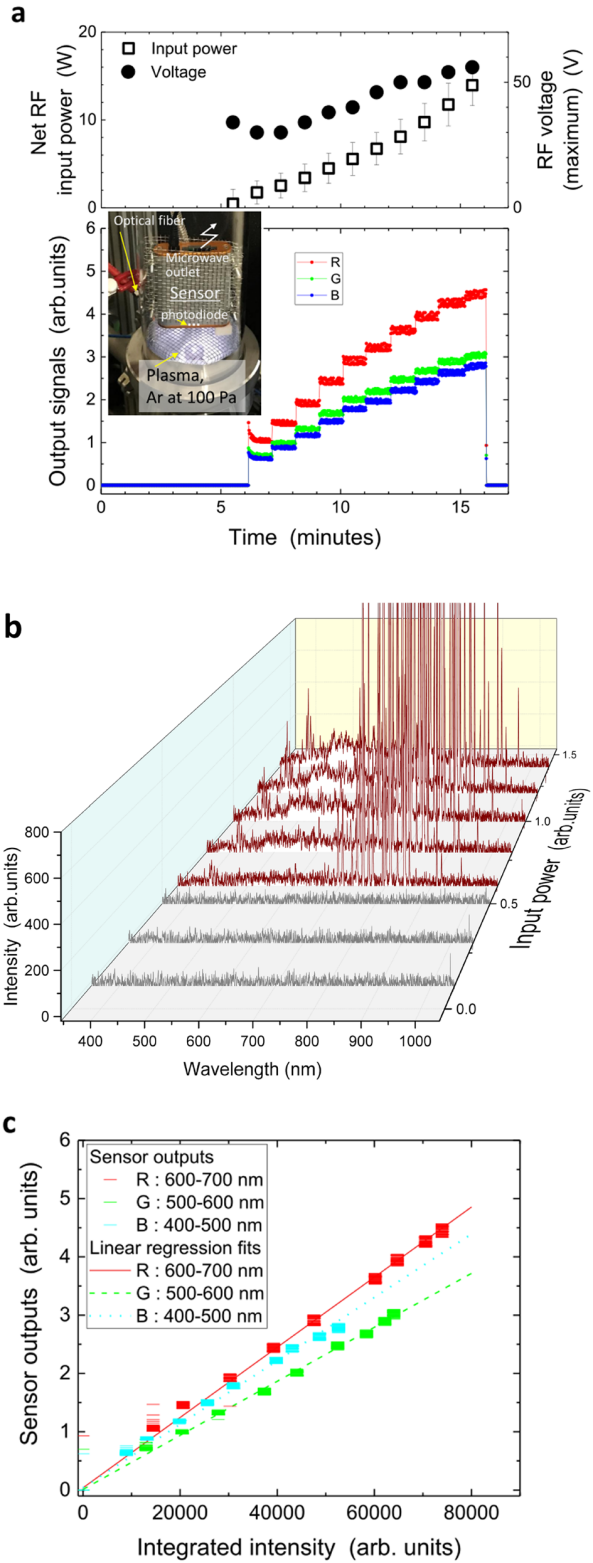
Detection of plasma emissions at 100 Pa of Ar. **(a)** Time evolutions of signals of averaged radiofrequency (RF, 13.56 MHz) net input power for plasma generation with the corresponding peak RF amplitude (upper side) and R, G, and B outputs of the sensor located in the vicinity of the plasma region (lower side), recorded on a personal computer located outside the vacuum chamber. Input power is linearly elevated every minute, and the power at level six ignites plasma. The inset photograph shows the outlook of the vacuum chamber used here and the visible plasma image. **(b)** Emission spectra of plasma monitored by monochromator to which optical fibre transmits lights from other end located just outside of glass vessel. Spectrum examples before plasma ignition are in grey, whereas those after plasma ignition are in brown, and lower-level signals are enlarged to focus on intensities in the visible light range. **(c)** Comparison of sensor output signals with integrated intensities of spectra monitored by monochromator. The signals used here are shown in **(a)**.

To perform our experiments with the colour sensor placed in the plasma space at low pressure, we installed the sensor upside down on the case, as shown in Fig. 2 (see “Methods”), with plasma generated on the lower side of the grounded mesh electrode and the plasma emissions moving along a direct path to the sensor inlet surface (the distance from the edge of the plasma was only 10 mm). To confirm that the plasma signals are distinguished from the background level (which was zero for all R, G, and B colour components), we adjusted the radiofrequency (RF) input power for plasma generation stepwise, by varying every 0.01-V output voltage of the RF signal generator and observed the corresponding responses. Figure 3a shows the detected signals over time. The signals started up at the sixth step, which was exactly the same time of the plasma ignition and demonstrated the same stepwise behaviour as that of the input power.

Before confirming the accuracy of the sensor signals, we investigated possible analogue noise signals on the signal processing unit. When we triggered analogue signals in a vacuum chamber, transmitted them to the storage device, and converted them to digital numerals, significant levels of noise invaded the estimated signal values. Figure 3a shows the RF voltage detected on the transmission line established between the RF amplifier and the powered electrode. This voltage is approximately the maximum level of the electric potential in the location of the sensor in this experiment. The voltage on the powered electrode, which was the ending point of the RF transmission line, was similar to or somewhat enhanced from the observed amplitude due to the mixing of forward and reflected RF powers. The plasma potential was similar to this externally-applied voltage because a sheath was mainly created on the surface of the grounded electrode. The mesh structure of the grounded electrode may sufficiently suppress this sheath potential, but part of it reaches the sensor since plasma leaked through the mesh holes (3.0 by 3.0 mm^2^). These considerations and findings imply that this in situ sensor significantly suppressed the noise effects created by the A/D conversion in the edge computing function; otherwise, the analogue data detected on the sensor would include RF noise attacking the signal transmission paths and might make the signals ambiguous.

To specify the sources of these sensor output signals, we simultaneously detected wavelength spectra of visible lights picked up by the optical fibre on the outer surface of the glass vessel, on the air side, and monitored it with a monochromator (USB4000, Ocean Optics Ltd.). As shown in Fig. 3b, the main emissions from the Ar plasma were in the infrared-ray range, but we also observed many spectra in the visible light range. These spectra indicate gradual changes in intensities in a manner similar to those of the elevated power levels for plasma generation. For a rough comparison, we plotted each output component of our in situ colour sensor as a function of the integrated values in the corresponding wavelength ranges (R for the range from 600 to 700 nm, G for that from 500 to 600 nm, and B for that from 400 to 500 nm (Fig. 3c). All dependences are clearly along one line of linear regression. This coincidentally confirmed that the output signals of the sensor successfully reflected Ar plasma emissions in this low-pressure in situ situation.

**Figure 4:**
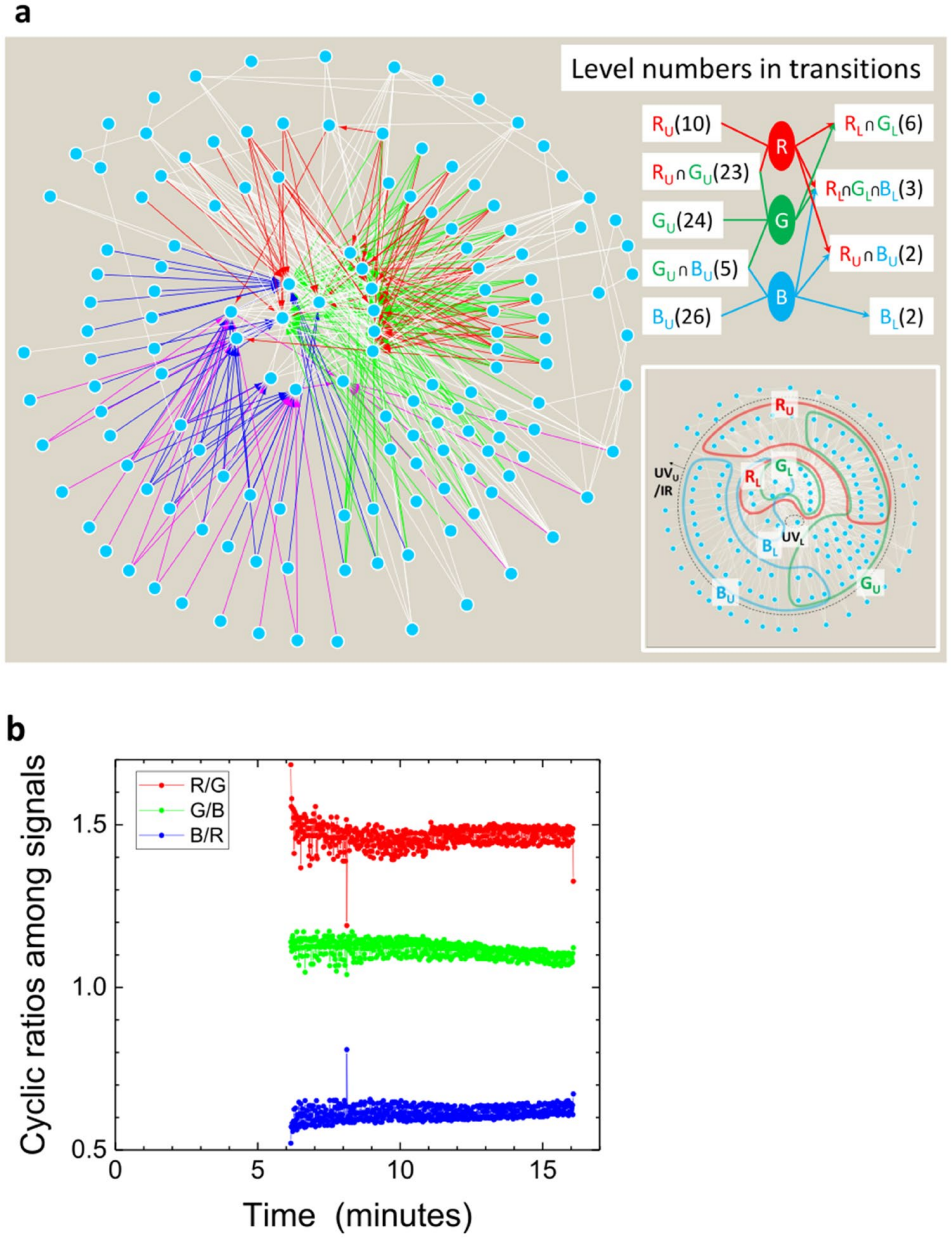
Information on the R, G, and B outputs of the sensor. **(a)** Transitions of Ar I emissions listed in the literature^32^ (see Supplemental File). Nodes represent energy levels, and directed edges indicate transitions using the colour of each output component of the sensor. We assume that wavelengths from 400 to 500 nm, from 500 to 600 nm and from 600 to 700 nm correspond to B, G, and R components, and subscripts U and L indicate upper and lower energy levels in transitions. Node groups and their node numbers are shown on the right side. **(b)** Ratios of R to G, G to B, and B to R of data in Fig. 3a.

One may consider that these R, G, and B signals have wavelength resolutions too broad to determine what happens inside plasma. However, these signals provide significant information on the balance of neutral atoms in the plasma at excited states. Ar I emissions arising from the excited levels of Ar atoms include 428 spectra, all listed in Ref.^32^, expanding from the ultraviolet (UV) to middle infrared ranges. The list of transitions between quantum levels, unique to each atom species, is too complicated for intuitive understanding (see the supplemental file). However, we can obtain clearer insight by using a graphic representation with nodes representing excited levels and edges representing transitions (Fig. 4a), such as the expressions of complex chemical systems in molecular plasmas^33–35^. For instance, spectra in the R component (from approximately 600 to 700 nm) originate primarily from the transitions from the 3*s*^2^3*p*^5^5*d*’ to 3*s*^2^3*p*^5^4*p* group in the Racah notation.

Generally, with some overlapping areas, transitions yielding emissions for the B, G, and R components are divided by area in this graphic representation. Using L and U for lower and upper energy levels for emissive transitions in the subscripts of group names, we obtain an overview of the energy transitions in Ar I emissions. The area for B_U_ is shown in the lower-left part of Fig. 4a, in which nodes representing the upper levels of energy for emissions from 400 to 500 nm are present and superposed with the ultraviolet (UV) region. The area for G_U_ is on the right-hand side and that for R_U_ is shown mostly in the upper part of Fig. 4a. Area B_U_ shares a partial area, corresponding to five nodes, with G_U_. Areas G_U_ and R_U_ share 23 nodes, but areas B_U_ and R_U_ share no nodes. The numbers of the lower levels for the R, G, and B components are much smaller, limited to 12 nodes, and many of these nodes are shared by the B_L_, G_L_, and R_L_ groups. We found three exceptions that cannot be included in the preceding description. One level in the R_U_ group also serves as a lower level for an emission in the R component, and another level in the R_U_ and G_U_ mixed group serves also an upper level for an emission from the R component. The remaining exceptional fact is that we observed one H-atom emission spectrum at 656.2 nm (in Fig. 3b and the Supplemental File), which is probably due to H_2_O impurities and contributes slightly to the R component. However, the main system of optical emissions consists of groups of upper (R_U_, G_U_, and B_U_) and lower (R_L_, G_L_, and B_L_) levels and their crossing directed links.

By detecting ratios among the three components, we can judge which part shown in Fig. 4a is more active during transitions inside plasma. Figure 4b shows the ratios of components R to G, G to B, and B to R detected by the sensor in the low-pressure chamber, as shown in Fig. 3a. For 10 min, the changes in signals were complicated, which may reflect a stabilization stage of the plasma structure. After 10 min, the input power caused the plasma parameters to vary monotonically. The ratio of R to G was constant during this phase; notably, the R and G components share many upper energy levels, making this constant tendency reasonable. The ratio of B to R increases, and that of G to B decreases; these two dependences indicate enhancement of a relative increase of the B component. In fact, as shown in Fig. 3b, the intensities of the transitions from 3*s*^2^3*p*^5^5*p*’ to 3*s*^2^3*p*^5^4*s* at 415.8591, 419.8317, and 420.0675 nm increased correspondingly.

In this experiment, we set the colour sensor to detect plasma emissions while comparable data monitored by a monochromator were available from the same equipment. We successfully confirmed the validity of the operation at low pressure and the data accumulation outside the vacuum chamber and found reasonable agreement of both datasets. At this point, this sensor was ready for use in a larger vacuum chamber with plasmas and/or other light emitting objects, which were unobservable from outside the vacuum chambers. The sensor uses paths through small vacuum windows for data transfer by wireless communication and installing colour sensors allowed us to visualize phenomena at low pressure inside the industrial vacuum chamber systems. According to the most rigorous definition, this sensor is not an edge computing device with local and flexible intelligence^8–12^ but is equivalently capable of in situ computing. Such in situ data processing for accurate data acquisition is a promising trend for scientific and industrial sensor systems that allow us to secure a large amount of reliable data.

## Concluding remarks

In this study, we demonstrated that our in-vacuum active colour sensor successfully detected direct optical lights emitted from discharge plasma under the low-pressure conditions of a vacuum chamber, computed analogue signals to digital numerals, and sent them to a storage device located outside the chamber. The capability of the colour sensor was invariant in cases where the gas pressure of its location, 30 Pa, varied from the atmospheric pressure, and we observed no significant outgases caused by possible detachment from the sensor surface, as long as we investigated them with a typical gas-pressure gauge. Its capable function did not vary even when the plasma was ignited at a position 10 mm away from the sensor, and the sensor successfully detected plasma emission signals as R, G, and B components of the output. This low-cost and easy-to-install sensor can provide useful data on the raw emission signals of the balance between excited energy states of discharge gases in the out-of-reach regions of plasma reactors without any filtering effects.

## Methods

### Design of the in-vacuum active sensor

Our in-vacuum active sensor is composed of printed circuit boards, discrete electronic circuit devices, and a sealing container. The required features in the entire sensing system for our purpose are summarized in three elements: RGB sensitivities were captured by a tiny integrated photodiode module, IoT capability enabled the data transfer from raw RGB digital bit-data through wireless communications, and the active functions of the sensor and computing electronic devices were robust.

It is preferable for the in situ sensor to occupy a small area or volume in the system that the device monitors; therefore, we adopted an RGB colour sensor (S11059-02DT, Hamamatsu Photonics K.K.), which is an integrated photodiode module chip with a sensitive area of 0.54 by 1.1 mm^2^. It accepts several commands including address calls, register calls, and data read-outs, and sends 8-bit outputs for each component (R, G, or B). While several types of colour-matching functions for wavelength spectra have been proposed^36^, we did not use the possible corrections of RGB values as outputs of the sensor and used these raw data to confirm the direct measurements on plasma emission information without any significant noise contamination. Instead of rigorous calibration of these data as R, G, and B values, we assured their linkage to chroma and hue values as described below^26^. We built up a convenient RGB-sensitive colour detector (Color Checker CC-01, Checkers Co.) that included a white light emitting diode (LED) with the same RGB colour sensor (S11059-02DT, Hamamatsu Photonics K.K.). This handy system was used for the calibration of the RGB colour sensor, as described in Ref.^26^ and summarized below. We performed supervised learning for two-layer neural networks using more than 1600 colour catalogue samples (Pantone Formula Guide, Pantone LLC, X-Rite Inc.) as training data. The neural networks identified positions on the CIE 1931 colour *xy* coordinates on which the values of chroma and hue were defined^36^. In addition, we linked the two counter colours, white and black, to the sensor outputs as shown below. We used a colourless, blank part of a white Pantone catalogue sheet and coated it white because white colour can be defined as a state with no absorption of reflected lights; the R, G, and B values in this test were set to zero. The black colour is equivalent to a case with the signal levels when no reflector is installed for the sensor, as all the R, G, and B lights are completely absorbed and their corresponding values in the sensor outputs are set to be the maximum. Although both of these colours correspond to (*x*, *y*) = (0.333, 0.333) on the colour coordinate, the sensor outputs are located at the very ends on both sides. Consequently, based on this assumption, it is possible to use this tiny RGB colour sensor solely in our in-vacuum active device with the working standard calibration level^1^. The colour sensor also has an infrared-ray (IR) signal whose spectrum has a peak wavelength of 855 nm and ranges from 785 to 885 nm, although its value is neither calibrated nor used in our experiment.

After receiving light and converting the light levels into numeral outputs in the photodiode array chip, a RISC micro-controller (AVR Microcontroller MEGA88V, Atmel Corp.) sent the outputs in nine bits via Bluetooth standard wireless communication to a master personal computer that sends data-transfer request messages to the sensor. Additionally, this RISC micro-controller includes the abovementioned calibration procedure for optimized two-layer neural networks. Another chip (ADM3202, Analog Devices Inc.) performed the Bluetooth wireless communication. All the circuit components were in a metallic (aluminium) container (70 × 20 × 83 mm^3^) with an open perforated hole (260 mm^2^) for wireless communication. Bluetooth wireless communication between inside sensors and outside data storage devices such as personal computers may be unstable or impossible if there are not enough paths for microwaves of approximately 2.4 GHz. This difficulty can be overcome, as we recently successfully observed microwave cloaking in plasma space^27,37–39^, by detouring the signals around the metallic components. This technique was not introduced in our system described here, although it is ready for practical use by setting anisotropic metamaterials around the dielectric window that faces both the low-pressure plasma space and the ambient air. The signals transferred to the personal computer are shown in its display and are simultaneously stored in its memory every second in line with the evolutions of R, G, B, and IR signals with time.

A printed circuit board (PCB), which was a typical glass epoxy plate (FR-4, frame retardant type 4), held the discrete devices with circuit connections composed of classical copper lines arranged by computer-aided design. The surface finish was coverage of organic solderability preservative (OSP) on the Cu lines^40^ and thin-film solder mask on the other area; these finish layers prevent copper corrosion and, after a baking process of approximately 120 degrees Celsius, achieve high thermal endurance that may repel the bombardment of plasma particles. The maximum temperature was fixed at a working temperature of 80 degrees Celsius for the RGB colour sensor, which tolerated the installation of the metallic-sealed sensor system on the vacuum chamber wall, which contacts the ambient air at room temperature even when a heater for the substrates, etc., is used somewhere else in the chamber. The electronic circuit’s power supply was directed from outside the chamber through a vacuum-sealed connector; however, battery technology for satellites was sufficient for our purpose^41^. Recently, lithium-ion batteries have also been available for this purpose, since, before commercial use, they have been tested under the UN/DOT 38.3 regulation, which stipulates that endurance at a pressure of 11.6 kPa or less is required for aircraft equipment^42^. The pressure difference of the test and the ambient environment (100 kPa) was approximately 88 kPa, which is only slightly less than that in our case (~ 100 kPa). One electrolyte-filling process method for lithium-ion batteries is performed under low pressure in a vacuum chamber^43,44^, which indicates that inclusion of these batteries in a vacuum chamber is possible.

### Experimental setup of vacuum chamber

**Figure 5:**
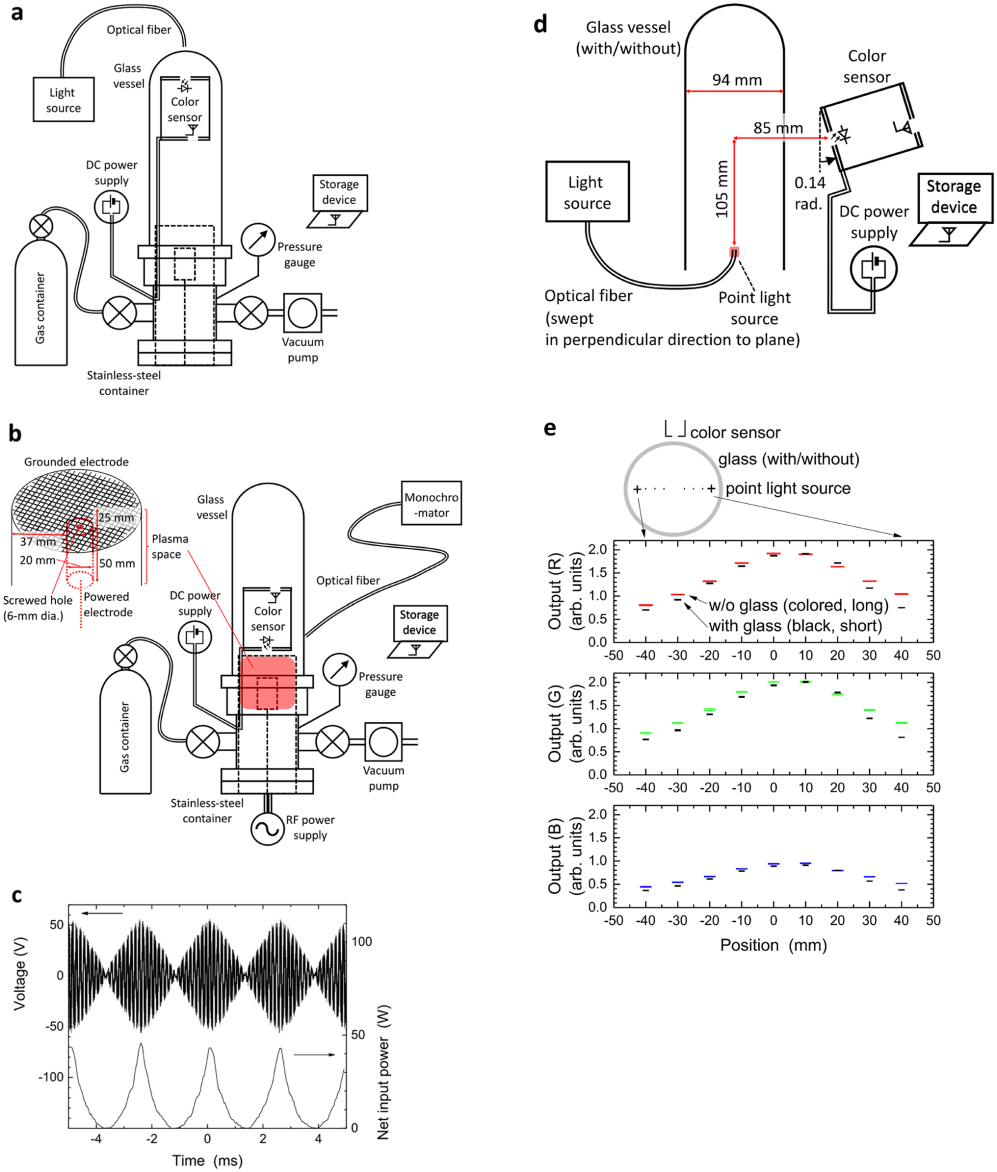
Setups of the vacuum chamber with colour sensor installation. **(a)** Detection of externally-controlled light. The data in Fig. 2 are obtained in this setup. **(b)** Detection of Ar plasma emissions. The data in Fig. 3 are obtained from this setup. **(c)** Example time evolutions of radiofrequency signals: forward voltage monitored by a directional coupler located before the powered electrode (upper side) and net input power (lower side). **(d)** Identification of the difference between direct (without glass) and indirect (with glass) sensing. **(e)** Colour sensor signals in direct and indirect measurements in the setup shown in **(d)**.

We used a compact vacuum chamber made of a stainless steel container and a fused-quartz glass vessel for the experiments, which were the common components previously reported^27^. The inset photograph in Fig. 3a shows a partial view of the chamber and the specific chamber configuration is shown in Fig. 5. The chamber wall of the upper part was fused-quartz glass through which both visible light and microwaves can penetrate it, with an outer diameter of 100 mm at 3-mm thickness. As shown in Fig. 5a, this upper part is useful for detection of the externally-supplied light source. The light from the halogen lamp was injected from the top of the glass vessel and due to optical transparency, the colour sensor detected the light with almost no depression. The glass vessel is also transparent for Bluetooth signals at approximately 2.4 GHz, which allows the microwave to propagate freely between the sensor in the chamber and the storage device outside the chamber. The lower part was enclosed by a stainless-steel wall and works only for adjustment of gas pressure, which depends on the balance between the openings of two valves for the gas-feed inlet and outlet.

The main function of the lower part of the chamber was room for plasma, as shown in Fig. 5b. A cylindrical grounded electrode made of a thin aluminium plate covered its inner surface with a diameter of 94 mm, which was electrically continuous to the metallic mesh electrode on the top side of this cylinder (Fig. 5b). Inside this metallic cylindrical shell, we installed a powered metallic electrode made of stainless steel whose diameter was 20 mm with a 6-mm screw hole. The distance between the powered and the grounded metallic mesh electrodes was 25 mm, whereas that on the side of the powered electrode was 37 mm. This spatial configuration supports two-discharge geometry; one is discharge between the powered and grounded electrodes, and the other is hollow-cathode discharge^45^ mainly generated in the screw hole. As shown in Fig. 3a, both discharge regimes were observed and sufficient emissions of visible light with high signal-to-noise ratios enabled us to confirm the validity of this measurement system. As in the experiments of the externally provided light detection, the sensor was installed in the upper part of the chamber, i.e. in the glass vessel, but its optical inlet was to the lower side to detect plasma emissions. Due to the transparency of the upper part, we can expect smooth wireless communication and monochromator measurements of visible emissions from inside to outside.

The method for supplying power for plasma generation was a classical one: a signal generator (WF1974, NF Corporation) of an RF wave at 13.56 MHz amplified by 55 dB by an RF amplifier (T145-5546A, Thamway Co., Ltd). We then transferred the RF power with several tens of watts into the vacuum chamber through the matching circuit with parallel and series capacitors with a series inductor. The temporal envelope of the power input was in the shape of a triangle at 400 Hz (Fig. 5c). A 40-dB directional coupler (T051-5726A, Thamway Co., Ltd) located between the output of the amplifier and the powered electrode monitored forward and reflected power levels. The gas inlet and outlet valves were on the sides of the chamber on the respective opposite directions, and the Ar gas at 20 mL/min flowed with its pressure set to 100 Pa by adjusting the opening ratio of the outlet valve, which was connected to an oil-sealed rotary-vane vacuum pump. Gas pressure was monitored by a thermocouple vacuum gauge (M-012DM, Canon Anelva Corporation).

Figure 5c shows an example of the RF signals monitored in one parameter (level 15, the maximum in our experiments). The RF forward voltage was detected by the directional coupler. The net input power was calculated from the forward power component from which the reflected power was subtracted. In Fig. 3, we plotted the average values of the net input power.

As aforementioned, the fused-quartz glass vessel was sufficiently transparent to visible light, but it deformed light signals from the inner space (e.g., plasma emission in this study) when we installed our in-vacuum colour sensor outside the vessel as in the conventional manner. To confirm the advantage of inner installation for data acquisition, we performed an experiment of light detection in the setup shown in Fig. 5d at atmospheric pressure and compared the results. In this case, light emission from the optical fibre outlet imitated the local plasma emission from the corresponding spatial point. As we swept the optical fibre, when the point light source at locations near the vessel wall, the profiles of the signals detected outside the vessel were depressed due to nonuniform glass refraction and surface reflection of the light, while those in the centre were unchanged (Fig. 5e). Curvatures in glass or other obstacles, such as contaminated surfaces, are likely to affect the light signals, but no signal change occurred when we set our colour sensor inside the vacuum vessels or chambers. Our concept of colour sensor installation in a vacuum to detect inside light is definitely superior to sensors sitting outside a vacuum container because, in the vacuum, no absorber, reflector, or refractive medium exists between the light source and the colour sensor.

## Supplementary Information

Supplementary Information.
