# Supplementary material for: In-vacuum active colour sensor and wireless communication across a vacuum-air interface
Source: Sci Rep. 2021 Jan 14;11:1364. doi: 10.1038/s41598-020-80501-z (PMC7809036; doi:10.1038/s41598-020-80501-z)
Supplement: Supplementary file 1 — Supplementary Information. [file 41598_2020_80501_MOESM1_ESM.docx]

Supplementary Information

**Title:** In-vacuum active colour sensor and wireless communication across a vacuum-air interface

**Authors:** Osamu Sakai, Takayuki Kitagawa, Keiji Sakurai, Go Itami, Shigeyuki Miyagi, Kazuyuki Noborio & Kohshi Taguchi

A. Supplementary information for Ar I emissions

In an Ar glow-discharge plasma generated in this study, most of the spectra contributing to components detected by the optical sensor and observed in the monochromator belong to the Ar I system, which are transitions between two electron-excited states of neutral Ar atoms. In Ref. [32], information on all the lines in Ar I is available, and we used it as listed in Tab. A1 and shown in Fig. A1. Here, notation in the two columns of “Symbol name” shows node names used in Fig. A2, which are the enlarged views of Fig. 4(a), and abbreviations in the column “Group” indicate tentative correspondences to R, G and B outputs of the sensor, where, below B, the wavelength range of ultraviolet light exists which we call “UV” whereas we call “IR” for infrared light with longer wavelength than those in the R region.

Table A1. Ar I lines stored as database in Ref. [32]. Angular brackets in configuration columns enclose *J* values of parent term. Superscript “^o^” in term columns indicate odd parity. *J*-values in *J* columns represent total electronic angular momentum.

| Upper level | | | | Lower level | | | | Wavelength | |
| --- | --- | --- | --- | --- | --- | --- | --- | --- | --- |
| Configuration | Term | *J* | Symbol name | Configuration | Term | *J* | Symbol name | (nm) | Group |
| 3*s*^2^3*p*^5^(^2^P°_1/2_)3*d* | ^2^[^3^/_2_]° | 1 | 1/2)3d2[3/2]o1 | 3*s*^2^3*p*^6^ | ^1^S | 0 | 1S0 | 86.6800 | UV |
| 3*s*^2^3*p*^5^(^2^P°_1/2_)5*s* | ^2^[^1^/_2_]° | 1 | 1/2)5s2[1/2]o1 | 3*s*^2^3*p*^6^ | ^1^S | 0 | 1S0 | 86.9754 | UV |
| 3*s*^2^3*p*^5^(^2^P°_3/2_)3*d* | ^2^[^3^/_2_]° | 1 | 3/2)3d2[3/2]o1 | 3*s*^2^3*p*^6^ | ^1^S | 0 | 1S0 | 87.6058 | UV |
| 3*s*^2^3*p*^5^(^2^P°_3/2_)5*s* | ^2^[^3^/_2_]° | 1 | 3/2)5s2[3/2]o1 | 3*s*^2^3*p*^6^ | ^1^S | 0 | 1S0 | 87.9947 | UV |
| 3*s*^2^3*p*^5^(^2^P°_3/2_)3*d* | ^2^[^1^/_2_]° | 1 | 3/2)3d2[1/2]o1 | 3*s*^2^3*p*^6^ | ^1^S | 0 | 1S0 | 89.4310 | UV |
| 3*s*^2^3*p*^5^(^2^P°_1/2_)4*s* | ^2^[^1^/_2_]° | 1 | 1/2)4s2[1/2]o1 | 3*s*^2^3*p*^6^ | ^1^S | 0 | 1S0 | 104.8220 | UV |
| 3*s*^2^3*p*^5^(^2^P°_3/2_)4*s* | ^2^[^3^/_2_]° | 1 | 3/2)4s2[3/2]o1 | 3*s*^2^3*p*^6^ | ^1^S | 0 | 1S0 | 106.6660 | UV |
| 3*s*^2^3*p*^5^(^2^P°_1/2_)7*p* | ^2^[^1^/_2_] | 0 | 1/2)7p2[1/2]0 | 3*s*^2^3*p*^5^(^2^P°_1/2_)4*s* | ^2^[^1^/_2_]° | 1 | 1/2)4s2[1/2]o1 | 340.6180 | UV |
| 3*s*^2^3*p*^5^(^2^P°_1/2_)6*p* | ^2^[^3^/_2_] | 2 | 1/2)6p2[3/2]2 | 3*s*^2^3*p*^5^(^2^P°_3/2_)4*s* | ^2^[^3^/_2_]° | 1 | 3/2)4s2[3/2]o1 | 346.1078 | UV |
| 3*s*^2^3*p*^5^(^2^P°_3/2_)6*p* | ^2^[^3^/_2_] | 2 | 3/2)6p2[3/2]2 | 3*s*^2^3*p*^5^(^2^P°_3/2_)4*s* | ^2^[^3^/_2_]° | 2 | 3/2)4s2[3/2]o2 | 355.4305 | UV |
| 3*s*^2^3*p*^5^(^2^P°_1/2_)6*p* | ^2^[^3^/_2_] | 1 | 1/2)6p2[3/2]1 | 3*s*^2^3*p*^5^(^2^P°_1/2_)4*s* | ^2^[^1^/_2_]° | 0 | 1/2)4s2[1/2]o0 | 356.3286 | UV |
| 3*s*^2^3*p*^5^(^2^P°_3/2_)6*p* | ^2^[^5^/_2_] | 3 | 3/2)6p2[5/2]3 | 3*s*^2^3*p*^5^(^2^P°_3/2_)4*s* | ^2^[^3^/_2_]° | 2 | 3/2)4s2[3/2]o2 | 356.7656 | UV |
| 3*s*^2^3*p*^5^(^2^P°_3/2_)7*p* | ^2^[^1^/_2_] | 0 | 3/2)7p2[1/2]0 | 3*s*^2^3*p*^5^(^2^P°_1/2_)4*s* | ^2^[^1^/_2_]° | 1 | 1/2)4s2[1/2]o1 | 357.2295 | UV |
| 3*s*^2^3*p*^5^(^2^P°_3/2_)6*p* | ^2^[^1^/_2_] | 0 | 3/2)6p2[1/2]0 | 3*s*^2^3*p*^5^(^2^P°_3/2_)4*s* | ^2^[^3^/_2_]° | 1 | 3/2)4s2[3/2]o1 | 360.6522 | UV |
| 3*s*^2^3*p*^5^(^2^P°_3/2_)6*p* | ^2^[^3^/_2_] | 2 | 3/2)6p2[3/2]2 | 3*s*^2^3*p*^5^(^2^P°_3/2_)4*s* | ^2^[^3^/_2_]° | 1 | 3/2)4s2[3/2]o1 | 363.2683 | UV |
| 3*s*^2^3*p*^5^(^2^P°_3/2_)6*p* | ^2^[^3^/_2_] | 1 | 3/2)6p2[3/2]1 | 3*s*^2^3*p*^5^(^2^P°_3/2_)4*s* | ^2^[^3^/_2_]° | 1 | 3/2)4s2[3/2]o1 | 363.4460 | UV |
| 3*s*^2^3*p*^5^(^2^P°_3/2_)6*p* | ^2^[^5^/_2_] | 2 | 3/2)6p2[5/2]2 | 3*s*^2^3*p*^5^(^2^P°_3/2_)4*s* | ^2^[^3^/_2_]° | 1 | 3/2)4s2[3/2]o1 | 364.3116 | UV |
| 3*s*^2^3*p*^5^(^2^P°_1/2_)6*p* | ^2^[^1^/_2_] | 0 | 1/2)6p2[1/2]0 | 3*s*^2^3*p*^5^(^2^P°_1/2_)4*s* | ^2^[^1^/_2_]° | 1 | 1/2)4s2[1/2]o1 | 364.9832 | UV |
| 3*s*^2^3*p*^5^(^2^P°_3/2_)6*p* | ^2^[^1^/_2_] | 1 | 3/2)6p2[1/2]1 | 3*s*^2^3*p*^5^(^2^P°_3/2_)4*s* | ^2^[^3^/_2_]° | 1 | 3/2)4s2[3/2]o1 | 365.9529 | UV |
| 3*s*^2^3*p*^5^(^2^P°_1/2_)6*p* | ^2^[^3^/_2_] | 2 | 1/2)6p2[3/2]2 | 3*s*^2^3*p*^5^(^2^P°_1/2_)4*s* | ^2^[^1^/_2_]° | 1 | 1/2)4s2[1/2]o1 | 367.0670 | UV |
| 3*s*^2^3*p*^5^(^2^P°_1/2_)6*p* | ^2^[^1^/_2_] | 1 | 1/2)6p2[1/2]1 | 3*s*^2^3*p*^5^(^2^P°_1/2_)4*s* | ^2^[^1^/_2_]° | 1 | 1/2)4s2[1/2]o1 | 367.5235 | UV |
| 3*s*^2^3*p*^5^(^2^P°_3/2_)6*p* | ^2^[^1^/_2_] | 1 | 3/2)6p2[1/2]1 | 3*s*^2^3*p*^5^(^2^P°_1/2_)4*s* | ^2^[^1^/_2_]° | 0 | 1/2)4s2[1/2]o0 | 377.0369 | UV |
| 3*s*^2^3*p*^5^(^2^P°_3/2_)6*p* | ^2^[^1^/_2_] | 0 | 3/2)6p2[1/2]0 | 3*s*^2^3*p*^5^(^2^P°_1/2_)4*s* | ^2^[^1^/_2_]° | 1 | 1/2)4s2[1/2]o1 | 383.4678 | UV |
| 3*s*^2^3*p*^5^(^2^P°_3/2_)6*p* | ^2^[^1^/_2_] | 1 | 3/2)6p2[1/2]1 | 3*s*^2^3*p*^5^(^2^P°_1/2_)4*s* | ^2^[^1^/_2_]° | 1 | 1/2)4s2[1/2]o1 | 389.4660 | UV |
| 3*s*^2^3*p*^5^(^2^P°_1/2_)5*p* | ^2^[^3^/_2_] | 2 | 1/2)5p2[3/2]2 | 3*s*^2^3*p*^5^(^2^P°_3/2_)4*s* | ^2^[^3^/_2_]° | 2 | 3/2)4s2[3/2]o2 | 394.7505 | UV |
| 3*s*^2^3*p*^5^(^2^P°_1/2_)5*p* | ^2^[^1^/_2_] | 1 | 1/2)5p2[1/2]1 | 3*s*^2^3*p*^5^(^2^P°_3/2_)4*s* | ^2^[^3^/_2_]° | 2 | 3/2)4s2[3/2]o2 | 394.8979 | UV |
| 3*s*^2^3*p*^5^(^2^P°_1/2_)5*p* | ^2^[^3^/_2_] | 2 | 1/2)5p2[3/2]2 | 3*s*^2^3*p*^5^(^2^P°_3/2_)4*s* | ^2^[^3^/_2_]° | 1 | 3/2)4s2[3/2]o1 | 404.4418 | B |
| 3*s*^2^3*p*^5^(^2^P°_1/2_)5*p* | ^2^[^1^/_2_] | 1 | 1/2)5p2[1/2]1 | 3*s*^2^3*p*^5^(^2^P°_3/2_)4*s* | ^2^[^3^/_2_]° | 1 | 3/2)4s2[3/2]o1 | 404.5965 | B |
| 3*s*^2^3*p*^5^(^2^P°_1/2_)5*p* | ^2^[^3^/_2_] | 1 | 1/2)5p2[3/2]1 | 3*s*^2^3*p*^5^(^2^P°_3/2_)4*s* | ^2^[^3^/_2_]° | 1 | 3/2)4s2[3/2]o1 | 405.4526 | B |
| 3*s*^2^3*p*^5^(^2^P°_3/2_)5*p* | ^2^[^3^/_2_] | 2 | 3/2)5p2[3/2]2 | 3*s*^2^3*p*^5^(^2^P°_3/2_)4*s* | ^2^[^3^/_2_]° | 2 | 3/2)4s2[3/2]o2 | 415.8591 | B |
| 3*s*^2^3*p*^5^(^2^P°_3/2_)5*p* | ^2^[^3^/_2_] | 1 | 3/2)5p2[3/2]1 | 3*s*^2^3*p*^5^(^2^P°_3/2_)4*s* | ^2^[^3^/_2_]° | 2 | 3/2)4s2[3/2]o2 | 416.4180 | B |
| 3*s*^2^3*p*^5^(^2^P°_1/2_)5*p* | ^2^[^1^/_2_] | 1 | 1/2)5p2[1/2]1 | 3*s*^2^3*p*^5^(^2^P°_1/2_)4*s* | ^2^[^1^/_2_]° | 0 | 1/2)4s2[1/2]o0 | 418.1884 | B |
| 3*s*^2^3*p*^5^(^2^P°_3/2_)5*p* | ^2^[^5^/_2_] | 2 | 3/2)5p2[5/2]2 | 3*s*^2^3*p*^5^(^2^P°_3/2_)4*s* | ^2^[^3^/_2_]° | 2 | 3/2)4s2[3/2]o2 | 419.0713 | B |
| 3*s*^2^3*p*^5^(^2^P°_1/2_)5*p* | ^2^[^3^/_2_] | 1 | 1/2)5p2[3/2]1 | 3*s*^2^3*p*^5^(^2^P°_1/2_)4*s* | ^2^[^1^/_2_]° | 0 | 1/2)4s2[1/2]o0 | 419.1029 | B |
| 3*s*^2^3*p*^5^(^2^P°_3/2_)5*p* | ^2^[^1^/_2_] | 0 | 3/2)5p2[1/2]0 | 3*s*^2^3*p*^5^(^2^P°_3/2_)4*s* | ^2^[^3^/_2_]° | 1 | 3/2)4s2[3/2]o1 | 419.8317 | B |
| 3*s*^2^3*p*^5^(^2^P°_3/2_)5*p* | ^2^[^5^/_2_] | 3 | 3/2)5p2[5/2]3 | 3*s*^2^3*p*^5^(^2^P°_3/2_)4*s* | ^2^[^3^/_2_]° | 2 | 3/2)4s2[3/2]o2 | 420.0675 | B |
| 3*s*^2^3*p*^5^(^2^P°_3/2_)5*p* | ^2^[^1^/_2_] | 1 | 3/2)5p2[1/2]1 | 3*s*^2^3*p*^5^(^2^P°_3/2_)4*s* | ^2^[^3^/_2_]° | 2 | 3/2)4s2[3/2]o2 | 425.1185 | B |
| 3*s*^2^3*p*^5^(^2^P°_1/2_)5*p* | ^2^[^1^/_2_] | 0 | 1/2)5p2[1/2]0 | 3*s*^2^3*p*^5^(^2^P°_1/2_)4*s* | ^2^[^1^/_2_]° | 1 | 1/2)4s2[1/2]o1 | 425.9362 | B |
| 3*s*^2^3*p*^5^(^2^P°_3/2_)5*p* | ^2^[^3^/_2_] | 2 | 3/2)5p2[3/2]2 | 3*s*^2^3*p*^5^(^2^P°_3/2_)4*s* | ^2^[^3^/_2_]° | 1 | 3/2)4s2[3/2]o1 | 426.6287 | B |
| 3*s*^2^3*p*^5^(^2^P°_3/2_)5*p* | ^2^[^3^/_2_] | 1 | 3/2)5p2[3/2]1 | 3*s*^2^3*p*^5^(^2^P°_3/2_)4*s* | ^2^[^3^/_2_]° | 1 | 3/2)4s2[3/2]o1 | 427.2169 | B |
| 3*s*^2^3*p*^5^(^2^P°_3/2_)5*p* | ^2^[^5^/_2_] | 2 | 3/2)5p2[5/2]2 | 3*s*^2^3*p*^5^(^2^P°_3/2_)4*s* | ^2^[^3^/_2_]° | 1 | 3/2)4s2[3/2]o1 | 430.0101 | B |
| 3*s*^2^3*p*^5^(^2^P°_1/2_)5*p* | ^2^[^3^/_2_] | 2 | 1/2)5p2[3/2]2 | 3*s*^2^3*p*^5^(^2^P°_1/2_)4*s* | ^2^[^1^/_2_]° | 1 | 1/2)4s2[1/2]o1 | 433.3561 | B |
| 3*s*^2^3*p*^5^(^2^P°_1/2_)5*p* | ^2^[^1^/_2_] | 1 | 1/2)5p2[1/2]1 | 3*s*^2^3*p*^5^(^2^P°_1/2_)4*s* | ^2^[^1^/_2_]° | 1 | 1/2)4s2[1/2]o1 | 433.5338 | B |
| 3*s*^2^3*p*^5^(^2^P°_1/2_)5*p* | ^2^[^3^/_2_] | 1 | 1/2)5p2[3/2]1 | 3*s*^2^3*p*^5^(^2^P°_1/2_)4*s* | ^2^[^1^/_2_]° | 1 | 1/2)4s2[1/2]o1 | 434.5168 | B |
| 3*s*^2^3*p*^5^(^2^P°_3/2_)5*p* | ^2^[^1^/_2_] | 1 | 3/2)5p2[1/2]1 | 3*s*^2^3*p*^5^(^2^P°_3/2_)4*s* | ^2^[^3^/_2_]° | 1 | 3/2)4s2[3/2]o1 | 436.3795 | B |
| 3*s*^2^3*p*^5^(^2^P°_3/2_)5*p* | ^2^[^3^/_2_] | 1 | 3/2)5p2[3/2]1 | 3*s*^2^3*p*^5^(^2^P°_1/2_)4*s* | ^2^[^1^/_2_]° | 0 | 1/2)4s2[1/2]o0 | 442.3994 | B |
| 3*s*^2^3*p*^5^(^2^P°_3/2_)5*p* | ^2^[^1^/_2_] | 0 | 3/2)5p2[1/2]0 | 3*s*^2^3*p*^5^(^2^P°_1/2_)4*s* | ^2^[^1^/_2_]° | 1 | 1/2)4s2[1/2]o1 | 451.0733 | B |
| 3*s*^2^3*p*^5^(^2^P°_3/2_)5*p* | ^2^[^1^/_2_] | 1 | 3/2)5p2[1/2]1 | 3*s*^2^3*p*^5^(^2^P°_1/2_)4*s* | ^2^[^1^/_2_]° | 0 | 1/2)4s2[1/2]o0 | 452.2323 | B |
| 3*s*^2^3*p*^5^(^2^P°_3/2_)11*d* | ^2^[^1^/_2_]° | 1 | 3/2)11d2[1/2]o1 | 3*s*^2^3*p*^5^(^2^P°_3/2_)4*p* | ^2^[^1^/_2_] | 1 | 3/2)4p2[1/2]1 | 454.4746 | B |
| 3*s*^2^3*p*^5^(^2^P°_1/2_)7*d* | ^2^[^3^/_2_]° | 2 | 1/2)7d2[3/2]o2 | 3*s*^2^3*p*^5^(^2^P°_3/2_)4*p* | ^2^[^1^/_2_] | 1 | 3/2)4p2[1/2]1 | 455.4324 | B |
| 3*s*^2^3*p*^5^(^2^P°_3/2_)10*d* | ^2^[^3^/_2_]° | 2 | 3/2)10d2[3/2]o2 | 3*s*^2^3*p*^5^(^2^P°_3/2_)4*p* | ^2^[^1^/_2_] | 1 | 3/2)4p2[1/2]1 | 458.4956 | B |
| 3*s*^2^3*p*^5^(^2^P°_3/2_)10*d* | ^2^[^1^/_2_]° | 1 | 3/2)10d2[1/2]o1 | 3*s*^2^3*p*^5^(^2^P°_3/2_)4*p* | ^2^[^1^/_2_] | 1 | 3/2)4p2[1/2]1 | 458.6611 | B |
| 3*s*^2^3*p*^5^(^2^P°_3/2_)10*d* | ^2^[^1^/_2_]° | 0 | 3/2)10d2[1/2]o0 | 3*s*^2^3*p*^5^(^2^P°_3/2_)4*p* | ^2^[^1^/_2_] | 1 | 3/2)4p2[1/2]1 | 458.7209 | B |
| 3*s*^2^3*p*^5^(^2^P°_3/2_)5*p* | ^2^[^3^/_2_] | 2 | 3/2)5p2[3/2]2 | 3*s*^2^3*p*^5^(^2^P°_1/2_)4*s* | ^2^[^1^/_2_]° | 1 | 1/2)4s2[1/2]o1 | 458.9289 | B |
| 3*s*^2^3*p*^5^(^2^P°_3/2_)5*p* | ^2^[^3^/_2_] | 1 | 3/2)5p2[3/2]1 | 3*s*^2^3*p*^5^(^2^P°_1/2_)4*s* | ^2^[^1^/_2_]° | 1 | 1/2)4s2[1/2]o1 | 459.6097 | B |
| 3*s*^2^3*p*^5^(^2^P°_3/2_)5*p* | ^2^[^5^/_2_] | 2 | 3/2)5p2[5/2]2 | 3*s*^2^3*p*^5^(^2^P°_1/2_)4*s* | ^2^[^1^/_2_]° | 1 | 1/2)4s2[1/2]o1 | 462.8441 | B |
| 3*s*^2^3*p*^5^(^2^P°_3/2_)9*d* | ^2^[^3^/_2_]° | 2 | 3/2)9d2[3/2]o2 | 3*s*^2^3*p*^5^(^2^P°_3/2_)4*p* | ^2^[^1^/_2_] | 1 | 3/2)4p2[1/2]1 | 464.2137 | B |
| 3*s*^2^3*p*^5^(^2^P°_3/2_)9*d* | ^2^[^1^/_2_]° | 1 | 3/2)9d2[1/2]o1 | 3*s*^2^3*p*^5^(^2^P°_3/2_)4*p* | ^2^[^1^/_2_] | 1 | 3/2)4p2[1/2]1 | 464.7489 | B |
| 3*s*^2^3*p*^5^(^2^P°_3/2_)5*p* | ^2^[^1^/_2_] | 1 | 3/2)5p2[1/2]1 | 3*s*^2^3*p*^5^(^2^P°_1/2_)4*s* | ^2^[^1^/_2_]° | 1 | 1/2)4s2[1/2]o1 | 470.2316 | B |
| 3*s*^2^3*p*^5^(^2^P°_3/2_)8*d* | ^2^[^1^/_2_]° | 0 | 3/2)8d2[1/2]o0 | 3*s*^2^3*p*^5^(^2^P°_3/2_)4*p* | ^2^[^1^/_2_] | 1 | 3/2)4p2[1/2]1 | 474.6821 | B |
| 3*s*^2^3*p*^5^(^2^P°_3/2_)8*d* | ^2^[^1^/_2_]° | 1 | 3/2)8d2[1/2]o1 | 3*s*^2^3*p*^5^(^2^P°_3/2_)4*p* | ^2^[^1^/_2_] | 1 | 3/2)4p2[1/2]1 | 475.2939 | B |
| 3*s*^2^3*p*^5^(^2^P°_1/2_)6*d* | ^2^[^3^/_2_]° | 2 | 1/2)6d2[3/2]o2 | 3*s*^2^3*p*^5^(^2^P°_3/2_)4*p* | ^2^[^1^/_2_] | 1 | 3/2)4p2[1/2]1 | 476.8673 | B |
| 3*s*^2^3*p*^5^(^2^P°_3/2_)12*d* | ^2^[^7^/_2_]° | 4 | 3/2)12d2[7/2]o4 | 3*s*^2^3*p*^5^(^2^P°_3/2_)4*p* | ^2^[^5^/_2_] | 3 | 3/2)4p2[5/2]3 | 479.8742 | B |
| 3*s*^2^3*p*^5^(^2^P°_3/2_)11*d* | ^2^[^7^/_2_]° | 4 | 3/2)11d2[7/2]o4 | 3*s*^2^3*p*^5^(^2^P°_3/2_)4*p* | ^2^[^5^/_2_] | 3 | 3/2)4p2[5/2]3 | 483.5970 | B |
| 3*s*^2^3*p*^5^(^2^P°_3/2_)9*s* | ^2^[^3^/_2_]° | 2 | 3/2)9s2[3/2]o2 | 3*s*^2^3*p*^5^(^2^P°_3/2_)4*p* | ^2^[^1^/_2_] | 1 | 3/2)4p2[1/2]1 | 483.6697 | B |
| 3*s*^2^3*p*^5^(^2^P°_3/2_)7*d* | ^2^[^3^/_2_]° | 2 | 3/2)7d2[3/2]o2 | 3*s*^2^3*p*^5^(^2^P°_3/2_)4*p* | ^2^[^1^/_2_] | 1 | 3/2)4p2[1/2]1 | 487.6261 | B |
| 3*s*^2^3*p*^5^(^2^P°_3/2_)10*d* | ^2^[^7^/_2_]° | 4 | 3/2)10d2[7/2]o4 | 3*s*^2^3*p*^5^(^2^P°_3/2_)4*p* | ^2^[^5^/_2_] | 3 | 3/2)4p2[5/2]3 | 488.6290 | B |
| 3*s*^2^3*p*^5^(^2^P°_3/2_)7*d* | ^2^[^1^/_2_]° | 1 | 3/2)7d2[1/2]o1 | 3*s*^2^3*p*^5^(^2^P°_3/2_)4*p* | ^2^[^1^/_2_] | 1 | 3/2)4p2[1/2]1 | 488.7947 | B |
| 3*s*^2^3*p*^5^(^2^P°_3/2_)7*d* | ^2^[^1^/_2_]° | 0 | 3/2)7d2[1/2]o0 | 3*s*^2^3*p*^5^(^2^P°_3/2_)4*p* | ^2^[^1^/_2_] | 1 | 3/2)4p2[1/2]1 | 489.4691 | B |
| 3*s*^2^3*p*^5^(^2^P°_3/2_)10*d* | ^2^[^7^/_2_]° | 3 | 3/2)10d2[7/2]o3 | 3*s*^2^3*p*^5^(^2^P°_3/2_)4*p* | ^2^[^5^/_2_] | 2 | 3/2)4p2[5/2]2 | 492.1034 | B |
| 3*s*^2^3*p*^5^(^2^P°_3/2_)11*s* | ^2^[^3^/_2_]° | 2 | 3/2)11s2[3/2]o2 | 3*s*^2^3*p*^5^(^2^P°_3/2_)4*p* | ^2^[^5^/_2_] | 3 | 3/2)4p2[5/2]3 | 493.7720 | B |
| 3*s*^2^3*p*^5^(^2^P°_3/2_)9*d* | ^2^[^7^/_2_]° | 4 | 3/2)9d2[7/2]o4 | 3*s*^2^3*p*^5^(^2^P°_3/2_)4*p* | ^2^[^5^/_2_] | 3 | 3/2)4p2[5/2]3 | 495.6750 | B |
| 3*s*^2^3*p*^5^(^2^P°_3/2_)9*d* | ^2^[^7^/_2_]° | 3 | 3/2)9d2[7/2]o3 | 3*s*^2^3*p*^5^(^2^P°_3/2_)4*p* | ^2^[^5^/_2_] | 2 | 3/2)4p2[5/2]2 | 498.9943 | B |
| 3*s*^2^3*p*^5^(^2^P°_3/2_)10*s* | ^2^[^3^/_2_]° | 2 | 3/2)10s2[3/2]o2 | 3*s*^2^3*p*^5^(^2^P°_3/2_)4*p* | ^2^[^5^/_2_] | 3 | 3/2)4p2[5/2]3 | 503.2024 | G |
| 3*s*^2^3*p*^5^(^2^P°_3/2_)8*s* | ^2^[^3^/_2_]° | 2 | 3/2)8s2[3/2]o2 | 3*s*^2^3*p*^5^(^2^P°_3/2_)4*p* | ^2^[^1^/_2_] | 1 | 3/2)4p2[1/2]1 | 504.8811 | G |
| 3*s*^2^3*p*^5^(^2^P°_1/2_)7*s* | ^2^[^1^/_2_]° | 1 | 1/2)7s2[1/2]o1 | 3*s*^2^3*p*^5^(^2^P°_3/2_)4*p* | ^2^[^1^/_2_] | 1 | 3/2)4p2[1/2]1 | 505.4176 | G |
| 3*s*^2^3*p*^5^(^2^P°_1/2_)7*s* | ^2^[^1^/_2_]° | 0 | 1/2)7s2[1/2]o0 | 3*s*^2^3*p*^5^(^2^P°_3/2_)4*p* | ^2^[^1^/_2_] | 1 | 3/2)4p2[1/2]1 | 505.6529 | G |
| 3*s*^2^3*p*^5^(^2^P°_3/2_)8*d* | ^2^[^7^/_2_]° | 4 | 3/2)8d2[7/2]o4 | 3*s*^2^3*p*^5^(^2^P°_3/2_)4*p* | ^2^[^5^/_2_] | 3 | 3/2)4p2[5/2]3 | 506.0079 | G |
| 3*s*^2^3*p*^5^(^2^P°_3/2_)10*s* | ^2^[^3^/_2_]° | 1 | 3/2)10s2[3/2]o1 | 3*s*^2^3*p*^5^(^2^P°_3/2_)4*p* | ^2^[^5^/_2_] | 2 | 3/2)4p2[5/2]2 | 507.0977 | G |
| 3*s*^2^3*p*^5^(^2^P°_3/2_)6*d* | ^2^[^3^/_2_]° | 2 | 3/2)6d2[3/2]o2 | 3*s*^2^3*p*^5^(^2^P°_3/2_)4*p* | ^2^[^1^/_2_] | 1 | 3/2)4p2[1/2]1 | 507.3076 | G |
| 3*s*^2^3*p*^5^(^2^P°_1/2_)6*d* | ^2^[^5^/_2_]° | 3 | 1/2)6d2[5/2]o3 | 3*s*^2^3*p*^5^(^2^P°_3/2_)4*p* | ^2^[^5^/_2_] | 3 | 3/2)4p2[5/2]3 | 507.8033 | G |
| 3*s*^2^3*p*^5^(^2^P°_3/2_)8*d* | ^2^[^7^/_2_]° | 3 | 3/2)8d2[7/2]o3 | 3*s*^2^3*p*^5^(^2^P°_3/2_)4*p* | ^2^[^5^/_2_] | 2 | 3/2)4p2[5/2]2 | 508.7084 | G |
| 3*s*^2^3*p*^5^(^2^P°_3/2_)9*d* | ^2^[^5^/_2_]° | 2 | 3/2)9d2[5/2]o2 | 3*s*^2^3*p*^5^(^2^P°_3/2_)4*p* | ^2^[^3^/_2_] | 1 | 3/2)4p2[3/2]1 | 510.4729 | G |
| 3*s*^2^3*p*^5^(^2^P°_1/2_)6*d* | ^2^[^5^/_2_]° | 3 | 1/2)6d2[5/2]o3 | 3*s*^2^3*p*^5^(^2^P°_3/2_)4*p* | ^2^[^5^/_2_] | 2 | 3/2)4p2[5/2]2 | 511.8202 | G |
| 3*s*^2^3*p*^5^(^2^P°_1/2_)6*d* | ^2^[^5^/_2_]° | 2 | 1/2)6d2[5/2]o2 | 3*s*^2^3*p*^5^(^2^P°_3/2_)4*p* | ^2^[^5^/_2_] | 2 | 3/2)4p2[5/2]2 | 512.7801 | G |
| 3*s*^2^3*p*^5^(^2^P°_3/2_)6*d* | ^2^[^1^/_2_]° | 0 | 3/2)6d2[1/2]o0 | 3*s*^2^3*p*^5^(^2^P°_3/2_)4*p* | ^2^[^1^/_2_] | 1 | 3/2)4p2[1/2]1 | 515.1391 | G |
| 3*s*^2^3*p*^5^(^2^P°_1/2_)5*d* | ^2^[^5^/_2_]° | 2 | 1/2)5d2[5/2]o2 | 3*s*^2^3*p*^5^(^2^P°_3/2_)4*p* | ^2^[^1^/_2_] | 1 | 3/2)4p2[1/2]1 | 515.2303 | G |
| 3*s*^2^3*p*^5^(^2^P°_3/2_)6*d* | ^2^[^1^/_2_]° | 1 | 3/2)6d2[1/2]o1 | 3*s*^2^3*p*^5^(^2^P°_3/2_)4*p* | ^2^[^1^/_2_] | 1 | 3/2)4p2[1/2]1 | 516.2285 | G |
| 3*s*^2^3*p*^5^(^2^P°_3/2_)9*s* | ^2^[^3^/_2_]° | 2 | 3/2)9s2[3/2]o2 | 3*s*^2^3*p*^5^(^2^P°_3/2_)4*p* | ^2^[^5^/_2_] | 3 | 3/2)4p2[5/2]3 | 517.7536 | G |
| 3*s*^2^3*p*^5^(^2^P°_1/2_)5*d* | ^2^[^3^/_2_]° | 2 | 1/2)5d2[3/2]o2 | 3*s*^2^3*p*^5^(^2^P°_3/2_)4*p* | ^2^[^1^/_2_] | 1 | 3/2)4p2[1/2]1 | 518.7746 | G |
| 3*s*^2^3*p*^5^(^2^P°_3/2_)7*d* | ^2^[^5^/_2_]° | 3 | 3/2)7d2[5/2]o3 | 3*s*^2^3*p*^5^(^2^P°_3/2_)4*p* | ^2^[^5^/_2_] | 3 | 3/2)4p2[5/2]3 | 519.2733 | G |
| 3*s*^2^3*p*^5^(^2^P°_1/2_)8*s* | ^2^[^1^/_2_]° | 0 | 1/2)8s2[1/2]o0 | 3*s*^2^3*p*^5^(^2^P°_3/2_)4*p* | ^2^[^3^/_2_] | 1 | 3/2)4p2[3/2]1 | 519.4052 | G |
| 3*s*^2^3*p*^5^(^2^P°_3/2_)7*d* | ^2^[^7^/_2_]° | 3 | 3/2)7d2[7/2]o3 | 3*s*^2^3*p*^5^(^2^P°_3/2_)4*p* | ^2^[^5^/_2_] | 3 | 3/2)4p2[5/2]3 | 521.0486 | G |
| 3*s*^2^3*p*^5^(^2^P°_3/2_)7*d* | ^2^[^3^/_2_]° | 1 | 3/2)7d2[3/2]o1 | 3*s*^2^3*p*^5^(^2^P°_3/2_)4*p* | ^2^[^5^/_2_] | 2 | 3/2)4p2[5/2]2 | 521.4774 | G |
| 3*s*^2^3*p*^5^(^2^P°_3/2_)9*s* | ^2^[^3^/_2_]° | 1 | 3/2)9s2[3/2]o1 | 3*s*^2^3*p*^5^(^2^P°_3/2_)4*p* | ^2^[^5^/_2_] | 2 | 3/2)4p2[5/2]2 | 521.6300 | G |
| 3*s*^2^3*p*^5^(^2^P°_3/2_)7*d* | ^2^[^7^/_2_]° | 4 | 3/2)7d2[7/2]o4 | 3*s*^2^3*p*^5^(^2^P°_3/2_)4*p* | ^2^[^5^/_2_] | 3 | 3/2)4p2[5/2]3 | 522.1270 | G |
| 3*s*^2^3*p*^5^(^2^P°_3/2_)7*d* | ^2^[^5^/_2_]° | 2 | 3/2)7d2[5/2]o2 | 3*s*^2^3*p*^5^(^2^P°_3/2_)4*p* | ^2^[^5^/_2_] | 2 | 3/2)4p2[5/2]2 | 524.1093 | G |
| 3*s*^2^3*p*^5^(^2^P°_3/2_)8*d* | ^2^[^5^/_2_]° | 3 | 3/2)8d2[5/2]o3 | 3*s*^2^3*p*^5^(^2^P°_3/2_)4*p* | ^2^[^3^/_2_] | 2 | 3/2)4p2[3/2]2 | 524.6198 | G |
| 3*s*^2^3*p*^5^(^2^P°_3/2_)8*d* | ^2^[^3^/_2_]° | 2 | 3/2)8d2[3/2]o2 | 3*s*^2^3*p*^5^(^2^P°_3/2_)4*p* | ^2^[^3^/_2_] | 2 | 3/2)4p2[3/2]2 | 524.9165 | G |
| 3*s*^2^3*p*^5^(^2^P°_3/2_)7*d* | ^2^[^7^/_2_]° | 3 | 3/2)7d2[7/2]o3 | 3*s*^2^3*p*^5^(^2^P°_3/2_)4*p* | ^2^[^5^/_2_] | 2 | 3/2)4p2[5/2]2 | 525.2787 | G |
| 3*s*^2^3*p*^5^(^2^P°_1/2_)6*d* | ^2^[^5^/_2_]° | 2 | 1/2)6d2[5/2]o2 | 3*s*^2^3*p*^5^(^2^P°_3/2_)4*p* | ^2^[^3^/_2_] | 1 | 3/2)4p2[3/2]1 | 525.4471 | G |
| 3*s*^2^3*p*^5^(^2^P°_1/2_)6*d* | ^2^[^5^/_2_]° | 3 | 1/2)6d2[5/2]o3 | 3*s*^2^3*p*^5^(^2^P°_3/2_)4*p* | ^2^[^3^/_2_] | 2 | 3/2)4p2[3/2]2 | 528.6069 | G |
| 3*s*^2^3*p*^5^(^2^P°_3/2_)8*d* | ^2^[^1^/_2_]° | 1 | 3/2)8d2[1/2]o1 | 3*s*^2^3*p*^5^(^2^P°_3/2_)4*p* | ^2^[^3^/_2_] | 2 | 3/2)4p2[3/2]2 | 529.0015 | G |
| 3*s*^2^3*p*^5^(^2^P°_1/2_)6*d* | ^2^[^3^/_2_]° | 2 | 1/2)6d2[3/2]o2 | 3*s*^2^3*p*^5^(^2^P°_3/2_)4*p* | ^2^[^3^/_2_] | 2 | 3/2)4p2[3/2]2 | 530.9513 | G |
| 3*s*^2^3*p*^5^(^2^P°_1/2_)7*d* | ^2^[^5^/_2_]° | 3 | 1/2)7d2[5/2]o3 | 3*s*^2^3*p*^5^(^2^P°_1/2_)4*p* | ^2^[^3^/_2_] | 2 | 1/2)4p2[3/2]2 | 531.7715 | G |
| 3*s*^2^3*p*^5^(^2^P°_3/2_)7*d* | ^2^[^5^/_2_]° | 2 | 3/2)7d2[5/2]o2 | 3*s*^2^3*p*^5^(^2^P°_3/2_)4*p* | ^2^[^3^/_2_] | 1 | 3/2)4p2[3/2]1 | 537.3494 | G |
| 3*s*^2^3*p*^5^(^2^P°_3/2_)9*s* | ^2^[^3^/_2_]° | 2 | 3/2)9s2[3/2]o2 | 3*s*^2^3*p*^5^(^2^P°_3/2_)4*p* | ^2^[^3^/_2_] | 2 | 3/2)4p2[3/2]2 | 539.3979 | G |
| 3*s*^2^3*p*^5^(^2^P°_3/2_)7*d* | ^2^[^5^/_2_]° | 3 | 3/2)7d2[5/2]o3 | 3*s*^2^3*p*^5^(^2^P°_3/2_)4*p* | ^2^[^3^/_2_] | 2 | 3/2)4p2[3/2]2 | 541.0475 | G |
| 3*s*^2^3*p*^5^(^2^P°_3/2_)8*s* | ^2^[^3^/_2_]° | 2 | 3/2)8s2[3/2]o2 | 3*s*^2^3*p*^5^(^2^P°_3/2_)4*p* | ^2^[^5^/_2_] | 3 | 3/2)4p2[5/2]3 | 542.1351 | G |
| 3*s*^2^3*p*^5^(^2^P°_3/2_)7*s* | ^2^[^3^/_2_]° | 1 | 3/2)7s2[3/2]o1 | 3*s*^2^3*p*^5^(^2^P°_3/2_)4*p* | ^2^[^1^/_2_] | 1 | 3/2)4p2[1/2]1 | 543.9989 | G |
| 3*s*^2^3*p*^5^(^2^P°_3/2_)6*d* | ^2^[^5^/_2_]° | 3 | 3/2)6d2[5/2]o3 | 3*s*^2^3*p*^5^(^2^P°_3/2_)4*p* | ^2^[^5^/_2_] | 3 | 3/2)4p2[5/2]3 | 544.2246 | G |
| 3*s*^2^3*p*^5^(^2^P°_3/2_)7*s* | ^2^[^3^/_2_]° | 2 | 3/2)7s2[3/2]o2 | 3*s*^2^3*p*^5^(^2^P°_3/2_)4*p* | ^2^[^1^/_2_] | 1 | 3/2)4p2[1/2]1 | 545.1652 | G |
| 3*s*^2^3*p*^5^(^2^P°_3/2_)8*s* | ^2^[^3^/_2_]° | 1 | 3/2)8s2[3/2]o1 | 3*s*^2^3*p*^5^(^2^P°_3/2_)4*p* | ^2^[^5^/_2_] | 2 | 3/2)4p2[5/2]2 | 545.7416 | G |
| 3*s*^2^3*p*^5^(^2^P°_3/2_)6*d* | ^2^[^7^/_2_]° | 3 | 3/2)6d2[7/2]o3 | 3*s*^2^3*p*^5^(^2^P°_3/2_)4*p* | ^2^[^5^/_2_] | 3 | 3/2)4p2[5/2]3 | 545.9651 | G |
| 3*s*^2^3*p*^5^(^2^P°_3/2_)8*s* | ^2^[^3^/_2_]° | 2 | 3/2)8s2[3/2]o2 | 3*s*^2^3*p*^5^(^2^P°_3/2_)4*p* | ^2^[^5^/_2_] | 2 | 3/2)4p2[5/2]2 | 546.7160 | G |
| 3*s*^2^3*p*^5^(^2^P°_1/2_)7*s* | ^2^[^1^/_2_]° | 1 | 1/2)7s2[1/2]o1 | 3*s*^2^3*p*^5^(^2^P°_3/2_)4*p* | ^2^[^5^/_2_] | 2 | 3/2)4p2[5/2]2 | 547.3451 | G |
| 3*s*^2^3*p*^5^(^2^P°_3/2_)6*d* | ^2^[^5^/_2_]° | 2 | 3/2)6d2[5/2]o2 | 3*s*^2^3*p*^5^(^2^P°_3/2_)4*p* | ^2^[^5^/_2_] | 2 | 3/2)4p2[5/2]2 | 549.0120 | G |
| 3*s*^2^3*p*^5^(^2^P°_1/2_)8*s* | ^2^[^1^/_2_]° | 0 | 1/2)8s2[1/2]o0 | 3*s*^2^3*p*^5^(^2^P°_1/2_)4*p* | ^2^[^3^/_2_] | 1 | 1/2)4p2[3/2]1 | 549.2080 | G |
| 3*s*^2^3*p*^5^(^2^P°_3/2_)6*d* | ^2^[^7^/_2_]° | 4 | 3/2)6d2[7/2]o4 | 3*s*^2^3*p*^5^(^2^P°_3/2_)4*p* | ^2^[^5^/_2_] | 3 | 3/2)4p2[5/2]3 | 549.5873 | G |
| 3*s*^2^3*p*^5^(^2^P°_3/2_)6*d* | ^2^[^7^/_2_]° | 3 | 3/2)6d2[7/2]o3 | 3*s*^2^3*p*^5^(^2^P°_3/2_)4*p* | ^2^[^5^/_2_] | 2 | 3/2)4p2[5/2]2 | 550.6112 | G |
| 3*s*^2^3*p*^5^(^2^P°_1/2_)5*d* | ^2^[^5^/_2_]° | 3 | 1/2)5d2[5/2]o3 | 3*s*^2^3*p*^5^(^2^P°_3/2_)4*p* | ^2^[^5^/_2_] | 3 | 3/2)4p2[5/2]3 | 552.4957 | G |
| 3*s*^2^3*p*^5^(^2^P°_3/2_)8*d* | ^2^[^1^/_2_]° | 1 | 3/2)8d2[1/2]o1 | 3*s*^2^3*p*^5^(^2^P°_3/2_)4*p* | ^2^[^1^/_2_] | 0 | 3/2)4p2[1/2]0 | 552.8961 | G |
| 3*s*^2^3*p*^5^(^2^P°_1/2_)8*s* | ^2^[^1^/_2_]° | 1 | 1/2)8s2[1/2]o1 | 3*s*^2^3*p*^5^(^2^P°_1/2_)4*p* | ^2^[^3^/_2_] | 2 | 1/2)4p2[3/2]2 | 553.4479 | G |
| 3*s*^2^3*p*^5^(^2^P°_1/2_)5*d* | ^2^[^5^/_2_]° | 2 | 1/2)5d2[5/2]o2 | 3*s*^2^3*p*^5^(^2^P°_3/2_)4*p* | ^2^[^5^/_2_] | 3 | 3/2)4p2[5/2]3 | 554.0860 | G |
| 3*s*^2^3*p*^5^(^2^P°_3/2_)8*d* | ^2^[^1^/_2_]° | 1 | 3/2)8d2[1/2]o1 | 3*s*^2^3*p*^5^(^2^P°_1/2_)4*p* | ^2^[^3^/_2_] | 1 | 1/2)4p2[3/2]1 | 555.2741 | G |
| 3*s*^2^3*p*^5^(^2^P°_3/2_)5*d* | ^2^[^3^/_2_]° | 2 | 3/2)5d2[3/2]o2 | 3*s*^2^3*p*^5^(^2^P°_3/2_)4*p* | ^2^[^1^/_2_] | 1 | 3/2)4p2[1/2]1 | 555.8702 | G |
| 3*s*^2^3*p*^5^(^2^P°_1/2_)6*d* | ^2^[^5^/_2_]° | 2 | 1/2)6d2[5/2]o2 | 3*s*^2^3*p*^5^(^2^P°_1/2_)4*p* | ^2^[^3^/_2_] | 1 | 1/2)4p2[3/2]1 | 555.9676 | G |
| 3*s*^2^3*p*^5^(^2^P°_1/2_)5*d* | ^2^[^5^/_2_]° | 3 | 1/2)5d2[5/2]o3 | 3*s*^2^3*p*^5^(^2^P°_3/2_)4*p* | ^2^[^5^/_2_] | 2 | 3/2)4p2[5/2]2 | 557.2541 | G |
| 3*s*^2^3*p*^5^(^2^P°_1/2_)6*d* | ^2^[^3^/_2_]° | 2 | 1/2)6d2[3/2]o2 | 3*s*^2^3*p*^5^(^2^P°_1/2_)4*p* | ^2^[^3^/_2_] | 1 | 1/2)4p2[3/2]1 | 557.4228 | G |
| 3*s*^2^3*p*^5^(^2^P°_1/2_)5*d* | ^2^[^3^/_2_]° | 2 | 1/2)5d2[3/2]o2 | 3*s*^2^3*p*^5^(^2^P°_3/2_)4*p* | ^2^[^5^/_2_] | 3 | 3/2)4p2[5/2]3 | 558.1871 | G |
| 3*s*^2^3*p*^5^(^2^P°_1/2_)5*d* | ^2^[^5^/_2_]° | 2 | 1/2)5d2[5/2]o2 | 3*s*^2^3*p*^5^(^2^P°_3/2_)4*p* | ^2^[^5^/_2_] | 2 | 3/2)4p2[5/2]2 | 558.8720 | G |
| 3*s*^2^3*p*^5^(^2^P°_1/2_)6*d* | ^2^[^5^/_2_]° | 3 | 1/2)6d2[5/2]o3 | 3*s*^2^3*p*^5^(^2^P°_1/2_)4*p* | ^2^[^3^/_2_] | 2 | 1/2)4p2[3/2]2 | 559.7476 | G |
| 3*s*^2^3*p*^5^(^2^P°_3/2_)5*d* | ^2^[^1^/_2_]° | 1 | 3/2)5d2[1/2]o1 | 3*s*^2^3*p*^5^(^2^P°_3/2_)4*p* | ^2^[^1^/_2_] | 1 | 3/2)4p2[1/2]1 | 560.6733 | G |
| 3*s*^2^3*p*^5^(^2^P°_1/2_)7*s* | ^2^[^1^/_2_]° | 1 | 1/2)7s2[1/2]o1 | 3*s*^2^3*p*^5^(^2^P°_3/2_)4*p* | ^2^[^3^/_2_] | 1 | 3/2)4p2[3/2]1 | 561.8014 | G |
| 3*s*^2^3*p*^5^(^2^P°_1/2_)7*s* | ^2^[^1^/_2_]° | 0 | 1/2)7s2[1/2]o0 | 3*s*^2^3*p*^5^(^2^P°_3/2_)4*p* | ^2^[^3^/_2_] | 1 | 3/2)4p2[3/2]1 | 562.0921 | G |
| 3*s*^2^3*p*^5^(^2^P°_1/2_)6*d* | ^2^[^3^/_2_]° | 2 | 1/2)6d2[3/2]o2 | 3*s*^2^3*p*^5^(^2^P°_1/2_)4*p* | ^2^[^3^/_2_] | 2 | 1/2)4p2[3/2]2 | 562.3770 | G |
| 3*s*^2^3*p*^5^(^2^P°_3/2_)6*d* | ^2^[^5^/_2_]° | 2 | 3/2)6d2[5/2]o2 | 3*s*^2^3*p*^5^(^2^P°_3/2_)4*p* | ^2^[^3^/_2_] | 1 | 3/2)4p2[3/2]1 | 563.5576 | G |
| 3*s*^2^3*p*^5^(^2^P°_3/2_)7*d* | ^2^[^3^/_2_]° | 1 | 3/2)7d2[3/2]o1 | 3*s*^2^3*p*^5^(^2^P°_3/2_)4*p* | ^2^[^1^/_2_] | 0 | 3/2)4p2[1/2]0 | 563.734 | G |
| 3*s*^2^3*p*^5^(^2^P°_3/2_)9*s* | ^2^[^3^/_2_]° | 1 | 3/2)9s2[3/2]o1 | 3*s*^2^3*p*^5^(^2^P°_3/2_)4*p* | ^2^[^1^/_2_] | 0 | 3/2)4p2[1/2]0 | 563.912 | G |
| 3*s*^2^3*p*^5^(^2^P°_3/2_)6*d* | ^2^[^3^/_2_]° | 2 | 3/2)6d2[3/2]o2 | 3*s*^2^3*p*^5^(^2^P°_3/2_)4*p* | ^2^[^3^/_2_] | 1 | 3/2)4p2[3/2]1 | 564.1376 | G |
| 3*s*^2^3*p*^5^(^2^P°_3/2_)8*s* | ^2^[^3^/_2_]° | 1 | 3/2)8s2[3/2]o1 | 3*s*^2^3*p*^5^(^2^P°_3/2_)4*p* | ^2^[^3^/_2_] | 2 | 3/2)4p2[3/2]2 | 564.8687 | G |
| 3*s*^2^3*p*^5^(^2^P°_3/2_)5*d* | ^2^[^1^/_2_]° | 0 | 3/2)5d2[1/2]o0 | 3*s*^2^3*p*^5^(^2^P°_3/2_)4*p* | ^2^[^1^/_2_] | 1 | 3/2)4p2[1/2]1 | 565.0704 | G |
| 3*s*^2^3*p*^5^(^2^P°_3/2_)8*s* | ^2^[^3^/_2_]° | 2 | 3/2)8s2[3/2]o2 | 3*s*^2^3*p*^5^(^2^P°_3/2_)4*p* | ^2^[^3^/_2_] | 2 | 3/2)4p2[3/2]2 | 565.9127 | G |
| 3*s*^2^3*p*^5^(^2^P°_3/2_)6*d* | ^2^[^5^/_2_]° | 3 | 3/2)6d2[5/2]o3 | 3*s*^2^3*p*^5^(^2^P°_3/2_)4*p* | ^2^[^3^/_2_] | 2 | 3/2)4p2[3/2]2 | 568.1899 | G |
| 3*s*^2^3*p*^5^(^2^P°_3/2_)6*d* | ^2^[^5^/_2_]° | 2 | 3/2)6d2[5/2]o2 | 3*s*^2^3*p*^5^(^2^P°_3/2_)4*p* | ^2^[^3^/_2_] | 2 | 3/2)4p2[3/2]2 | 568.3730 | G |
| 3*s*^2^3*p*^5^(^2^P°_3/2_)6*d* | ^2^[^7^/_2_]° | 3 | 3/2)6d2[7/2]o3 | 3*s*^2^3*p*^5^(^2^P°_3/2_)4*p* | ^2^[^3^/_2_] | 2 | 3/2)4p2[3/2]2 | 570.0872 | G |
| 3*s*^2^3*p*^5^(^2^P°_3/2_)7*d* | ^2^[^1^/_2_]° | 1 | 3/2)7d2[1/2]o1 | 3*s*^2^3*p*^5^(^2^P°_3/2_)4*p* | ^2^[^1^/_2_] | 0 | 3/2)4p2[1/2]0 | 571.2504 | G |
| 3*s*^2^3*p*^5^(^2^P°_1/2_)5*d* | ^2^[^5^/_2_]° | 2 | 1/2)5d2[5/2]o2 | 3*s*^2^3*p*^5^(^2^P°_3/2_)4*p* | ^2^[^3^/_2_] | 1 | 3/2)4p2[3/2]1 | 573.9519 | G |
| 3*s*^2^3*p*^5^(^2^P°_1/2_)5*d* | ^2^[^5^/_2_]° | 3 | 1/2)5d2[5/2]o3 | 3*s*^2^3*p*^5^(^2^P°_3/2_)4*p* | ^2^[^3^/_2_] | 2 | 3/2)4p2[3/2]2 | 577.2114 | G |
| 3*s*^2^3*p*^5^(^2^P°_3/2_)7*d* | ^2^[^3^/_2_]° | 2 | 3/2)7d2[3/2]o2 | 3*s*^2^3*p*^5^(^2^P°_1/2_)4*p* | ^2^[^3^/_2_] | 2 | 1/2)4p2[3/2]2 | 577.4008 | G |
| 3*s*^2^3*p*^5^(^2^P°_1/2_)5*d* | ^2^[^3^/_2_]° | 2 | 1/2)5d2[3/2]o2 | 3*s*^2^3*p*^5^(^2^P°_3/2_)4*p* | ^2^[^3^/_2_] | 1 | 3/2)4p2[3/2]1 | 578.3536 | G |
| 3*s*^2^3*p*^5^(^2^P°_1/2_)5*d* | ^2^[^5^/_2_]° | 2 | 1/2)5d2[5/2]o2 | 3*s*^2^3*p*^5^(^2^P°_3/2_)4*p* | ^2^[^3^/_2_] | 2 | 3/2)4p2[3/2]2 | 578.9474 | G |
| 3*s*^2^3*p*^5^(^2^P°_3/2_)7*d* | ^2^[^1^/_2_]° | 1 | 3/2)7d2[1/2]o1 | 3*s*^2^3*p*^5^(^2^P°_1/2_)4*p* | ^2^[^3^/_2_] | 2 | 1/2)4p2[3/2]2 | 579.0401 | G |
| 3*s*^2^3*p*^5^(^2^P°_3/2_)6*d* | ^2^[^1^/_2_]° | 1 | 3/2)6d2[1/2]o1 | 3*s*^2^3*p*^5^(^2^P°_3/2_)4*p* | ^2^[^3^/_2_] | 2 | 3/2)4p2[3/2]2 | 580.2080 | G |
| 3*s*^2^3*p*^5^(^2^P°_1/2_)5*d* | ^2^[^3^/_2_]° | 2 | 1/2)5d2[3/2]o2 | 3*s*^2^3*p*^5^(^2^P°_3/2_)4*p* | ^2^[^3^/_2_] | 2 | 3/2)4p2[3/2]2 | 583.4264 | G |
| 3*s*^2^3*p*^5^(^2^P°_3/2_)7*d* | ^2^[^3^/_2_]° | 2 | 3/2)7d2[3/2]o2 | 3*s*^2^3*p*^5^(^2^P°_1/2_)4*p* | ^2^[^1^/_2_] | 1 | 1/2)4p2[1/2]1 | 584.3779 | G |
| 3*s*^2^3*p*^5^(^2^P°_1/2_)6*s* | ^2^[^1^/_2_]° | 1 | 1/2)6s2[1/2]o1 | 3*s*^2^3*p*^5^(^2^P°_3/2_)4*p* | ^2^[^1^/_2_] | 1 | 3/2)4p2[1/2]1 | 586.0310 | G |
| 3*s*^2^3*p*^5^(^2^P°_1/2_)6*s* | ^2^[^1^/_2_]° | 0 | 1/2)6s2[1/2]o0 | 3*s*^2^3*p*^5^(^2^P°_3/2_)4*p* | ^2^[^1^/_2_] | 1 | 3/2)4p2[1/2]1 | 588.2624 | G |
| 3*s*^2^3*p*^5^(^2^P°_3/2_)7*s* | ^2^[^3^/_2_]° | 2 | 3/2)7s2[3/2]o2 | 3*s*^2^3*p*^5^(^2^P°_3/2_)4*p* | ^2^[^5^/_2_] | 3 | 3/2)4p2[5/2]3 | 588.8584 | G |
| 3*s*^2^3*p*^5^(^2^P°_1/2_)4*d* | ^2^[^3^/_2_]° | 1 | 1/2)4d2[3/2]o1 | 3*s*^2^3*p*^5^(^2^P°_3/2_)4*p* | ^2^[^1^/_2_] | 1 | 3/2)4p2[1/2]1 | 591.2085 | G |
| 3*s*^2^3*p*^5^(^2^P°_3/2_)5*d* | ^2^[^3^/_2_]° | 1 | 3/2)5d2[3/2]o1 | 3*s*^2^3*p*^5^(^2^P°_3/2_)4*p* | ^2^[^5^/_2_] | 2 | 3/2)4p2[5/2]2 | 591.6598 | G |
| 3*s*^2^3*p*^5^(^2^P°_3/2_)5*d* | ^2^[^5^/_2_]° | 3 | 3/2)5d2[5/2]o3 | 3*s*^2^3*p*^5^(^2^P°_3/2_)4*p* | ^2^[^5^/_2_] | 3 | 3/2)4p2[5/2]3 | 592.7125 | G |
| 3*s*^2^3*p*^5^(^2^P°_3/2_)7*s* | ^2^[^3^/_2_]° | 1 | 3/2)7s2[3/2]o1 | 3*s*^2^3*p*^5^(^2^P°_3/2_)4*p* | ^2^[^5^/_2_] | 2 | 3/2)4p2[5/2]2 | 592.8812 | G |
| 3*s*^2^3*p*^5^(^2^P°_1/2_)7*s* | ^2^[^1^/_2_]° | 1 | 1/2)7s2[1/2]o1 | 3*s*^2^3*p*^5^(^2^P°_3/2_)4*p* | ^2^[^1^/_2_] | 0 | 3/2)4p2[1/2]0 | 594.0855 | G |
| 3*s*^2^3*p*^5^(^2^P°_3/2_)7*s* | ^2^[^3^/_2_]° | 2 | 3/2)7s2[3/2]o2 | 3*s*^2^3*p*^5^(^2^P°_3/2_)4*p* | ^2^[^5^/_2_] | 2 | 3/2)4p2[5/2]2 | 594.2668 | G |
| 3*s*^2^3*p*^5^(^2^P°_3/2_)5*d* | ^2^[^5^/_2_]° | 2 | 3/2)5d2[5/2]o2 | 3*s*^2^3*p*^5^(^2^P°_3/2_)4*p* | ^2^[^5^/_2_] | 3 | 3/2)4p2[5/2]3 | 594.3888 | G |
| 3*s*^2^3*p*^5^(^2^P°_3/2_)8*s* | ^2^[^3^/_2_]° | 1 | 3/2)8s2[3/2]o1 | 3*s*^2^3*p*^5^(^2^P°_1/2_)4*p* | ^2^[^3^/_2_] | 1 | 1/2)4p2[3/2]1 | 594.9259 | G |
| 3*s*^2^3*p*^5^(^2^P°_1/2_)5*d* | ^2^[^3^/_2_]° | 1 | 1/2)5d2[3/2]o1 | 3*s*^2^3*p*^5^(^2^P°_3/2_)4*p* | ^2^[^1^/_2_] | 0 | 3/2)4p2[1/2]0 | 596.448 | G |
| 3*s*^2^3*p*^5^(^2^P°_1/2_)7*s* | ^2^[^1^/_2_]° | 1 | 1/2)7s2[1/2]o1 | 3*s*^2^3*p*^5^(^2^P°_1/2_)4*p* | ^2^[^3^/_2_] | 1 | 1/2)4p2[3/2]1 | 596.8320 | G |
| 3*s*^2^3*p*^5^(^2^P°_1/2_)7*s* | ^2^[^1^/_2_]° | 0 | 1/2)7s2[1/2]o0 | 3*s*^2^3*p*^5^(^2^P°_1/2_)4*p* | ^2^[^3^/_2_] | 1 | 1/2)4p2[3/2]1 | 597.1601 | G |
| 3*s*^2^3*p*^5^(^2^P°_3/2_)5*d* | ^2^[^5^/_2_]° | 3 | 3/2)5d2[5/2]o3 | 3*s*^2^3*p*^5^(^2^P°_3/2_)4*p* | ^2^[^5^/_2_] | 2 | 3/2)4p2[5/2]2 | 598.1923 | G |
| 3*s*^2^3*p*^5^(^2^P°_3/2_)5*d* | ^2^[^7^/_2_]° | 3 | 3/2)5d2[7/2]o3 | 3*s*^2^3*p*^5^(^2^P°_3/2_)4*p* | ^2^[^5^/_2_] | 3 | 3/2)4p2[5/2]3 | 598.7301 | G |
| 3*s*^2^3*p*^5^(^2^P°_3/2_)6*d* | ^2^[^5^/_2_]° | 2 | 3/2)6d2[5/2]o2 | 3*s*^2^3*p*^5^(^2^P°_1/2_)4*p* | ^2^[^3^/_2_] | 1 | 1/2)4p2[3/2]1 | 598.8144 | G |
| 3*s*^2^3*p*^5^(^2^P°_3/2_)6*d* | ^2^[^3^/_2_]° | 2 | 3/2)6d2[3/2]o2 | 3*s*^2^3*p*^5^(^2^P°_1/2_)4*p* | ^2^[^3^/_2_] | 1 | 1/2)4p2[3/2]1 | 599.4692 | G |
| 3*s*^2^3*p*^5^(^2^P°_3/2_)5*d* | ^2^[^5^/_2_]° | 2 | 3/2)5d2[5/2]o2 | 3*s*^2^3*p*^5^(^2^P°_3/2_)4*p* | ^2^[^5^/_2_] | 2 | 3/2)4p2[5/2]2 | 599.8998 | G |
| 3*s*^2^3*p*^5^(^2^P°_3/2_)8*s* | ^2^[^3^/_2_]° | 1 | 3/2)8s2[3/2]o1 | 3*s*^2^3*p*^5^(^2^P°_1/2_)4*p* | ^2^[^3^/_2_] | 2 | 1/2)4p2[3/2]2 | 600.5725 | R |
| 3*s*^2^3*p*^5^(^2^P°_3/2_)5*d* | ^2^[^3^/_2_]° | 2 | 3/2)5d2[3/2]o2 | 3*s*^2^3*p*^5^(^2^P°_3/2_)4*p* | ^2^[^5^/_2_] | 3 | 3/2)4p2[5/2]3 | 601.3677 | R |
| 3*s*^2^3*p*^5^(^2^P°_1/2_)7*s* | ^2^[^1^/_2_]° | 1 | 1/2)7s2[1/2]o1 | 3*s*^2^3*p*^5^(^2^P°_1/2_)4*p* | ^2^[^3^/_2_] | 2 | 1/2)4p2[3/2]2 | 602.5150 | R |
| 3*s*^2^3*p*^5^(^2^P°_3/2_)5*d* | ^2^[^7^/_2_]° | 4 | 3/2)5d2[7/2]o4 | 3*s*^2^3*p*^5^(^2^P°_3/2_)4*p* | ^2^[^5^/_2_] | 3 | 3/2)4p2[5/2]3 | 603.2127 | R |
| 3*s*^2^3*p*^5^(^2^P°_3/2_)5*d* | ^2^[^7^/_2_]° | 3 | 3/2)5d2[7/2]o3 | 3*s*^2^3*p*^5^(^2^P°_3/2_)4*p* | ^2^[^5^/_2_] | 2 | 3/2)4p2[5/2]2 | 604.3223 | R |
| 3*s*^2^3*p*^5^(^2^P°_1/2_)4*d* | ^2^[^5^/_2_]° | 2 | 1/2)4d2[5/2]o2 | 3*s*^2^3*p*^5^(^2^P°_3/2_)4*p* | ^2^[^1^/_2_] | 1 | 3/2)4p2[1/2]1 | 605.2723 | R |
| 3*s*^2^3*p*^5^(^2^P°_1/2_)4*d* | ^2^[^3^/_2_]° | 2 | 1/2)4d2[3/2]o2 | 3*s*^2^3*p*^5^(^2^P°_3/2_)4*p* | ^2^[^1^/_2_] | 1 | 3/2)4p2[1/2]1 | 605.9372 | R |
| 3*s*^2^3*p*^5^(^2^P°_3/2_)6*d* | ^2^[^7^/_2_]° | 3 | 3/2)6d2[7/2]o3 | 3*s*^2^3*p*^5^(^2^P°_1/2_)4*p* | ^2^[^3^/_2_] | 2 | 1/2)4p2[3/2]2 | 606.4750 | R |
| 3*s*^2^3*p*^5^(^2^P°_3/2_)8*s* | ^2^[^3^/_2_]° | 1 | 3/2)8s2[3/2]o1 | 3*s*^2^3*p*^5^(^2^P°_1/2_)4*p* | ^2^[^1^/_2_] | 1 | 1/2)4p2[1/2]1 | 608.1244 | R |
| 3*s*^2^3*p*^5^(^2^P°_3/2_)5*d* | ^2^[^3^/_2_]° | 1 | 3/2)5d2[3/2]o1 | 3*s*^2^3*p*^5^(^2^P°_3/2_)4*p* | ^2^[^3^/_2_] | 1 | 3/2)4p2[3/2]1 | 608.5879 | R |
| 3*s*^2^3*p*^5^(^2^P°_3/2_)6*d* | ^2^[^1^/_2_]° | 1 | 3/2)6d2[1/2]o1 | 3*s*^2^3*p*^5^(^2^P°_3/2_)4*p* | ^2^[^1^/_2_] | 0 | 3/2)4p2[1/2]0 | 609.0785 | R |
| 3*s*^2^3*p*^5^(^2^P°_3/2_)7*s* | ^2^[^3^/_2_]° | 1 | 3/2)7s2[3/2]o1 | 3*s*^2^3*p*^5^(^2^P°_3/2_)4*p* | ^2^[^3^/_2_] | 1 | 3/2)4p2[3/2]1 | 609.8803 | R |
| 3*s*^2^3*p*^5^(^2^P°_1/2_)7*s* | ^2^[^1^/_2_]° | 1 | 1/2)7s2[1/2]o1 | 3*s*^2^3*p*^5^(^2^P°_1/2_)4*p* | ^2^[^1^/_2_] | 1 | 1/2)4p2[1/2]1 | 610.1161 | R |
| 3*s*^2^3*p*^5^(^2^P°_1/2_)7*s* | ^2^[^1^/_2_]° | 0 | 1/2)7s2[1/2]o0 | 3*s*^2^3*p*^5^(^2^P°_1/2_)4*p* | ^2^[^1^/_2_] | 1 | 1/2)4p2[1/2]1 | 610.4590 | R |
| 3*s*^2^3*p*^5^(^2^P°_1/2_)5*d* | ^2^[^5^/_2_]° | 2 | 1/2)5d2[5/2]o2 | 3*s*^2^3*p*^5^(^2^P°_1/2_)4*p* | ^2^[^3^/_2_] | 1 | 1/2)4p2[3/2]1 | 610.5635 | R |
| 3*s*^2^3*p*^5^(^2^P°_3/2_)7*s* | ^2^[^3^/_2_]° | 2 | 3/2)7s2[3/2]o2 | 3*s*^2^3*p*^5^(^2^P°_3/2_)4*p* | ^2^[^3^/_2_] | 1 | 3/2)4p2[3/2]1 | 611.3466 | R |
| 3*s*^2^3*p*^5^(^2^P°_3/2_)6*d* | ^2^[^1^/_2_]° | 1 | 3/2)6d2[1/2]o1 | 3*s*^2^3*p*^5^(^2^P°_1/2_)4*p* | ^2^[^3^/_2_] | 1 | 1/2)4p2[3/2]1 | 611.9656 | R |
| 3*s*^2^3*p*^5^(^2^P°_3/2_)6*d* | ^2^[^5^/_2_]° | 2 | 3/2)6d2[5/2]o2 | 3*s*^2^3*p*^5^(^2^P°_1/2_)4*p* | ^2^[^1^/_2_] | 1 | 1/2)4p2[1/2]1 | 612.1879 | R |
| 3*s*^2^3*p*^5^(^2^P°_3/2_)5*d* | ^2^[^1^/_2_]° | 1 | 3/2)5d2[1/2]o1 | 3*s*^2^3*p*^5^(^2^P°_3/2_)4*p* | ^2^[^5^/_2_] | 2 | 3/2)4p2[5/2]2 | 612.7416 | R |
| 3*s*^2^3*p*^5^(^2^P°_3/2_)6*d* | ^2^[^3^/_2_]° | 2 | 3/2)6d2[3/2]o2 | 3*s*^2^3*p*^5^(^2^P°_1/2_)4*p* | ^2^[^1^/_2_] | 1 | 1/2)4p2[1/2]1 | 612.8724 | R |
| 3*s*^2^3*p*^5^(^2^P°_1/2_)5*d* | ^2^[^5^/_2_]° | 3 | 1/2)5d2[5/2]o3 | 3*s*^2^3*p*^5^(^2^P°_1/2_)4*p* | ^2^[^3^/_2_] | 2 | 1/2)4p2[3/2]2 | 614.5441 | R |
| 3*s*^2^3*p*^5^(^2^P°_3/2_)7*s* | ^2^[^3^/_2_]° | 1 | 3/2)7s2[3/2]o1 | 3*s*^2^3*p*^5^(^2^P°_3/2_)4*p* | ^2^[^3^/_2_] | 2 | 3/2)4p2[3/2]2 | 615.5238 | R |
| 3*s*^2^3*p*^5^(^2^P°_1/2_)5*d* | ^2^[^5^/_2_]° | 2 | 1/2)5d2[5/2]o2 | 3*s*^2^3*p*^5^(^2^P°_1/2_)4*p* | ^2^[^3^/_2_] | 2 | 1/2)4p2[3/2]2 | 616.5123 | R |
| 3*s*^2^3*p*^5^(^2^P°_3/2_)7*s* | ^2^[^3^/_2_]° | 2 | 3/2)7s2[3/2]o2 | 3*s*^2^3*p*^5^(^2^P°_3/2_)4*p* | ^2^[^3^/_2_] | 2 | 3/2)4p2[3/2]2 | 617.0174 | R |
| 3*s*^2^3*p*^5^(^2^P°_3/2_)5*d* | ^2^[^5^/_2_]° | 2 | 3/2)5d2[5/2]o2 | 3*s*^2^3*p*^5^(^2^P°_3/2_)4*p* | ^2^[^3^/_2_] | 1 | 3/2)4p2[3/2]1 | 617.3096 | R |
| 3*s*^2^3*p*^5^(^2^P°_3/2_)6*d* | ^2^[^1^/_2_]° | 1 | 3/2)6d2[1/2]o1 | 3*s*^2^3*p*^5^(^2^P°_1/2_)4*p* | ^2^[^3^/_2_] | 2 | 1/2)4p2[3/2]2 | 617.9419 | R |
| 3*s*^2^3*p*^5^(^2^P°_3/2_)5*d* | ^2^[^5^/_2_]° | 3 | 3/2)5d2[5/2]o3 | 3*s*^2^3*p*^5^(^2^P°_3/2_)4*p* | ^2^[^3^/_2_] | 2 | 3/2)4p2[3/2]2 | 621.2503 | R |
| 3*s*^2^3*p*^5^(^2^P°_1/2_)5*d* | ^2^[^3^/_2_]° | 2 | 1/2)5d2[3/2]o2 | 3*s*^2^3*p*^5^(^2^P°_1/2_)4*p* | ^2^[^3^/_2_] | 2 | 1/2)4p2[3/2]2 | 621.5939 | R |
| 3*s*^2^3*p*^5^(^2^P°_3/2_)5*d* | ^2^[^5^/_2_]° | 2 | 3/2)5d2[5/2]o2 | 3*s*^2^3*p*^5^(^2^P°_3/2_)4*p* | ^2^[^3^/_2_] | 2 | 3/2)4p2[3/2]2 | 623.0921 | R |
| 3*s*^2^3*p*^5^(^2^P°_3/2_)6*d* | ^2^[^1^/_2_]° | 0 | 3/2)6d2[1/2]o0 | 3*s*^2^3*p*^5^(^2^P°_1/2_)4*p* | ^2^[^1^/_2_] | 1 | 1/2)4p2[1/2]1 | 624.3390 | R |
| 3*s*^2^3*p*^5^(^2^P°_1/2_)5*d* | ^2^[^5^/_2_]° | 2 | 1/2)5d2[5/2]o2 | 3*s*^2^3*p*^5^(^2^P°_1/2_)4*p* | ^2^[^1^/_2_] | 1 | 1/2)4p2[1/2]1 | 624.4730 | R |
| 3*s*^2^3*p*^5^(^2^P°_3/2_)5*d* | ^2^[^3^/_2_]° | 2 | 3/2)5d2[3/2]o2 | 3*s*^2^3*p*^5^(^2^P°_3/2_)4*p* | ^2^[^3^/_2_] | 1 | 3/2)4p2[3/2]1 | 624.8406 | R |
| 3*s*^2^3*p*^5^(^2^P°_3/2_)5*d* | ^2^[^7^/_2_]° | 3 | 3/2)5d2[7/2]o3 | 3*s*^2^3*p*^5^(^2^P°_3/2_)4*p* | ^2^[^3^/_2_] | 2 | 3/2)4p2[3/2]2 | 627.8645 | R |
| 3*s*^2^3*p*^5^(^2^P°_1/2_)5*d* | ^2^[^3^/_2_]° | 2 | 1/2)5d2[3/2]o2 | 3*s*^2^3*p*^5^(^2^P°_1/2_)4*p* | ^2^[^1^/_2_] | 1 | 1/2)4p2[1/2]1 | 629.6873 | R |
| 3*s*^2^3*p*^5^(^2^P°_3/2_)5*d* | ^2^[^3^/_2_]° | 2 | 3/2)5d2[3/2]o2 | 3*s*^2^3*p*^5^(^2^P°_3/2_)4*p* | ^2^[^3^/_2_] | 2 | 3/2)4p2[3/2]2 | 630.7657 | R |
| 3*s*^2^3*p*^5^(^2^P°_3/2_)5*d* | ^2^[^1^/_2_]° | 1 | 3/2)5d2[1/2]o1 | 3*s*^2^3*p*^5^(^2^P°_3/2_)4*p* | ^2^[^3^/_2_] | 1 | 3/2)4p2[3/2]1 | 630.9160 | R |
| 3*s*^2^3*p*^5^(^2^P°_3/2_)5*d* | ^2^[^1^/_2_]° | 0 | 3/2)5d2[1/2]o0 | 3*s*^2^3*p*^5^(^2^P°_3/2_)4*p* | ^2^[^3^/_2_] | 1 | 3/2)4p2[3/2]1 | 636.4894 | R |
| 3*s*^2^3*p*^5^(^2^P°_3/2_)5*d* | ^2^[^1^/_2_]° | 1 | 3/2)5d2[1/2]o1 | 3*s*^2^3*p*^5^(^2^P°_3/2_)4*p* | ^2^[^3^/_2_] | 2 | 3/2)4p2[3/2]2 | 636.9575 | R |
| 3*s*^2^3*p*^5^(^2^P°_3/2_)6*s* | ^2^[^3^/_2_]° | 1 | 3/2)6s2[3/2]o1 | 3*s*^2^3*p*^5^(^2^P°_3/2_)4*p* | ^2^[^1^/_2_] | 1 | 3/2)4p2[1/2]1 | 638.4717 | R |
| 3*s*^2^3*p*^5^(^2^P°_3/2_)6*s* | ^2^[^3^/_2_]° | 2 | 3/2)6s2[3/2]o2 | 3*s*^2^3*p*^5^(^2^P°_3/2_)4*p* | ^2^[^1^/_2_] | 1 | 3/2)4p2[1/2]1 | 641.6307 | R |
| 3*s*^2^3*p*^5^(^2^P°_1/2_)6*s* | ^2^[^1^/_2_]° | 1 | 1/2)6s2[1/2]o1 | 3*s*^2^3*p*^5^(^2^P°_3/2_)4*p* | ^2^[^5^/_2_] | 2 | 3/2)4p2[5/2]2 | 643.1555 | R |
| 3*s*^2^3*p*^5^(^2^P°_3/2_)5*d* | ^2^[^3^/_2_]° | 1 | 3/2)5d2[3/2]o1 | 3*s*^2^3*p*^5^(^2^P°_3/2_)4*p* | ^2^[^1^/_2_] | 0 | 3/2)4p2[1/2]0 | 646.6552 | R |
| 3*s*^2^3*p*^5^(^2^P°_3/2_)7*s* | ^2^[^3^/_2_]° | 1 | 3/2)7s2[3/2]o1 | 3*s*^2^3*p*^5^(^2^P°_3/2_)4*p* | ^2^[^1^/_2_] | 0 | 3/2)4p2[1/2]0 | 648.1145 | R |
| 3*s*^2^3*p*^5^(^2^P°_1/2_)4*d* | ^2^[^3^/_2_]° | 1 | 1/2)4d2[3/2]o1 | 3*s*^2^3*p*^5^(^2^P°_3/2_)4*p* | ^2^[^5^/_2_] | 2 | 3/2)4p2[5/2]2 | 649.3969 | R |
| 3*s*^2^3*p*^5^(^2^P°_3/2_)7*s* | ^2^[^3^/_2_]° | 1 | 3/2)7s2[3/2]o1 | 3*s*^2^3*p*^5^(^2^P°_1/2_)4*p* | ^2^[^3^/_2_] | 1 | 1/2)4p2[3/2]1 | 651.3846 | R |
| 3*s*^2^3*p*^5^(^2^P°_1/2_)4*d* | ^2^[^5^/_2_]° | 3 | 1/2)4d2[5/2]o3 | 3*s*^2^3*p*^5^(^2^P°_3/2_)4*p* | ^2^[^5^/_2_] | 3 | 3/2)4p2[5/2]3 | 653.8112 | R |
| 3*s*^2^3*p*^5^(^2^P°_1/2_)4*d* | ^2^[^5^/_2_]° | 2 | 1/2)4d2[5/2]o2 | 3*s*^2^3*p*^5^(^2^P°_3/2_)4*p* | ^2^[^5^/_2_] | 3 | 3/2)4p2[5/2]3 | 659.6113 | R |
| 3*s*^2^3*p*^5^(^2^P°_3/2_)7*s* | ^2^[^3^/_2_]° | 2 | 3/2)7s2[3/2]o2 | 3*s*^2^3*p*^5^(^2^P°_1/2_)4*p* | ^2^[^3^/_2_] | 2 | 1/2)4p2[3/2]2 | 659.8678 | R |
| 3*s*^2^3*p*^5^(^2^P°_1/2_)4*d* | ^2^[^3^/_2_]° | 2 | 1/2)4d2[3/2]o2 | 3*s*^2^3*p*^5^(^2^P°_3/2_)4*p* | ^2^[^5^/_2_] | 3 | 3/2)4p2[5/2]3 | 660.4011 | R |
| 3*s*^2^3*p*^5^(^2^P°_1/2_)4*d* | ^2^[^5^/_2_]° | 3 | 1/2)4d2[5/2]o3 | 3*s*^2^3*p*^5^(^2^P°_3/2_)4*p* | ^2^[^5^/_2_] | 2 | 3/2)4p2[5/2]2 | 660.4853 | R |
| 3*s*^2^3*p*^5^(^2^P°_1/2_)6*s* | ^2^[^1^/_2_]° | 1 | 1/2)6s2[1/2]o1 | 3*s*^2^3*p*^5^(^2^P°_3/2_)4*p* | ^2^[^3^/_2_] | 1 | 3/2)4p2[3/2]1 | 663.2084 | R |
| 3*s*^2^3*p*^5^(^2^P°_3/2_)5*d* | ^2^[^3^/_2_]° | 1 | 3/2)5d2[3/2]o1 | 3*s*^2^3*p*^5^(^2^P°_1/2_)4*p* | ^2^[^1^/_2_] | 1 | 1/2)4p2[1/2]1 | 665.6938 | R |
| 3*s*^2^3*p*^5^(^2^P°_1/2_)6*s* | ^2^[^1^/_2_]° | 0 | 1/2)6s2[1/2]o0 | 3*s*^2^3*p*^5^(^2^P°_3/2_)4*p* | ^2^[^3^/_2_] | 1 | 3/2)4p2[3/2]1 | 666.0677 | R |
| 3*s*^2^3*p*^5^(^2^P°_1/2_)4*d* | ^2^[^5^/_2_]° | 2 | 1/2)4d2[5/2]o2 | 3*s*^2^3*p*^5^(^2^P°_3/2_)4*p* | ^2^[^5^/_2_] | 2 | 3/2)4p2[5/2]2 | 666.4051 | R |
| 3*s*^2^3*p*^5^(^2^P°_1/2_)4*p* | ^2^[^1^/_2_] | 0 | 1/2)4p2[1/2]0 | 3*s*^2^3*p*^5^(^2^P°_3/2_)4*s* | ^2^[^3^/_2_]° | 1 | 3/2)4s2[3/2]o1 | 667.7281 | R |
| 3*s*^2^3*p*^5^(^2^P°_3/2_)5*d* | ^2^[^3^/_2_]° | 2 | 3/2)5d2[3/2]o2 | 3*s*^2^3*p*^5^(^2^P°_1/2_)4*p* | ^2^[^3^/_2_] | 1 | 1/2)4p2[3/2]1 | 668.4788 | R |
| 3*s*^2^3*p*^5^(^2^P°_1/2_)4*d* | ^2^[^3^/_2_]° | 1 | 1/2)4d2[3/2]o1 | 3*s*^2^3*p*^5^(^2^P°_3/2_)4*p* | ^2^[^3^/_2_] | 1 | 3/2)4p2[3/2]1 | 669.8471 | R |
| 3*s*^2^3*p*^5^(^2^P°_1/2_)6*s* | ^2^[^1^/_2_]° | 1 | 1/2)6s2[1/2]o1 | 3*s*^2^3*p*^5^(^2^P°_3/2_)4*p* | ^2^[^3^/_2_] | 2 | 3/2)4p2[3/2]2 | 669.8874 | R |
| 3*s*^2^3*p*^5^(^2^P°_3/2_)5*d* | ^2^[^1^/_2_]° | 1 | 3/2)5d2[1/2]o1 | 3*s*^2^3*p*^5^(^2^P°_3/2_)4*p* | ^2^[^1^/_2_] | 0 | 3/2)4p2[1/2]0 | 671.9219 | R |
| 3*s*^2^3*p*^5^(^2^P°_3/2_)5*d* | ^2^[^7^/_2_]° | 3 | 3/2)5d2[7/2]o3 | 3*s*^2^3*p*^5^(^2^P°_1/2_)4*p* | ^2^[^3^/_2_] | 2 | 1/2)4p2[3/2]2 | 672.2890 | R |
| 3*s*^2^3*p*^5^(^2^P°_3/2_)4*d* | ^2^[^3^/_2_]° | 2 | 3/2)4d2[3/2]o2 | 3*s*^2^3*p*^5^(^2^P°_3/2_)4*p* | ^2^[^1^/_2_] | 1 | 3/2)4p2[1/2]1 | 675.2834 | R |
| 3*s*^2^3*p*^5^(^2^P°_3/2_)5*d* | ^2^[^1^/_2_]° | 1 | 3/2)5d2[1/2]o1 | 3*s*^2^3*p*^5^(^2^P°_1/2_)4*p* | ^2^[^3^/_2_] | 1 | 1/2)4p2[3/2]1 | 675.4372 | R |
| 3*s*^2^3*p*^5^(^2^P°_3/2_)5*d* | ^2^[^3^/_2_]° | 2 | 3/2)5d2[3/2]o2 | 3*s*^2^3*p*^5^(^2^P°_1/2_)4*p* | ^2^[^3^/_2_] | 2 | 1/2)4p2[3/2]2 | 675.6163 | R |
| 3*s*^2^3*p*^5^(^2^P°_1/2_)4*d* | ^2^[^3^/_2_]° | 1 | 1/2)4d2[3/2]o1 | 3*s*^2^3*p*^5^(^2^P°_3/2_)4*p* | ^2^[^3^/_2_] | 2 | 3/2)4p2[3/2]2 | 676.6611 | R |
| 3*s*^2^3*p*^5^(^2^P°_3/2_)6*d* | ^2^[^1^/_2_]° | 1 | 3/2)6d2[1/2]o1 | 3*s*^2^3*p*^5^(^2^P°_1/2_)4*p* | ^2^[^1^/_2_] | 0 | 1/2)4p2[1/2]0 | 677.9926 | R |
| 3*s*^2^3*p*^5^(^2^P°_3/2_)5*d* | ^2^[^1^/_2_]° | 0 | 3/2)5d2[1/2]o0 | 3*s*^2^3*p*^5^(^2^P°_1/2_)4*p* | ^2^[^3^/_2_] | 1 | 1/2)4p2[3/2]1 | 681.8289 | R |
| 3*s*^2^3*p*^5^(^2^P°_3/2_)5*d* | ^2^[^1^/_2_]° | 1 | 3/2)5d2[1/2]o1 | 3*s*^2^3*p*^5^(^2^P°_1/2_)4*p* | ^2^[^3^/_2_] | 2 | 1/2)4p2[3/2]2 | 682.7249 | R |
| 3*s*^2^3*p*^5^(^2^P°_3/2_)5*d* | ^2^[^3^/_2_]° | 2 | 3/2)5d2[3/2]o2 | 3*s*^2^3*p*^5^(^2^P°_1/2_)4*p* | ^2^[^1^/_2_] | 1 | 1/2)4p2[1/2]1 | 685.1884 | R |
| 3*s*^2^3*p*^5^(^2^P°_3/2_)4*d* | ^2^[^1^/_2_]° | 1 | 3/2)4d2[1/2]o1 | 3*s*^2^3*p*^5^(^2^P°_3/2_)4*p* | ^2^[^1^/_2_] | 1 | 3/2)4p2[1/2]1 | 687.1289 | R |
| 3*s*^2^3*p*^5^(^2^P°_1/2_)4*d* | ^2^[^5^/_2_]° | 2 | 1/2)4d2[5/2]o2 | 3*s*^2^3*p*^5^(^2^P°_3/2_)4*p* | ^2^[^3^/_2_] | 1 | 3/2)4p2[3/2]1 | 687.9583 | R |
| 3*s*^2^3*p*^5^(^2^P°_1/2_)4*d* | ^2^[^5^/_2_]° | 3 | 1/2)4d2[5/2]o3 | 3*s*^2^3*p*^5^(^2^P°_3/2_)4*p* | ^2^[^3^/_2_] | 2 | 3/2)4p2[3/2]2 | 688.7088 | R |
| 3*s*^2^3*p*^5^(^2^P°_1/2_)4*d* | ^2^[^3^/_2_]° | 2 | 1/2)4d2[3/2]o2 | 3*s*^2^3*p*^5^(^2^P°_3/2_)4*p* | ^2^[^3^/_2_] | 1 | 3/2)4p2[3/2]1 | 688.8174 | R |
| 3*s*^2^3*p*^5^(^2^P°_3/2_)5*d* | ^2^[^1^/_2_]° | 1 | 3/2)5d2[1/2]o1 | 3*s*^2^3*p*^5^(^2^P°_1/2_)4*p* | ^2^[^1^/_2_] | 1 | 1/2)4p2[1/2]1 | 692.5009 | R |
| 3*s*^2^3*p*^5^(^2^P°_3/2_)4*d* | ^2^[^1^/_2_]° | 0 | 3/2)4d2[1/2]o0 | 3*s*^2^3*p*^5^(^2^P°_3/2_)4*p* | ^2^[^1^/_2_] | 1 | 3/2)4p2[1/2]1 | 693.7664 | R |
| 3*s*^2^3*p*^5^(^2^P°_1/2_)4*d* | ^2^[^5^/_2_]° | 2 | 1/2)4d2[5/2]o2 | 3*s*^2^3*p*^5^(^2^P°_3/2_)4*p* | ^2^[^3^/_2_] | 2 | 3/2)4p2[3/2]2 | 695.1477 | R |
| 3*s*^2^3*p*^5^(^2^P°_1/2_)4*d* | ^2^[^3^/_2_]° | 2 | 1/2)4d2[3/2]o2 | 3*s*^2^3*p*^5^(^2^P°_3/2_)4*p* | ^2^[^3^/_2_] | 2 | 3/2)4p2[3/2]2 | 696.0250 | R |
| 3*s*^2^3*p*^5^(^2^P°_1/2_)4*p* | ^2^[^1^/_2_] | 1 | 1/2)4p2[1/2]1 | 3*s*^2^3*p*^5^(^2^P°_3/2_)4*s* | ^2^[^3^/_2_]° | 2 | 3/2)4s2[3/2]o2 | 696.5430 | R |
| 3*s*^2^3*p*^5^(^2^P°_3/2_)5*d* | ^2^[^1^/_2_]° | 0 | 3/2)5d2[1/2]o0 | 3*s*^2^3*p*^5^(^2^P°_1/2_)4*p* | ^2^[^1^/_2_] | 1 | 1/2)4p2[1/2]1 | 699.2212 | R |
| 3*s*^2^3*p*^5^(^2^P°_3/2_)6*s* | ^2^[^3^/_2_]° | 2 | 3/2)6s2[3/2]o2 | 3*s*^2^3*p*^5^(^2^P°_3/2_)4*p* | ^2^[^5^/_2_] | 3 | 3/2)4p2[5/2]3 | 703.0251 | IR |
| 3*s*^2^3*p*^5^(^2^P°_1/2_)4*p* | ^2^[^3^/_2_] | 2 | 1/2)4p2[3/2]2 | 3*s*^2^3*p*^5^(^2^P°_3/2_)4*s* | ^2^[^3^/_2_]° | 2 | 3/2)4s2[3/2]o2 | 706.7217 | IR |
| 3*s*^2^3*p*^5^(^2^P°_3/2_)6*s* | ^2^[^3^/_2_]° | 1 | 3/2)6s2[3/2]o1 | 3*s*^2^3*p*^5^(^2^P°_3/2_)4*p* | ^2^[^5^/_2_] | 2 | 3/2)4p2[5/2]2 | 706.8735 | IR |
| 3*s*^2^3*p*^5^(^2^P°_1/2_)6*s* | ^2^[^1^/_2_]° | 1 | 1/2)6s2[1/2]o1 | 3*s*^2^3*p*^5^(^2^P°_3/2_)4*p* | ^2^[^1^/_2_] | 0 | 3/2)4p2[1/2]0 | 708.6705 | IR |
| 3*s*^2^3*p*^5^(^2^P°_3/2_)6*s* | ^2^[^3^/_2_]° | 2 | 3/2)6s2[3/2]o2 | 3*s*^2^3*p*^5^(^2^P°_3/2_)4*p* | ^2^[^5^/_2_] | 2 | 3/2)4p2[5/2]2 | 710.7477 | IR |
| 3*s*^2^3*p*^5^(^2^P°_1/2_)6*s* | ^2^[^1^/_2_]° | 1 | 1/2)6s2[1/2]o1 | 3*s*^2^3*p*^5^(^2^P°_1/2_)4*p* | ^2^[^3^/_2_] | 1 | 1/2)4p2[3/2]1 | 712.5820 | IR |
| 3*s*^2^3*p*^5^(^2^P°_1/2_)4*p* | ^2^[^3^/_2_] | 1 | 1/2)4p2[3/2]1 | 3*s*^2^3*p*^5^(^2^P°_3/2_)4*s* | ^2^[^3^/_2_]° | 2 | 3/2)4s2[3/2]o2 | 714.7041 | IR |
| 3*s*^2^3*p*^5^(^2^P°_1/2_)6*s* | ^2^[^1^/_2_]° | 0 | 1/2)6s2[1/2]o0 | 3*s*^2^3*p*^5^(^2^P°_1/2_)4*p* | ^2^[^3^/_2_] | 1 | 1/2)4p2[3/2]1 | 715.8839 | IR |
| 3*s*^2^3*p*^5^(^2^P°_1/2_)4*d* | ^2^[^3^/_2_]° | 1 | 1/2)4d2[3/2]o1 | 3*s*^2^3*p*^5^(^2^P°_3/2_)4*p* | ^2^[^1^/_2_] | 0 | 3/2)4p2[1/2]0 | 716.2557 | IR |
| 3*s*^2^3*p*^5^(^2^P°_1/2_)6*s* | ^2^[^1^/_2_]° | 1 | 1/2)6s2[1/2]o1 | 3*s*^2^3*p*^5^(^2^P°_1/2_)4*p* | ^2^[^3^/_2_] | 2 | 1/2)4p2[3/2]2 | 720.6980 | IR |
| 3*s*^2^3*p*^5^(^2^P°_3/2_)4*d* | ^2^[^5^/_2_]° | 2 | 3/2)4d2[5/2]o2 | 3*s*^2^3*p*^5^(^2^P°_3/2_)4*p* | ^2^[^5^/_2_] | 2 | 3/2)4p2[5/2]2 | 722.994 | IR |
| 3*s*^2^3*p*^5^(^2^P°_3/2_)4*d* | ^2^[^3^/_2_]° | 1 | 3/2)4d2[3/2]o1 | 3*s*^2^3*p*^5^(^2^P°_3/2_)4*p* | ^2^[^3^/_2_] | 1 | 3/2)4p2[3/2]1 | 726.517 | IR |
| 3*s*^2^3*p*^5^(^2^P°_3/2_)4*d* | ^2^[^7^/_2_]° | 3 | 3/2)4d2[7/2]o3 | 3*s*^2^3*p*^5^(^2^P°_3/2_)4*p* | ^2^[^5^/_2_] | 3 | 3/2)4p2[5/2]3 | 727.067 | IR |
| 3*s*^2^3*p*^5^(^2^P°_1/2_)4*p* | ^2^[^1^/_2_] | 1 | 1/2)4p2[1/2]1 | 3*s*^2^3*p*^5^(^2^P°_3/2_)4*s* | ^2^[^3^/_2_]° | 1 | 3/2)4s2[3/2]o1 | 727.2935 | IR |
| 3*s*^2^3*p*^5^(^2^P°_1/2_)4*d* | ^2^[^3^/_2_]° | 1 | 1/2)4d2[3/2]o1 | 3*s*^2^3*p*^5^(^2^P°_1/2_)4*p* | ^2^[^3^/_2_] | 2 | 1/2)4p2[3/2]2 | 728.5443 | IR |
| 3*s*^2^3*p*^5^(^2^P°_3/2_)6*s* | ^2^[^3^/_2_]° | 1 | 3/2)6s2[3/2]o1 | 3*s*^2^3*p*^5^(^2^P°_3/2_)4*p* | ^2^[^3^/_2_] | 1 | 3/2)4p2[3/2]1 | 731.1716 | IR |
| 3*s*^2^3*p*^5^(^2^P°_1/2_)6*s* | ^2^[^1^/_2_]° | 1 | 1/2)6s2[1/2]o1 | 3*s*^2^3*p*^5^(^2^P°_1/2_)4*p* | ^2^[^1^/_2_] | 1 | 1/2)4p2[1/2]1 | 731.6005 | IR |
| 3*s*^2^3*p*^5^(^2^P°_1/2_)6*s* | ^2^[^1^/_2_]° | 0 | 1/2)6s2[1/2]o0 | 3*s*^2^3*p*^5^(^2^P°_1/2_)4*p* | ^2^[^1^/_2_] | 1 | 1/2)4p2[1/2]1 | 735.0814 | IR |
| 3*s*^2^3*p*^5^(^2^P°_3/2_)6*s* | ^2^[^3^/_2_]° | 2 | 3/2)6s2[3/2]o2 | 3*s*^2^3*p*^5^(^2^P°_3/2_)4*p* | ^2^[^3^/_2_] | 1 | 3/2)4p2[3/2]1 | 735.3175 | IR |
| 3*s*^2^3*p*^5^(^2^P°_3/2_)4*d* | ^2^[^7^/_2_]° | 3 | 3/2)4d2[7/2]o3 | 3*s*^2^3*p*^5^(^2^P°_3/2_)4*p* | ^2^[^5^/_2_] | 2 | 3/2)4p2[5/2]2 | 735.330 | IR |
| 3*s*^2^3*p*^5^(^2^P°_3/2_)4*d* | ^2^[^7^/_2_]° | 4 | 3/2)4d2[7/2]o4 | 3*s*^2^3*p*^5^(^2^P°_3/2_)4*p* | ^2^[^5^/_2_] | 3 | 3/2)4p2[5/2]3 | 737.2117 | IR |
| 3*s*^2^3*p*^5^(^2^P°_1/2_)4*p* | ^2^[^3^/_2_] | 2 | 1/2)4p2[3/2]2 | 3*s*^2^3*p*^5^(^2^P°_3/2_)4*s* | ^2^[^3^/_2_]° | 1 | 3/2)4s2[3/2]o1 | 738.3980 | IR |
| 3*s*^2^3*p*^5^(^2^P°_3/2_)6*s* | ^2^[^3^/_2_]° | 1 | 3/2)6s2[3/2]o1 | 3*s*^2^3*p*^5^(^2^P°_3/2_)4*p* | ^2^[^3^/_2_] | 2 | 3/2)4p2[3/2]2 | 739.2980 | IR |
| 3*s*^2^3*p*^5^(^2^P°_1/2_)4*d* | ^2^[^5^/_2_]° | 2 | 1/2)4d2[5/2]o2 | 3*s*^2^3*p*^5^(^2^P°_1/2_)4*p* | ^2^[^3^/_2_] | 1 | 1/2)4p2[3/2]1 | 741.2337 | IR |
| 3*s*^2^3*p*^5^(^2^P°_1/2_)4*d* | ^2^[^3^/_2_]° | 2 | 1/2)4d2[3/2]o2 | 3*s*^2^3*p*^5^(^2^P°_1/2_)4*p* | ^2^[^3^/_2_] | 1 | 1/2)4p2[3/2]1 | 742.2312 | IR |
| 3*s*^2^3*p*^5^(^2^P°_1/2_)4*d* | ^2^[^5^/_2_]° | 3 | 1/2)4d2[5/2]o3 | 3*s*^2^3*p*^5^(^2^P°_1/2_)4*p* | ^2^[^3^/_2_] | 2 | 1/2)4p2[3/2]2 | 742.5294 | IR |
| 3*s*^2^3*p*^5^(^2^P°_3/2_)6*s* | ^2^[^3^/_2_]° | 2 | 3/2)6s2[3/2]o2 | 3*s*^2^3*p*^5^(^2^P°_3/2_)4*p* | ^2^[^3^/_2_] | 2 | 3/2)4p2[3/2]2 | 743.5368 | IR |
| 3*s*^2^3*p*^5^(^2^P°_3/2_)4*d* | ^2^[^3^/_2_]° | 2 | 3/2)4d2[3/2]o2 | 3*s*^2^3*p*^5^(^2^P°_3/2_)4*p* | ^2^[^5^/_2_] | 3 | 3/2)4p2[5/2]3 | 743.6297 | IR |
| 3*s*^2^3*p*^5^(^2^P°_1/2_)4*p* | ^2^[^3^/_2_] | 1 | 1/2)4p2[3/2]1 | 3*s*^2^3*p*^5^(^2^P°_3/2_)4*s* | ^2^[^3^/_2_]° | 1 | 3/2)4s2[3/2]o1 | 747.1164 | IR |
| 3*s*^2^3*p*^5^(^2^P°_3/2_)4*d* | ^2^[^5^/_2_]° | 2 | 3/2)4d2[5/2]o2 | 3*s*^2^3*p*^5^(^2^P°_3/2_)4*p* | ^2^[^3^/_2_] | 1 | 3/2)4p2[3/2]1 | 748.433 | IR |
| 3*s*^2^3*p*^5^(^2^P°_1/2_)4*p* | ^2^[^1^/_2_] | 0 | 1/2)4p2[1/2]0 | 3*s*^2^3*p*^5^(^2^P°_1/2_)4*s* | ^2^[^1^/_2_]° | 1 | 1/2)4s2[1/2]o1 | 750.3868 | IR |
| 3*s*^2^3*p*^5^(^2^P°_1/2_)4*d* | ^2^[^3^/_2_]° | 2 | 1/2)4d2[3/2]o2 | 3*s*^2^3*p*^5^(^2^P°_1/2_)4*p* | ^2^[^3^/_2_] | 2 | 1/2)4p2[3/2]2 | 751.0408 | IR |
| 3*s*^2^3*p*^5^(^2^P°_3/2_)4*p* | ^2^[^1^/_2_] | 0 | 3/2)4p2[1/2]0 | 3*s*^2^3*p*^5^(^2^P°_3/2_)4*s* | ^2^[^3^/_2_]° | 1 | 3/2)4s2[3/2]o1 | 751.4651 | IR |
| 3*s*^2^3*p*^5^(^2^P°_1/2_)4*d* | ^2^[^5^/_2_]° | 2 | 1/2)4d2[5/2]o2 | 3*s*^2^3*p*^5^(^2^P°_1/2_)4*p* | ^2^[^1^/_2_] | 1 | 1/2)4p2[1/2]1 | 761.8344 | IR |
| 3*s*^2^3*p*^5^(^2^P°_1/2_)4*d* | ^2^[^3^/_2_]° | 2 | 1/2)4d2[3/2]o2 | 3*s*^2^3*p*^5^(^2^P°_1/2_)4*p* | ^2^[^1^/_2_] | 1 | 1/2)4p2[1/2]1 | 762.8882 | IR |
| 3*s*^2^3*p*^5^(^2^P°_3/2_)4*p* | ^2^[^3^/_2_] | 2 | 3/2)4p2[3/2]2 | 3*s*^2^3*p*^5^(^2^P°_3/2_)4*s* | ^2^[^3^/_2_]° | 2 | 3/2)4s2[3/2]o2 | 763.5105 | IR |
| 3*s*^2^3*p*^5^(^2^P°_3/2_)4*d* | ^2^[^1^/_2_]° | 1 | 3/2)4d2[1/2]o1 | 3*s*^2^3*p*^5^(^2^P°_3/2_)4*p* | ^2^[^5^/_2_] | 2 | 3/2)4p2[5/2]2 | 767.0057 | IR |
| 3*s*^2^3*p*^5^(^2^P°_3/2_)4*d* | ^2^[^7^/_2_]° | 3 | 3/2)4d2[7/2]o3 | 3*s*^2^3*p*^5^(^2^P°_3/2_)4*p* | ^2^[^3^/_2_] | 2 | 3/2)4p2[3/2]2 | 770.482 | IR |
| 3*s*^2^3*p*^5^(^2^P°_3/2_)4*p* | ^2^[^3^/_2_] | 1 | 3/2)4p2[3/2]1 | 3*s*^2^3*p*^5^(^2^P°_3/2_)4*s* | ^2^[^3^/_2_]° | 2 | 3/2)4s2[3/2]o2 | 772.3760 | IR |
| 3*s*^2^3*p*^5^(^2^P°_1/2_)4*p* | ^2^[^1^/_2_] | 1 | 1/2)4p2[1/2]1 | 3*s*^2^3*p*^5^(^2^P°_1/2_)4*s* | ^2^[^1^/_2_]° | 0 | 1/2)4s2[1/2]o0 | 772.4207 | IR |
| 3*s*^2^3*p*^5^(^2^P°_3/2_)4*d* | ^2^[^3^/_2_]° | 2 | 3/2)4d2[3/2]o2 | 3*s*^2^3*p*^5^(^2^P°_3/2_)4*p* | ^2^[^3^/_2_] | 1 | 3/2)4p2[3/2]1 | 779.8561 | IR |
| 3*s*^2^3*p*^5^(^2^P°_3/2_)6*s* | ^2^[^3^/_2_]° | 1 | 3/2)6s2[3/2]o1 | 3*s*^2^3*p*^5^(^2^P°_3/2_)4*p* | ^2^[^1^/_2_] | 0 | 3/2)4p2[1/2]0 | 786.8194 | IR |
| 3*s*^2^3*p*^5^(^2^P°_3/2_)4*d* | ^2^[^3^/_2_]° | 2 | 3/2)4d2[3/2]o2 | 3*s*^2^3*p*^5^(^2^P°_3/2_)4*p* | ^2^[^3^/_2_] | 2 | 3/2)4p2[3/2]2 | 789.1075 | IR |
| 3*s*^2^3*p*^5^(^2^P°_3/2_)6*s* | ^2^[^3^/_2_]° | 1 | 3/2)6s2[3/2]o1 | 3*s*^2^3*p*^5^(^2^P°_1/2_)4*p* | ^2^[^3^/_2_] | 1 | 1/2)4p2[3/2]1 | 791.6442 | IR |
| 3*s*^2^3*p*^5^(^2^P°_1/2_)4*p* | ^2^[^3^/_2_] | 1 | 1/2)4p2[3/2]1 | 3*s*^2^3*p*^5^(^2^P°_1/2_)4*s* | ^2^[^1^/_2_]° | 0 | 1/2)4s2[1/2]o0 | 794.8176 | IR |
| 3*s*^2^3*p*^5^(^2^P°_3/2_)6*s* | ^2^[^3^/_2_]° | 2 | 3/2)6s2[3/2]o2 | 3*s*^2^3*p*^5^(^2^P°_1/2_)4*p* | ^2^[^3^/_2_] | 1 | 1/2)4p2[3/2]1 | 796.5065 | IR |
| 3*s*^2^3*p*^5^(^2^P°_3/2_)4*p* | ^2^[^3^/_2_] | 2 | 3/2)4p2[3/2]2 | 3*s*^2^3*p*^5^(^2^P°_3/2_)4*s* | ^2^[^3^/_2_]° | 1 | 3/2)4s2[3/2]o1 | 800.6156 | IR |
| 3*s*^2^3*p*^5^(^2^P°_3/2_)4*p* | ^2^[^5^/_2_] | 2 | 3/2)4p2[5/2]2 | 3*s*^2^3*p*^5^(^2^P°_3/2_)4*s* | ^2^[^3^/_2_]° | 2 | 3/2)4s2[3/2]o2 | 801.4785 | IR |
| 3*s*^2^3*p*^5^(^2^P°_3/2_)6*s* | ^2^[^3^/_2_]° | 1 | 3/2)6s2[3/2]o1 | 3*s*^2^3*p*^5^(^2^P°_1/2_)4*p* | ^2^[^3^/_2_] | 2 | 1/2)4p2[3/2]2 | 801.6738 | IR |
| 3*s*^2^3*p*^5^(^2^P°_1/2_)6*s* | ^2^[^1^/_2_]° | 1 | 1/2)6s2[1/2]o1 | 3*s*^2^3*p*^5^(^2^P°_1/2_)4*p* | ^2^[^1^/_2_] | 0 | 1/2)4p2[1/2]0 | 803.7219 | IR |
| 3*s*^2^3*p*^5^(^2^P°_3/2_)4*d* | ^2^[^1^/_2_]° | 0 | 3/2)4d2[1/2]o0 | 3*s*^2^3*p*^5^(^2^P°_3/2_)4*p* | ^2^[^3^/_2_] | 1 | 3/2)4p2[3/2]1 | 804.6118 | IR |
| 3*s*^2^3*p*^5^(^2^P°_3/2_)4*d* | ^2^[^1^/_2_]° | 1 | 3/2)4d2[1/2]o1 | 3*s*^2^3*p*^5^(^2^P°_3/2_)4*p* | ^2^[^3^/_2_] | 2 | 3/2)4p2[3/2]2 | 805.3308 | IR |
| 3*s*^2^3*p*^5^(^2^P°_3/2_)6*s* | ^2^[^3^/_2_]° | 2 | 3/2)6s2[3/2]o2 | 3*s*^2^3*p*^5^(^2^P°_1/2_)4*p* | ^2^[^3^/_2_] | 2 | 1/2)4p2[3/2]2 | 806.6604 | IR |
| 3*s*^2^3*p*^5^(^2^P°_3/2_)4*p* | ^2^[^3^/_2_] | 1 | 3/2)4p2[3/2]1 | 3*s*^2^3*p*^5^(^2^P°_3/2_)4*s* | ^2^[^3^/_2_]° | 1 | 3/2)4s2[3/2]o1 | 810.3692 | IR |
| 3*s*^2^3*p*^5^(^2^P°_3/2_)4*p* | ^2^[^5^/_2_] | 3 | 3/2)4p2[5/2]3 | 3*s*^2^3*p*^5^(^2^P°_3/2_)4*s* | ^2^[^3^/_2_]° | 2 | 3/2)4s2[3/2]o2 | 811.5311 | IR |
| 3*s*^2^3*p*^5^(^2^P°_3/2_)6*s* | ^2^[^3^/_2_]° | 1 | 3/2)6s2[3/2]o1 | 3*s*^2^3*p*^5^(^2^P°_1/2_)4*p* | ^2^[^1^/_2_] | 1 | 1/2)4p2[1/2]1 | 815.1868 | IR |
| 3*s*^2^3*p*^5^(^2^P°_3/2_)6*s* | ^2^[^3^/_2_]° | 2 | 3/2)6s2[3/2]o2 | 3*s*^2^3*p*^5^(^2^P°_1/2_)4*p* | ^2^[^1^/_2_] | 1 | 1/2)4p2[1/2]1 | 820.3435 | IR |
| 3*s*^2^3*p*^5^(^2^P°_1/2_)4*p* | ^2^[^1^/_2_] | 1 | 1/2)4p2[1/2]1 | 3*s*^2^3*p*^5^(^2^P°_1/2_)4*s* | ^2^[^1^/_2_]° | 1 | 1/2)4s2[1/2]o1 | 826.4521 | IR |
| 3*s*^2^3*p*^5^(^2^P°_3/2_)4*d* | ^2^[^7^/_2_]° | 3 | 3/2)4d2[7/2]o3 | 3*s*^2^3*p*^5^(^2^P°_1/2_)4*p* | ^2^[^3^/_2_] | 2 | 1/2)4p2[3/2]2 | 838.473 | IR |
| 3*s*^2^3*p*^5^(^2^P°_1/2_)4*p* | ^2^[^3^/_2_] | 2 | 1/2)4p2[3/2]2 | 3*s*^2^3*p*^5^(^2^P°_1/2_)4*s* | ^2^[^1^/_2_]° | 1 | 1/2)4s2[1/2]o1 | 840.8209 | IR |
| 3*s*^2^3*p*^5^(^2^P°_3/2_)4*p* | ^2^[^5^/_2_] | 2 | 3/2)4p2[5/2]2 | 3*s*^2^3*p*^5^(^2^P°_3/2_)4*s* | ^2^[^3^/_2_]° | 1 | 3/2)4s2[3/2]o1 | 842.4647 | IR |
| 3*s*^2^3*p*^5^(^2^P°_3/2_)4*d* | ^2^[^3^/_2_]° | 2 | 3/2)4d2[3/2]o2 | 3*s*^2^3*p*^5^(^2^P°_1/2_)4*p* | ^2^[^3^/_2_] | 1 | 1/2)4p2[3/2]1 | 849.0307 | IR |
| 3*s*^2^3*p*^5^(^2^P°_1/2_)4*p* | ^2^[^3^/_2_] | 1 | 1/2)4p2[3/2]1 | 3*s*^2^3*p*^5^(^2^P°_1/2_)4*s* | ^2^[^1^/_2_]° | 1 | 1/2)4s2[1/2]o1 | 852.1441 | IR |
| 3*s*^2^3*p*^5^(^2^P°_3/2_)4*d* | ^2^[^3^/_2_]° | 2 | 3/2)4d2[3/2]o2 | 3*s*^2^3*p*^5^(^2^P°_1/2_)4*p* | ^2^[^3^/_2_] | 2 | 1/2)4p2[3/2]2 | 860.5776 | IR |
| 3*s*^2^3*p*^5^(^2^P°_3/2_)4*d* | ^2^[^1^/_2_]° | 1 | 3/2)4d2[1/2]o1 | 3*s*^2^3*p*^5^(^2^P°_3/2_)4*p* | ^2^[^1^/_2_] | 0 | 3/2)4p2[1/2]0 | 862.0460 | IR |
| 3*s*^2^3*p*^5^(^2^P°_3/2_)4*p* | ^2^[^3^/_2_] | 1 | 3/2)4p2[3/2]1 | 3*s*^2^3*p*^5^(^2^P°_1/2_)4*s* | ^2^[^1^/_2_]° | 0 | 1/2)4s2[1/2]o0 | 866.7943 | IR |
| 3*s*^2^3*p*^5^(^2^P°_3/2_)4*d* | ^2^[^3^/_2_]° | 2 | 3/2)4d2[3/2]o2 | 3*s*^2^3*p*^5^(^2^P°_1/2_)4*p* | ^2^[^1^/_2_] | 1 | 1/2)4p2[1/2]1 | 876.1686 | IR |
| 3*s*^2^3*p*^5^(^2^P°_3/2_)4*d* | ^2^[^1^/_2_]° | 0 | 3/2)4d2[1/2]o0 | 3*s*^2^3*p*^5^(^2^P°_1/2_)4*p* | ^2^[^3^/_2_] | 1 | 1/2)4p2[3/2]1 | 878.4557 | IR |
| 3*s*^2^3*p*^5^(^2^P°_3/2_)4*d* | ^2^[^1^/_2_]° | 1 | 3/2)4d2[1/2]o1 | 3*s*^2^3*p*^5^(^2^P°_1/2_)4*p* | ^2^[^3^/_2_] | 2 | 1/2)4p2[3/2]2 | 879.9087 | IR |
| 3*s*^2^3*p*^5^(^2^P°_3/2_)4*d* | ^2^[^1^/_2_]° | 1 | 3/2)4d2[1/2]o1 | 3*s*^2^3*p*^5^(^2^P°_1/2_)4*p* | ^2^[^1^/_2_] | 1 | 1/2)4p2[1/2]1 | 896.2147 | IR |
| 3*s*^2^3*p*^5^(^2^P°_3/2_)6*s* | ^2^[^3^/_2_]° | 1 | 3/2)6s2[3/2]o1 | 3*s*^2^3*p*^5^(^2^P°_1/2_)4*p* | ^2^[^1^/_2_] | 0 | 1/2)4p2[1/2]0 | 905.7493 | IR |
| 3*s*^2^3*p*^5^(^2^P°_3/2_)4*d* | ^2^[^1^/_2_]° | 0 | 3/2)4d2[1/2]o0 | 3*s*^2^3*p*^5^(^2^P°_1/2_)4*p* | ^2^[^1^/_2_] | 1 | 1/2)4p2[1/2]1 | 907.5395 | IR |
| 3*s*^2^3*p*^5^(^2^P°_3/2_)4*p* | ^2^[^1^/_2_] | 1 | 3/2)4p2[1/2]1 | 3*s*^2^3*p*^5^(^2^P°_3/2_)4*s* | ^2^[^3^/_2_]° | 2 | 3/2)4s2[3/2]o2 | 912.2967 | IR |
| 3*s*^2^3*p*^5^(^2^P°_1/2_)5*s* | ^2^[^1^/_2_]° | 1 | 1/2)5s2[1/2]o1 | 3*s*^2^3*p*^5^(^2^P°_3/2_)4*p* | ^2^[^1^/_2_] | 1 | 3/2)4p2[1/2]1 | 919.4638 | IR |
| 3*s*^2^3*p*^5^(^2^P°_3/2_)4*p* | ^2^[^3^/_2_] | 2 | 3/2)4p2[3/2]2 | 3*s*^2^3*p*^5^(^2^P°_1/2_)4*s* | ^2^[^1^/_2_]° | 1 | 1/2)4s2[1/2]o1 | 922.4498 | IR |
| 3*s*^2^3*p*^5^(^2^P°_1/2_)5*s* | ^2^[^1^/_2_]° | 0 | 1/2)5s2[1/2]o0 | 3*s*^2^3*p*^5^(^2^P°_3/2_)4*p* | ^2^[^1^/_2_] | 1 | 3/2)4p2[1/2]1 | 929.1532 | IR |
| 3*s*^2^3*p*^5^(^2^P°_3/2_)4*p* | ^2^[^3^/_2_] | 1 | 3/2)4p2[3/2]1 | 3*s*^2^3*p*^5^(^2^P°_1/2_)4*s* | ^2^[^1^/_2_]° | 1 | 1/2)4s2[1/2]o1 | 935.4218 | IR |
| 3*s*^2^3*p*^5^(^2^P°_3/2_)4*p* | ^2^[^1^/_2_] | 1 | 3/2)4p2[1/2]1 | 3*s*^2^3*p*^5^(^2^P°_3/2_)4*s* | ^2^[^3^/_2_]° | 1 | 3/2)4s2[3/2]o1 | 965.7786 | IR |
| 3*s*^2^3*p*^5^(^2^P°_3/2_)4*p* | ^2^[^5^/_2_] | 2 | 3/2)4p2[5/2]2 | 3*s*^2^3*p*^5^(^2^P°_1/2_)4*s* | ^2^[^1^/_2_]° | 1 | 1/2)4s2[1/2]o1 | 978.4502 | IR |
| 3*s*^2^3*p*^5^(^2^P°_3/2_)4*p* | ^2^[^1^/_2_] | 1 | 3/2)4p2[1/2]1 | 3*s*^2^3*p*^5^(^2^P°_1/2_)4*s* | ^2^[^1^/_2_]° | 0 | 1/2)4s2[1/2]o0 | 1047.0053 | IR |
| 3*s*^2^3*p*^5^(^2^P°_3/2_)5*s* | ^2^[^3^/_2_]° | 1 | 3/2)5s2[3/2]o1 | 3*s*^2^3*p*^5^(^2^P°_3/2_)4*p* | ^2^[^1^/_2_] | 1 | 3/2)4p2[1/2]1 | 1047.8034 | IR |
| 3*s*^2^3*p*^5^(^2^P°_1/2_)4*f* | ^2^[^5^/_2_] | 2 | 1/2)4f2[5/2]2 | 3*s*^2^3*p*^5^(^2^P°_3/2_)3*d* | ^2^[^3^/_2_]° | 2 | 3/2)3d2[3/2]o2 | 1050.6118 | IR |
| 3*s*^2^3*p*^5^(^2^P°_1/2_)4*f* | ^2^[^5^/_2_] | 3 | 1/2)4f2[5/2]3 | 3*s*^2^3*p*^5^(^2^P°_3/2_)3*d* | ^2^[^3^/_2_]° | 2 | 3/2)3d2[3/2]o2 | 1050.6500 | IR |
| 3*s*^2^3*p*^5^(^2^P°_3/2_)5*s* | ^2^[^3^/_2_]° | 2 | 3/2)5s2[3/2]o2 | 3*s*^2^3*p*^5^(^2^P°_3/2_)4*p* | ^2^[^1^/_2_] | 1 | 3/2)4p2[1/2]1 | 1067.3566 | IR |
| 3*s*^2^3*p*^5^(^2^P°_1/2_)5*s* | ^2^[^1^/_2_]° | 1 | 1/2)5s2[1/2]o1 | 3*s*^2^3*p*^5^(^2^P°_3/2_)4*p* | ^2^[^5^/_2_] | 2 | 3/2)4p2[5/2]2 | 1068.3404 | IR |
| 3*s*^2^3*p*^5^(^2^P°_1/2_)3*d* | ^2^[^3^/_2_]° | 1 | 1/2)3d2[3/2]o1 | 3*s*^2^3*p*^5^(^2^P°_3/2_)4*p* | ^2^[^3^/_2_] | 2 | 3/2)4p2[3/2]2 | 1095.0726 | IR |
| 3*s*^2^3*p*^5^(^2^P°_1/2_)3*d* | ^2^[^5^/_2_]° | 2 | 1/2)3d2[5/2]o2 | 3*s*^2^3*p*^5^(^2^P°_3/2_)4*p* | ^2^[^5^/_2_] | 2 | 3/2)4p2[5/2]2 | 1107.8868 | IR |
| 3*s*^2^3*p*^5^(^2^P°_1/2_)5*s* | ^2^[^1^/_2_]° | 1 | 1/2)5s2[1/2]o1 | 3*s*^2^3*p*^5^(^2^P°_3/2_)4*p* | ^2^[^3^/_2_] | 1 | 3/2)4p2[3/2]1 | 1124.8350 | IR |
| 3*s*^2^3*p*^5^(^2^P°_1/2_)5*s* | ^2^[^1^/_2_]° | 0 | 1/2)5s2[1/2]o0 | 3*s*^2^3*p*^5^(^2^P°_3/2_)4*p* | ^2^[^3^/_2_] | 1 | 3/2)4p2[3/2]1 | 1139.3703 | IR |
| 3*s*^2^3*p*^5^(^2^P°_1/2_)5*s* | ^2^[^1^/_2_]° | 1 | 1/2)5s2[1/2]o1 | 3*s*^2^3*p*^5^(^2^P°_3/2_)4*p* | ^2^[^3^/_2_] | 2 | 3/2)4p2[3/2]2 | 1144.1832 | IR |
| 3*s*^2^3*p*^5^(^2^P°_1/2_)3*d* | ^2^[^3^/_2_]° | 2 | 1/2)3d2[3/2]o2 | 3*s*^2^3*p*^5^(^2^P°_3/2_)4*p* | ^2^[^3^/_2_] | 1 | 3/2)4p2[3/2]1 | 1146.7545 | IR |
| 3*s*^2^3*p*^5^(^2^P°_3/2_)4*p* | ^2^[^1^/_2_] | 1 | 3/2)4p2[1/2]1 | 3*s*^2^3*p*^5^(^2^P°_1/2_)4*s* | ^2^[^1^/_2_]° | 1 | 1/2)4s2[1/2]o1 | 1148.8108 | IR |
| 3*s*^2^3*p*^5^(^2^P°_1/2_)4*f* | ^2^[^7^/_2_] | 4 | 1/2)4f2[7/2]4 | 3*s*^2^3*p*^5^(^2^P°_3/2_)3*d* | ^2^[^7^/_2_]° | 3 | 3/2)3d2[7/2]o3 | 1158.0389 | IR |
| 3*s*^2^3*p*^5^(^2^P°_1/2_)4*f* | ^2^[^7^/_2_] | 3 | 1/2)4f2[7/2]3 | 3*s*^2^3*p*^5^(^2^P°_3/2_)3*d* | ^2^[^7^/_2_]° | 3 | 3/2)3d2[7/2]o3 | 1158.0446 | IR |
| 3*s*^2^3*p*^5^(^2^P°_1/2_)3*d* | ^2^[^3^/_2_]° | 2 | 1/2)3d2[3/2]o2 | 3*s*^2^3*p*^5^(^2^P°_3/2_)4*p* | ^2^[^3^/_2_] | 2 | 3/2)4p2[3/2]2 | 1166.8709 | IR |
| 3*s*^2^3*p*^5^(^2^P°_3/2_)3*d* | ^2^[^3^/_2_]° | 1 | 3/2)3d2[3/2]o1 | 3*s*^2^3*p*^5^(^2^P°_3/2_)4*p* | ^2^[^5^/_2_] | 2 | 3/2)4p2[5/2]2 | 1171.9487 | IR |
| 3*s*^2^3*p*^5^(^2^P°_3/2_)4*f* | ^2^[^3^/_2_] | 1 | 3/2)4f2[3/2]1 | 3*s*^2^3*p*^5^(^2^P°_3/2_)3*d* | ^2^[^1^/_2_]° | 0 | 3/2)3d2[1/2]o0 | 1173.3236 | IR |
| 3*s*^2^3*p*^5^(^2^P°_3/2_)4*f* | ^2^[^3^/_2_] | 2 | 3/2)4f2[3/2]2 | 3*s*^2^3*p*^5^(^2^P°_3/2_)3*d* | ^2^[^1^/_2_]° | 1 | 3/2)3d2[1/2]o1 | 1194.3302 | IR |
| 3*s*^2^3*p*^5^(^2^P°_3/2_)4*f* | ^2^[^3^/_2_] | 1 | 3/2)4f2[3/2]1 | 3*s*^2^3*p*^5^(^2^P°_3/2_)3*d* | ^2^[^1^/_2_]° | 1 | 3/2)3d2[1/2]o1 | 1194.3871 | IR |
| 3*s*^2^3*p*^5^(^2^P°_1/2_)3*d* | ^2^[^3^/_2_]° | 1 | 1/2)3d2[3/2]o1 | 3*s*^2^3*p*^5^(^2^P°_3/2_)4*p* | ^2^[^1^/_2_] | 0 | 3/2)4p2[1/2]0 | 1202.6648 | IR |
| 3*s*^2^3*p*^5^(^2^P°_3/2_)3*d* | ^2^[^5^/_2_]° | 3 | 3/2)3d2[5/2]o3 | 3*s*^2^3*p*^5^(^2^P°_3/2_)4*p* | ^2^[^5^/_2_] | 3 | 3/2)4p2[5/2]3 | 1211.2324 | IR |
| 3*s*^2^3*p*^5^(^2^P°_1/2_)3*d* | ^2^[^3^/_2_]° | 1 | 1/2)3d2[3/2]o1 | 3*s*^2^3*p*^5^(^2^P°_1/2_)4*p* | ^2^[^3^/_2_] | 1 | 1/2)4p2[3/2]1 | 1213.9737 | IR |
| 3*s*^2^3*p*^5^(^2^P°_3/2_)3*d* | ^2^[^5^/_2_]° | 3 | 3/2)3d2[5/2]o3 | 3*s*^2^3*p*^5^(^2^P°_3/2_)4*p* | ^2^[^5^/_2_] | 2 | 3/2)4p2[5/2]2 | 1234.3392 | IR |
| 3*s*^2^3*p*^5^(^2^P°_3/2_)4*f* | ^2^[^5^/_2_] | 2 | 3/2)4f2[5/2]2 | 3*s*^2^3*p*^5^(^2^P°_3/2_)3*d* | ^2^[^3^/_2_]° | 2 | 3/2)3d2[3/2]o2 | 1235.5819 | IR |
| 3*s*^2^3*p*^5^(^2^P°_3/2_)4*f* | ^2^[^5^/_2_] | 3 | 3/2)4f2[5/2]3 | 3*s*^2^3*p*^5^(^2^P°_3/2_)3*d* | ^2^[^3^/_2_]° | 2 | 3/2)3d2[3/2]o2 | 1235.6297 | IR |
| 3*s*^2^3*p*^5^(^2^P°_3/2_)3*d* | ^2^[^3^/_2_]° | 1 | 3/2)3d2[3/2]o1 | 3*s*^2^3*p*^5^(^2^P°_3/2_)4*p* | ^2^[^3^/_2_] | 1 | 3/2)4p2[3/2]1 | 1240.2828 | IR |
| 3*s*^2^3*p*^5^(^2^P°_3/2_)3*d* | ^2^[^3^/_2_]° | 2 | 3/2)3d2[3/2]o2 | 3*s*^2^3*p*^5^(^2^P°_3/2_)4*p* | ^2^[^1^/_2_] | 1 | 3/2)4p2[1/2]1 | 1243.9321 | IR |
| 3*s*^2^3*p*^5^(^2^P°_3/2_)5*s* | ^2^[^3^/_2_]° | 1 | 3/2)5s2[3/2]o1 | 3*s*^2^3*p*^5^(^2^P°_3/2_)4*p* | ^2^[^5^/_2_] | 2 | 3/2)4p2[5/2]2 | 1245.6114 | IR |
| 3*s*^2^3*p*^5^(^2^P°_3/2_)5*s* | ^2^[^3^/_2_]° | 2 | 3/2)5s2[3/2]o2 | 3*s*^2^3*p*^5^(^2^P°_3/2_)4*p* | ^2^[^5^/_2_] | 3 | 3/2)4p2[5/2]3 | 1248.7663 | IR |
| 3*s*^2^3*p*^5^(^2^P°_3/2_)3*d* | ^2^[^5^/_2_]° | 2 | 3/2)3d2[5/2]o2 | 3*s*^2^3*p*^5^(^2^P°_3/2_)4*p* | ^2^[^5^/_2_] | 3 | 3/2)4p2[5/2]3 | 1255.4324 | IR |
| 3*s*^2^3*p*^5^(^2^P°_1/2_)5*s* | ^2^[^1^/_2_]° | 1 | 1/2)5s2[1/2]o1 | 3*s*^2^3*p*^5^(^2^P°_3/2_)4*p* | ^2^[^1^/_2_] | 0 | 3/2)4p2[1/2]0 | 1262.1619 | IR |
| 3*s*^2^3*p*^5^(^2^P°_1/2_)3*d* | ^2^[^3^/_2_]° | 1 | 1/2)3d2[3/2]o1 | 3*s*^2^3*p*^5^(^2^P°_1/2_)4*p* | ^2^[^1^/_2_] | 1 | 1/2)4p2[1/2]1 | 1270.2280 | IR |
| 3*s*^2^3*p*^5^(^2^P°_3/2_)5*s* | ^2^[^3^/_2_]° | 2 | 3/2)5s2[3/2]o2 | 3*s*^2^3*p*^5^(^2^P°_3/2_)4*p* | ^2^[^5^/_2_] | 2 | 3/2)4p2[5/2]2 | 1273.3418 | IR |
| 3*s*^2^3*p*^5^(^2^P°_1/2_)5*s* | ^2^[^1^/_2_]° | 1 | 1/2)5s2[1/2]o1 | 3*s*^2^3*p*^5^(^2^P°_1/2_)4*p* | ^2^[^3^/_2_] | 1 | 1/2)4p2[3/2]1 | 1274.6232 | IR |
| 3*s*^2^3*p*^5^(^2^P°_3/2_)3*d* | ^2^[^5^/_2_]° | 2 | 3/2)3d2[5/2]o2 | 3*s*^2^3*p*^5^(^2^P°_3/2_)4*p* | ^2^[^5^/_2_] | 2 | 3/2)4p2[5/2]2 | 1280.2737 | IR |
| 3*s*^2^3*p*^5^(^2^P°_1/2_)5*s* | ^2^[^1^/_2_]° | 0 | 1/2)5s2[1/2]o0 | 3*s*^2^3*p*^5^(^2^P°_1/2_)4*p* | ^2^[^3^/_2_] | 1 | 1/2)4p2[3/2]1 | 1293.3196 | IR |
| 3*s*^2^3*p*^5^(^2^P°_3/2_)3*d* | ^2^[^1^/_2_]° | 1 | 3/2)3d2[1/2]o1 | 3*s*^2^3*p*^5^(^2^P°_3/2_)4*p* | ^2^[^1^/_2_] | 1 | 3/2)4p2[1/2]1 | 1295.6658 | IR |
| 3*s*^2^3*p*^5^(^2^P°_1/2_)5*s* | ^2^[^1^/_2_]° | 1 | 1/2)5s2[1/2]o1 | 3*s*^2^3*p*^5^(^2^P°_1/2_)4*p* | ^2^[^3^/_2_] | 2 | 1/2)4p2[3/2]2 | 1300.8264 | IR |
| 3*s*^2^3*p*^5^(^2^P°_3/2_)3*d* | ^2^[^1^/_2_]° | 0 | 3/2)3d2[1/2]o0 | 3*s*^2^3*p*^5^(^2^P°_3/2_)4*p* | ^2^[^1^/_2_] | 1 | 3/2)4p2[1/2]1 | 1321.3991 | IR |
| 3*s*^2^3*p*^5^(^2^P°_3/2_)5*s* | ^2^[^3^/_2_]° | 1 | 3/2)5s2[3/2]o1 | 3*s*^2^3*p*^5^(^2^P°_3/2_)4*p* | ^2^[^3^/_2_] | 1 | 3/2)4p2[3/2]1 | 1323.0897 | IR |
| 3*s*^2^3*p*^5^(^2^P°_1/2_)3*d* | ^2^[^5^/_2_]° | 3 | 1/2)3d2[5/2]o3 | 3*s*^2^3*p*^5^(^2^P°_1/2_)4*p* | ^2^[^3^/_2_] | 2 | 1/2)4p2[3/2]2 | 1327.2635 | IR |
| 3*s*^2^3*p*^5^(^2^P°_1/2_)3*d* | ^2^[^5^/_2_]° | 2 | 1/2)3d2[5/2]o2 | 3*s*^2^3*p*^5^(^2^P°_1/2_)4*p* | ^2^[^3^/_2_] | 1 | 1/2)4p2[3/2]1 | 1331.3209 | IR |
| 3*s*^2^3*p*^5^(^2^P°_1/2_)5*s* | ^2^[^1^/_2_]° | 1 | 1/2)5s2[1/2]o1 | 3*s*^2^3*p*^5^(^2^P°_1/2_)4*p* | ^2^[^1^/_2_] | 1 | 1/2)4p2[1/2]1 | 1336.7827 | IR |
| 3*s*^2^3*p*^5^(^2^P°_3/2_)4*f* | ^2^[^9^/_2_] | 4 | 3/2)4f2[9/2]4 | 3*s*^2^3*p*^5^(^2^P°_3/2_)3*d* | ^2^[^7^/_2_]° | 4 | 3/2)3d2[7/2]o4 | 1340.6513 | IR |
| 3*s*^2^3*p*^5^(^2^P°_3/2_)4*f* | ^2^[^9^/_2_] | 5 | 3/2)4f2[9/2]5 | 3*s*^2^3*p*^5^(^2^P°_3/2_)3*d* | ^2^[^7^/_2_]° | 4 | 3/2)3d2[7/2]o4 | 1340.6587 | IR |
| 3*s*^2^3*p*^5^(^2^P°_3/2_)5*s* | ^2^[^3^/_2_]° | 1 | 3/2)5s2[3/2]o1 | 3*s*^2^3*p*^5^(^2^P°_3/2_)4*p* | ^2^[^3^/_2_] | 2 | 3/2)4p2[3/2]2 | 1349.9406 | IR |
| 3*s*^2^3*p*^5^(^2^P°_3/2_)3*d* | ^2^[^7^/_2_]° | 3 | 3/2)3d2[7/2]o3 | 3*s*^2^3*p*^5^(^2^P°_3/2_)4*p* | ^2^[^5^/_2_] | 2 | 3/2)4p2[5/2]2 | 1350.4189 | IR |
| 3*s*^2^3*p*^5^(^2^P°_3/2_)5*s* | ^2^[^3^/_2_]° | 2 | 3/2)5s2[3/2]o2 | 3*s*^2^3*p*^5^(^2^P°_3/2_)4*p* | ^2^[^3^/_2_] | 1 | 3/2)4p2[3/2]1 | 1354.4205 | IR |
| 3*s*^2^3*p*^5^(^2^P°_1/2_)5*s* | ^2^[^1^/_2_]° | 0 | 1/2)5s2[1/2]o0 | 3*s*^2^3*p*^5^(^2^P°_1/2_)4*p* | ^2^[^1^/_2_] | 1 | 1/2)4p2[1/2]1 | 1357.3618 | IR |
| 3*s*^2^3*p*^5^(^2^P°_1/2_)3*d* | ^2^[^5^/_2_]° | 2 | 1/2)3d2[5/2]o2 | 3*s*^2^3*p*^5^(^2^P°_1/2_)4*p* | ^2^[^3^/_2_] | 2 | 1/2)4p2[3/2]2 | 1359.9333 | IR |
| 3*s*^2^3*p*^5^(^2^P°_3/2_)3*d* | ^2^[^5^/_2_]° | 2 | 3/2)3d2[5/2]o2 | 3*s*^2^3*p*^5^(^2^P°_3/2_)4*p* | ^2^[^3^/_2_] | 1 | 3/2)4p2[3/2]1 | 1362.2659 | IR |
| 3*s*^2^3*p*^5^(^2^P°_1/2_)3*d* | ^2^[^3^/_2_]° | 2 | 1/2)3d2[3/2]o2 | 3*s*^2^3*p*^5^(^2^P°_1/2_)4*p* | ^2^[^1^/_2_] | 1 | 1/2)4p2[1/2]1 | 1367.8549 | IR |
| 3*s*^2^3*p*^5^(^2^P°_3/2_)5*s* | ^2^[^3^/_2_]° | 2 | 3/2)5s2[3/2]o2 | 3*s*^2^3*p*^5^(^2^P°_3/2_)4*p* | ^2^[^3^/_2_] | 2 | 3/2)4p2[3/2]2 | 1382.5716 | IR |
| 3*s*^2^3*p*^5^(^2^P°_3/2_)3*d* | ^2^[^3^/_2_]° | 1 | 3/2)3d2[3/2]o1 | 3*s*^2^3*p*^5^(^2^P°_3/2_)4*p* | ^2^[^1^/_2_] | 0 | 3/2)4p2[1/2]0 | 1409.3640 | IR |
| 3*s*^2^3*p*^5^(^2^P°_1/2_)4*f* | ^2^[^5^/_2_] | 2 | 1/2)4f2[5/2]2 | 3*s*^2^3*p*^5^(^2^P°_1/2_)3*d* | ^2^[^3^/_2_]° | 2 | 1/2)3d2[3/2]o2 | 1459.5734 | IR |
| 3*s*^2^3*p*^5^(^2^P°_1/2_)4*f* | ^2^[^5^/_2_] | 3 | 1/2)4f2[5/2]3 | 3*s*^2^3*p*^5^(^2^P°_1/2_)3*d* | ^2^[^3^/_2_]° | 2 | 1/2)3d2[3/2]o2 | 1459.6471 | IR |
| 3*s*^2^3*p*^5^(^2^P°_1/2_)4*f* | ^2^[^7^/_2_] | 4 | 1/2)4f2[7/2]4 | 3*s*^2^3*p*^5^(^2^P°_1/2_)3*d* | ^2^[^5^/_2_]° | 3 | 1/2)3d2[5/2]o3 | 1463.4414 | IR |
| 3*s*^2^3*p*^5^(^2^P°_1/2_)4*f* | ^2^[^7^/_2_] | 3 | 1/2)4f2[7/2]3 | 3*s*^2^3*p*^5^(^2^P°_1/2_)3*d* | ^2^[^5^/_2_]° | 3 | 1/2)3d2[5/2]o3 | 1463.4504 | IR |
| 3*s*^2^3*p*^5^(^2^P°_3/2_)3*d* | ^2^[^7^/_2_]° | 3 | 3/2)3d2[7/2]o3 | 3*s*^2^3*p*^5^(^2^P°_3/2_)4*p* | ^2^[^3^/_2_] | 2 | 3/2)4p2[3/2]2 | 1473.9139 | IR |
| 3*s*^2^3*p*^5^(^2^P°_3/2_)4*f* | ^2^[^5^/_2_] | 2 | 3/2)4f2[5/2]2 | 3*s*^2^3*p*^5^(^2^P°_3/2_)5*s* | ^2^[^3^/_2_]° | 2 | 3/2)5s2[3/2]o2 | 1478.5380 | IR |
| 3*s*^2^3*p*^5^(^2^P°_3/2_)4*f* | ^2^[^5^/_2_] | 3 | 3/2)4f2[5/2]3 | 3*s*^2^3*p*^5^(^2^P°_3/2_)5*s* | ^2^[^3^/_2_]° | 2 | 3/2)5s2[3/2]o2 | 1478.6064 | IR |
| 3*s*^2^3*p*^5^(^2^P°_3/2_)4*f* | ^2^[^3^/_2_] | 2 | 3/2)4f2[3/2]2 | 3*s*^2^3*p*^5^(^2^P°_3/2_)5*s* | ^2^[^3^/_2_]° | 2 | 3/2)5s2[3/2]o2 | 1487.6537 | IR |
| 3*s*^2^3*p*^5^(^2^P°_3/2_)4*f* | ^2^[^3^/_2_] | 1 | 3/2)4f2[3/2]1 | 3*s*^2^3*p*^5^(^2^P°_3/2_)5*s* | ^2^[^3^/_2_]° | 2 | 3/2)5s2[3/2]o2 | 1487.7420 | IR |
| 3*s*^2^3*p*^5^(^2^P°_1/2_)3*d* | ^2^[^3^/_2_]° | 1 | 1/2)3d2[3/2]o1 | 3*s*^2^3*p*^5^(^2^P°_1/2_)4*p* | ^2^[^1^/_2_] | 0 | 1/2)4p2[1/2]0 | 1504.6503 | IR |
| 3*s*^2^3*p*^5^(^2^P°_3/2_)5*s* | ^2^[^3^/_2_]° | 1 | 3/2)5s2[3/2]o1 | 3*s*^2^3*p*^5^(^2^P°_3/2_)4*p* | ^2^[^1^/_2_] | 0 | 3/2)4p2[1/2]0 | 1517.2691 | IR |
| 3*s*^2^3*p*^5^(^2^P°_3/2_)4*f* | ^2^[^7^/_2_] | 4 | 3/2)4f2[7/2]4 | 3*s*^2^3*p*^5^(^2^P°_3/2_)3*d* | ^2^[^5^/_2_]° | 3 | 3/2)3d2[5/2]o3 | 1530.1881 | IR |
| 3*s*^2^3*p*^5^(^2^P°_3/2_)4*f* | ^2^[^7^/_2_] | 3 | 3/2)4f2[7/2]3 | 3*s*^2^3*p*^5^(^2^P°_3/2_)3*d* | ^2^[^5^/_2_]° | 3 | 3/2)3d2[5/2]o3 | 1530.1970 | IR |
| 3*s*^2^3*p*^5^(^2^P°_3/2_)3*d* | ^2^[^3^/_2_]° | 2 | 3/2)3d2[3/2]o2 | 3*s*^2^3*p*^5^(^2^P°_3/2_)4*p* | ^2^[^5^/_2_] | 2 | 3/2)4p2[5/2]2 | 1532.9345 | IR |
| 3*s*^2^3*p*^5^(^2^P°_3/2_)5*s* | ^2^[^3^/_2_]° | 1 | 3/2)5s2[3/2]o1 | 3*s*^2^3*p*^5^(^2^P°_1/2_)4*p* | ^2^[^3^/_2_] | 1 | 1/2)4p2[3/2]1 | 1535.3128 | IR |
| 3*s*^2^3*p*^5^(^2^P°_3/2_)4*f* | ^2^[^9^/_2_] | 4 | 3/2)4f2[9/2]4 | 3*s*^2^3*p*^5^(^2^P°_3/2_)3*d* | ^2^[^5^/_2_]° | 3 | 3/2)3d2[5/2]o3 | 1540.2640 | IR |
| 3*s*^2^3*p*^5^(^2^P°_3/2_)3*d* | ^2^[^5^/_2_]° | 3 | 3/2)3d2[5/2]o3 | 3*s*^2^3*p*^5^(^2^P°_1/2_)4*p* | ^2^[^3^/_2_] | 2 | 1/2)4p2[3/2]2 | 1555.5460 | IR |
| 3*s*^2^3*p*^5^(^2^P°_3/2_)5*s* | ^2^[^3^/_2_]° | 1 | 3/2)5s2[3/2]o1 | 3*s*^2^3*p*^5^(^2^P°_1/2_)4*p* | ^2^[^3^/_2_] | 2 | 1/2)4p2[3/2]2 | 1573.4909 | IR |
| 3*s*^2^3*p*^5^(^2^P°_3/2_)5*s* | ^2^[^3^/_2_]° | 2 | 3/2)5s2[3/2]o2 | 3*s*^2^3*p*^5^(^2^P°_1/2_)4*p* | ^2^[^3^/_2_] | 1 | 1/2)4p2[3/2]1 | 1577.6614 | IR |
| 3*s*^2^3*p*^5^(^2^P°_1/2_)5*p* | ^2^[^1^/_2_] | 1 | 1/2)5p2[1/2]1 | 3*s*^2^3*p*^5^(^2^P°_3/2_)3*d* | ^2^[^3^/_2_]° | 2 | 3/2)3d2[3/2]o2 | 1581.6777 | IR |
| 3*s*^2^3*p*^5^(^2^P°_1/2_)4*f* | ^2^[^5^/_2_] | 2 | 1/2)4f2[5/2]2 | 3*s*^2^3*p*^5^(^2^P°_1/2_)3*d* | ^2^[^3^/_2_]° | 1 | 1/2)3d2[3/2]o1 | 1589.9687 | IR |
| 3*s*^2^3*p*^5^(^2^P°_1/2_)5*s* | ^2^[^1^/_2_]° | 1 | 1/2)5s2[1/2]o1 | 3*s*^2^3*p*^5^(^2^P°_1/2_)4*p* | ^2^[^1^/_2_] | 0 | 1/2)4p2[1/2]0 | 1598.9491 | IR |
| 3*s*^2^3*p*^5^(^2^P°_3/2_)3*d* | ^2^[^1^/_2_]° | 1 | 3/2)3d2[1/2]o1 | 3*s*^2^3*p*^5^(^2^P°_3/2_)4*p* | ^2^[^5^/_2_] | 2 | 3/2)4p2[5/2]2 | 1612.2656 | IR |
| 3*s*^2^3*p*^5^(^2^P°_3/2_)5*s* | ^2^[^3^/_2_]° | 2 | 3/2)5s2[3/2]o2 | 3*s*^2^3*p*^5^(^2^P°_1/2_)4*p* | ^2^[^3^/_2_] | 2 | 1/2)4p2[3/2]2 | 1618.0023 | IR |
| 3*s*^2^3*p*^5^(^2^P°_3/2_)5*s* | ^2^[^3^/_2_]° | 1 | 3/2)5s2[3/2]o1 | 3*s*^2^3*p*^5^(^2^P°_1/2_)4*p* | ^2^[^1^/_2_] | 1 | 1/2)4p2[1/2]1 | 1626.4070 | IR |
| 3*s*^2^3*p*^5^(^2^P°_3/2_)4*f* | ^2^[^5^/_2_] | 2 | 3/2)4f2[5/2]2 | 3*s*^2^3*p*^5^(^2^P°_3/2_)3*d* | ^2^[^3^/_2_]° | 1 | 3/2)3d2[3/2]o1 | 1643.6575 | IR |
| 3*s*^2^3*p*^5^(^2^P°_3/2_)3*d* | ^2^[^3^/_2_]° | 2 | 3/2)3d2[3/2]o2 | 3*s*^2^3*p*^5^(^2^P°_3/2_)4*p* | ^2^[^3^/_2_] | 1 | 3/2)4p2[3/2]1 | 1651.9867 | IR |
| 3*s*^2^3*p*^5^(^2^P°_3/2_)4*f* | ^2^[^3^/_2_] | 2 | 3/2)4f2[3/2]2 | 3*s*^2^3*p*^5^(^2^P°_3/2_)3*d* | ^2^[^3^/_2_]° | 1 | 3/2)3d2[3/2]o1 | 1654.9307 | IR |
| 3*s*^2^3*p*^5^(^2^P°_3/2_)4*f* | ^2^[^3^/_2_] | 1 | 3/2)4f2[3/2]1 | 3*s*^2^3*p*^5^(^2^P°_3/2_)3*d* | ^2^[^3^/_2_]° | 1 | 3/2)3d2[3/2]o1 | 1655.0400 | IR |
| 3*s*^2^3*p*^5^(^2^P°_3/2_)5*s* | ^2^[^3^/_2_]° | 2 | 3/2)5s2[3/2]o2 | 3*s*^2^3*p*^5^(^2^P°_1/2_)4*p* | ^2^[^1^/_2_] | 1 | 1/2)4p2[1/2]1 | 1674.0078 | IR |
| 3*s*^2^3*p*^5^(^2^P°_3/2_)3*d* | ^2^[^3^/_2_]° | 2 | 3/2)3d2[3/2]o2 | 3*s*^2^3*p*^5^(^2^P°_3/2_)4*p* | ^2^[^3^/_2_] | 2 | 3/2)4p2[3/2]2 | 1694.0584 | IR |
| 3*s*^2^3*p*^5^(^2^P°_3/2_)4*f* | ^2^[^7^/_2_] | 3 | 3/2)4f2[7/2]3 | 3*s*^2^3*p*^5^(^2^P°_1/2_)3*d* | ^2^[^5^/_2_]° | 2 | 1/2)3d2[5/2]o2 | 1782.399 | IR |
| 3*s*^2^3*p*^5^(^2^P°_3/2_)5*s* | ^2^[^3^/_2_]° | 1 | 3/2)5s2[3/2]o1 | 3*s*^2^3*p*^5^(^2^P°_1/2_)4*p* | ^2^[^1^/_2_] | 0 | 1/2)4p2[1/2]0 | 2032.256 | IR |
| 3*s*^2^3*p*^5^(^2^P°_3/2_)3*d* | ^2^[^3^/_2_]° | 2 | 3/2)3d2[3/2]o2 | 3*s*^2^3*p*^5^(^2^P°_1/2_)4*p* | ^2^[^3^/_2_] | 2 | 1/2)4p2[3/2]2 | 2062.186 | IR |
| 3*s*^2^3*p*^5^(^2^P°_3/2_)5*p* | ^2^[^5^/_2_] | 3 | 3/2)5p2[5/2]3 | 3*s*^2^3*p*^5^(^2^P°_3/2_)3*d* | ^2^[^3^/_2_]° | 2 | 3/2)3d2[3/2]o2 | 2081.672 | IR |
| 3*s*^2^3*p*^5^(^2^P°_3/2_)3*d* | ^2^[^1^/_2_]° | 1 | 3/2)3d2[1/2]o1 | 3*s*^2^3*p*^5^(^2^P°_1/2_)4*p* | ^2^[^3^/_2_] | 1 | 1/2)4p2[3/2]1 | 2133.871 | IR |
| 3*s*^2^3*p*^5^(^2^P°_3/2_)3*d* | ^2^[^3^/_2_]° | 2 | 3/2)3d2[3/2]o2 | 3*s*^2^3*p*^5^(^2^P°_1/2_)4*p* | ^2^[^1^/_2_] | 1 | 1/2)4p2[1/2]1 | 2154.008 | IR |
| 3*s*^2^3*p*^5^(^2^P°_3/2_)3*d* | ^2^[^1^/_2_]° | 0 | 3/2)3d2[1/2]o0 | 3*s*^2^3*p*^5^(^2^P°_1/2_)4*p* | ^2^[^3^/_2_] | 1 | 1/2)4p2[3/2]1 | 2204.558 | IR |
| 3*s*^2^3*p*^5^(^2^P°_3/2_)3*d* | ^2^[^1^/_2_]° | 1 | 3/2)3d2[1/2]o1 | 3*s*^2^3*p*^5^(^2^P°_1/2_)4*p* | ^2^[^3^/_2_] | 2 | 1/2)4p2[3/2]2 | 2208.321 | IR |
| 3*s*^2^3*p*^5^(^2^P°_3/2_)3*d* | ^2^[^1^/_2_]° | 1 | 3/2)3d2[1/2]o1 | 3*s*^2^3*p*^5^(^2^P°_1/2_)4*p* | ^2^[^1^/_2_] | 1 | 1/2)4p2[1/2]1 | 2313.952 | IR |
| 3*s*^2^3*p*^5^(^2^P°_3/2_)5*p* | ^2^[^5^/_2_] | 3 | 3/2)5p2[5/2]3 | 3*s*^2^3*p*^5^(^2^P°_3/2_)3*d* | ^2^[^7^/_2_]° | 4 | 3/2)3d2[7/2]o4 | 2385.154 | IR |
| 3*s*^2^3*p*^5^(^2^P°_3/2_)3*d* | ^2^[^1^/_2_]° | 0 | 3/2)3d2[1/2]o0 | 3*s*^2^3*p*^5^(^2^P°_1/2_)4*p* | ^2^[^1^/_2_] | 1 | 1/2)4p2[1/2]1 | 2397.306 | IR |

References (with the same reference number in the article)

[32] NIST (National Institute of Standards and Technology, U.S. Department of Commerce) database in https://webbook.nist.gov/chemistry/

Figure Captions

Figure A1. Optical emission spectra of plasma observed in experiment of Ar at 100 Pa. Ar I lines listed in literature and supplemental file is attached in lower part of plot.

Figure A2. Details of node information in Fig. 4(a) using symbol names listed in Tab A1. (a) Upper-left part, (b) lower-left part, (c) upper-right part and (d) lower-right part in Fig. 4(a).

Figure A1

Figure A1. Optical emission spectra of plasma observed in experiment of Ar at 100 Pa. Ar I lines listed in literature and supplemental file is attached in lower part of plot.


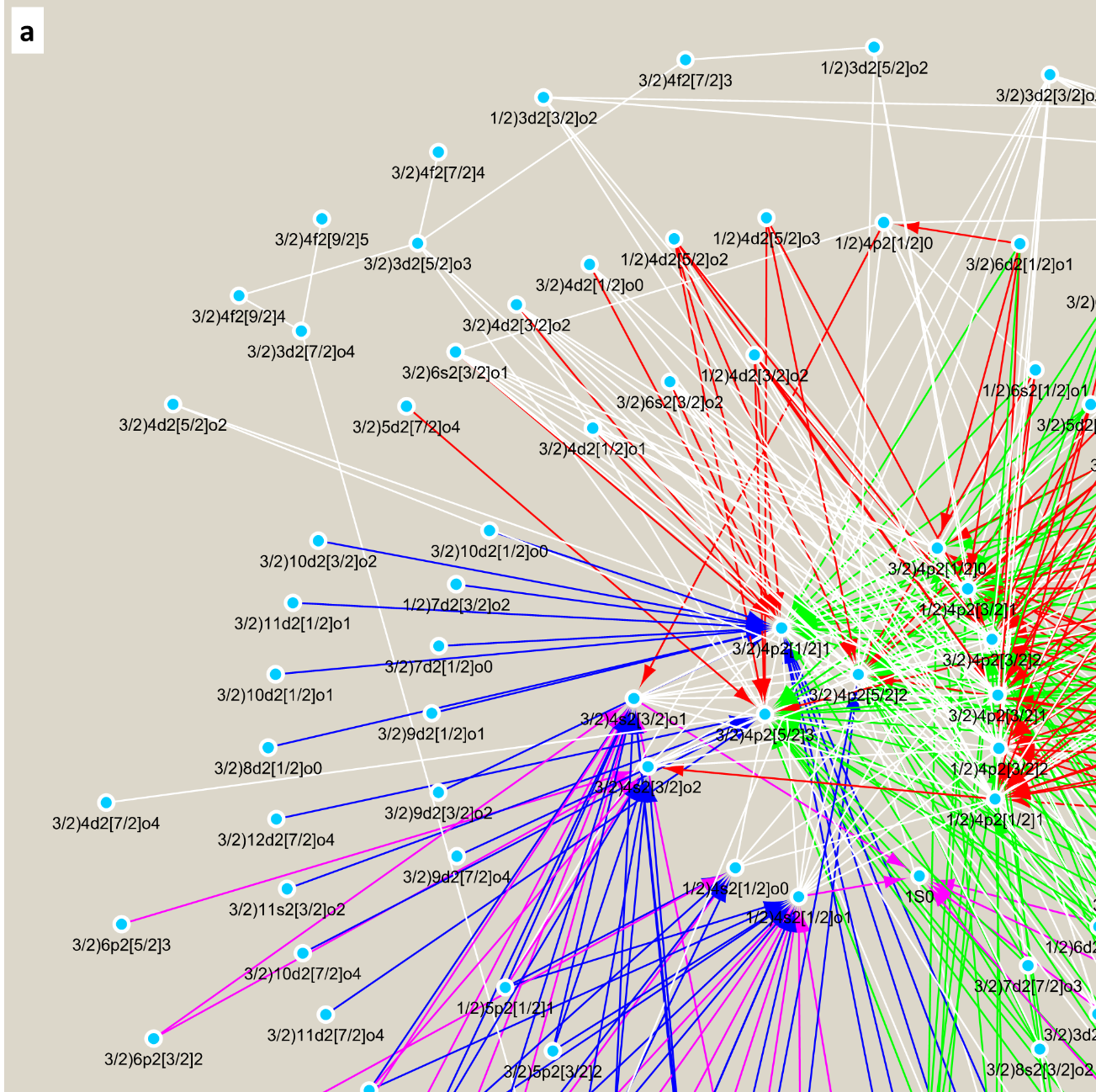


Figure A2(a)

Figure A2. Details of node information in Fig. 4(a) using symbol names listed in Tab A1. (a) Upper-left part, (b) lower-left part, (c) upper-right part and (d) lower-right part in Fig. 4(a).


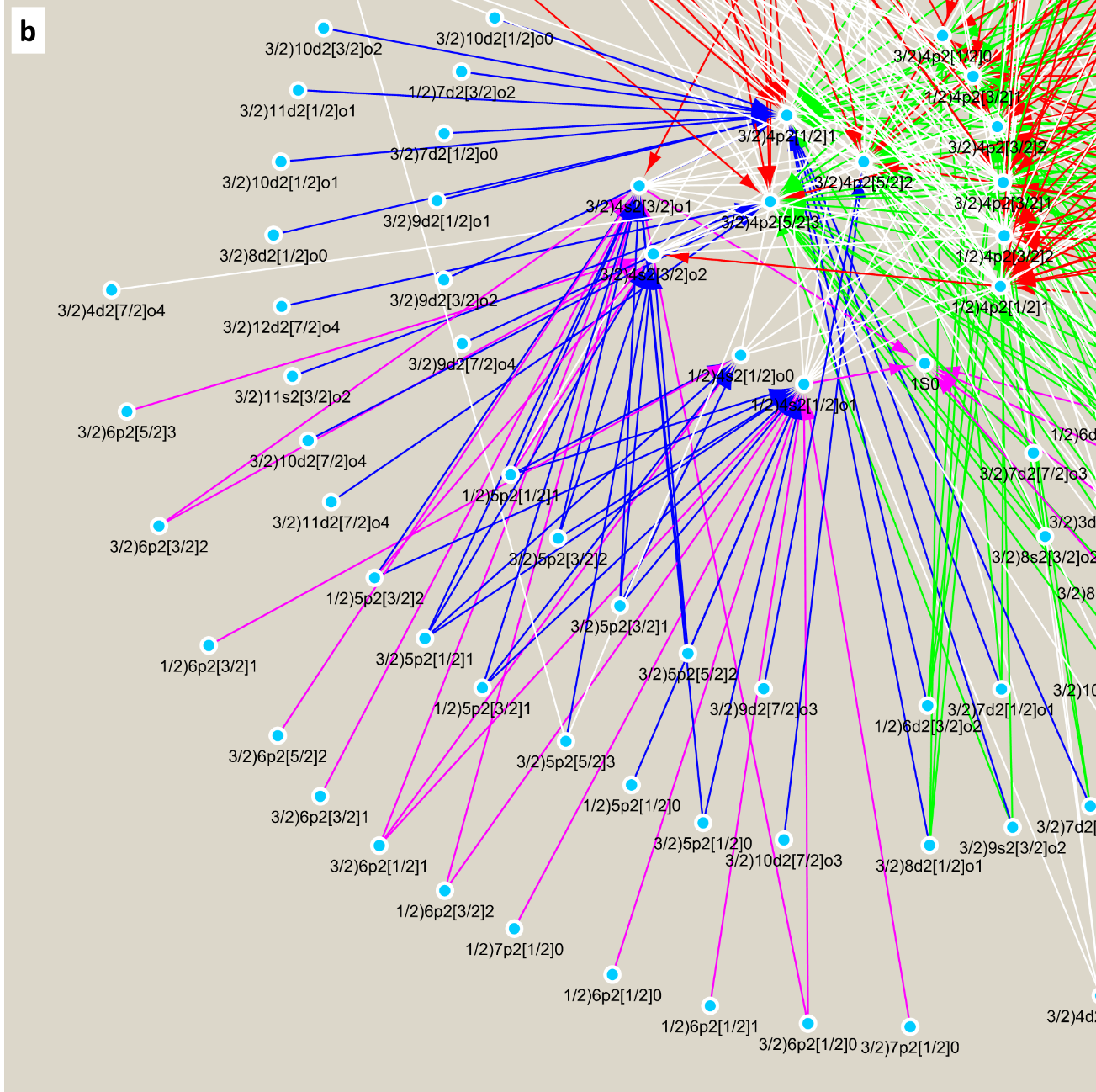


Figure A2(b)

Figure A2. Details of node information in Fig. 4(a) using symbol names listed in Tab A1. (a) Upper-left part, (b) lower-left part, (c) upper-right part and (d) lower-right part in Fig. 4(a).


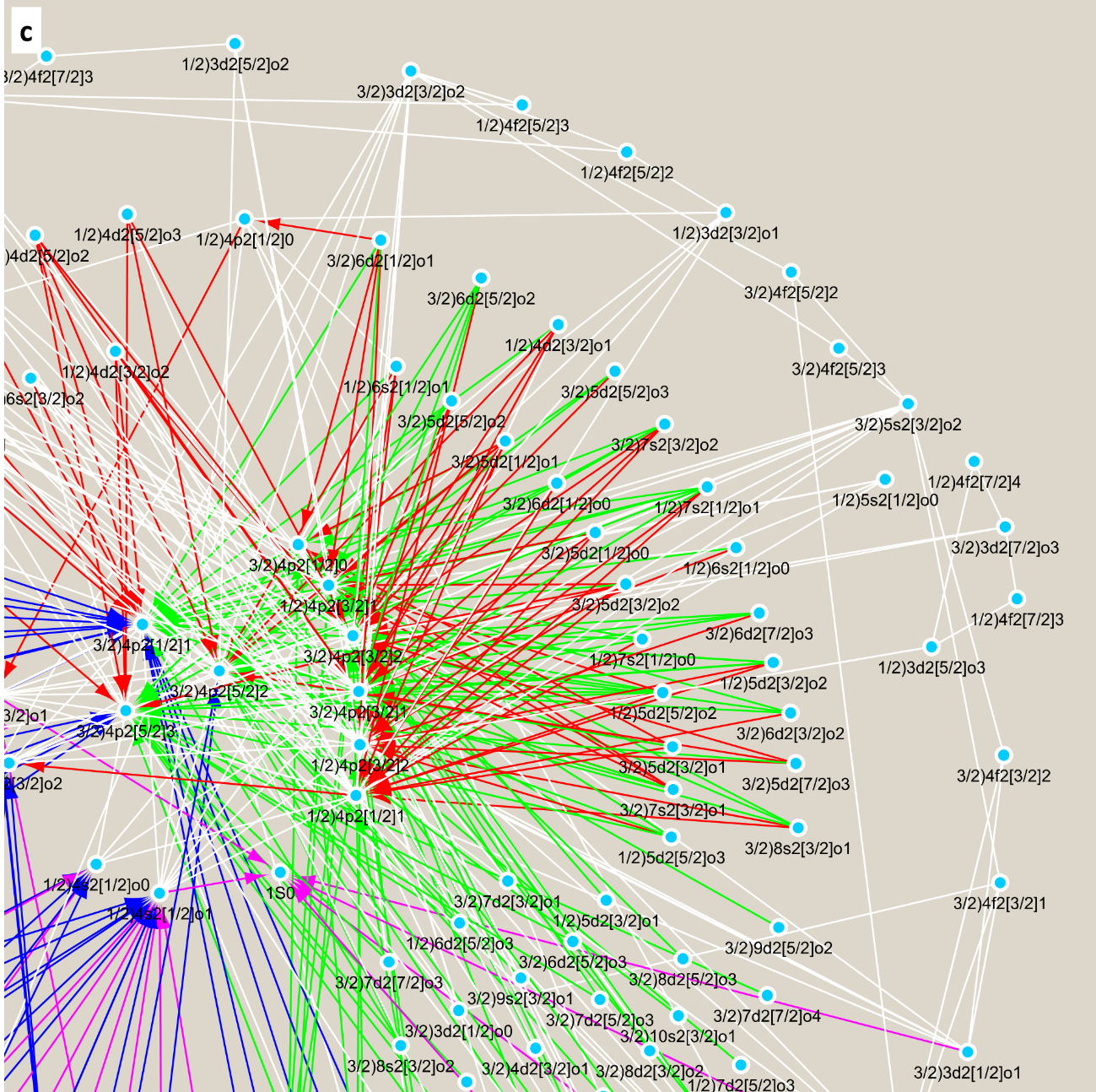


Figure A2(c)

Figure A2. Details of node information in Fig. 4(a) using symbol names listed in Tab A1. (a) Upper-left part, (b) lower-left part, (c) upper-right part and (d) lower-right part in Fig. 4(a).


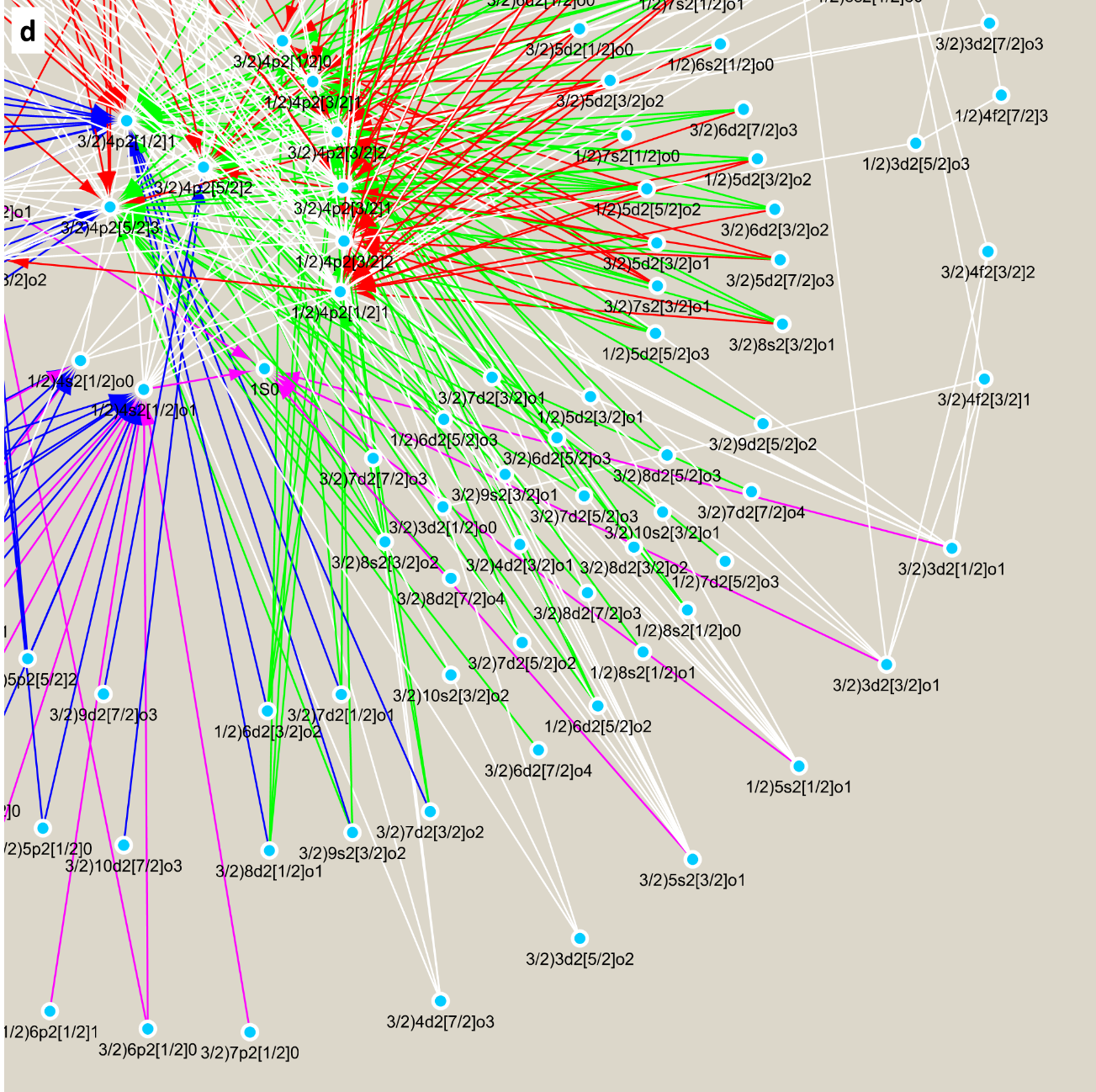


Figure A2(d)

Figure A2. Details of node information in Fig. 4(a) using symbol names listed in Tab A1. (a) Upper-left part, (b) lower-left part, (c) upper-right part and (d) lower-right part in Fig. 4(a).
